# Defining Postinduction Hemodynamic Instability With an Automated Classification Model

**DOI:** 10.1213/ANE.0000000000007315

**Published:** 2024-10-25

**Authors:** Eline Kho, Rogier V. Immink, Bjorn J.P. van der Ster, Ward H. van der Ven, Jimmy Schenk, Markus W. Hollmann, Johan T.M. Tol, Lotte E. Terwindt, Alexander P.J. Vlaar, Denise P. Veelo

**Affiliations:** From the * Department of Anesthesiology, Amsterdam UMC, University of Amsterdam, Amsterdam, the Netherlands; † Department of Intensive Care, Amsterdam UMC, University of Amsterdam, Amsterdam, the Netherlands; ‡ Department of Epidemiology and Data Science, Amsterdam UMC, University of Amsterdam, Amsterdam, the Netherlands; § Laboratory of Experimental Intensive Care and Anesthesiology, Amsterdam UMC, University of Amsterdam, Amsterdam, the Netherlands.

## Abstract

**BACKGROUND:**

Postinduction hypotension (PIH) may be associated with increased morbidity and mortality. In earlier studies, the definition of PIH is solely based on different absolute or relative thresholds. However, the time-course (eg, how fast blood pressure drops during induction) is rarely incorporated, whereas it might represent the hemodynamic instability of a patient. We propose a comprehensive model to distinguish hemodynamically unstable from stable patients by combining blood pressure thresholds with the magnitude and speed of decline.

**METHODS:**

This prospective study included 375 adult elective noncardiac surgery patients. Noninvasive blood pressure was continuously measured between 5 minutes before up to 15 minutes after the first induction agent had been administered. An expert panel rated whether the patient experienced clinically relevant hemodynamic instability or not. Interrater correlation coefficient and intraclass correlation were computed to check for consistency between experts. Next, an automated classification model for clinically relevant hemodynamic instability was developed using mean, maximum, minimum systolic, mean, diastolic arterial blood pressure (SAP, MAP, and DAP, respectively) and their corresponding time course of decline. The model was trained and tested based on the hemodynamic instability labels provided by the experts.

**RESULTS:**

In total 78 patients were classified as having experienced hemodynamic instability and 279 as not. The hemodynamically unstable patients were significantly older (7 years, 95% confidence interval (CI), 4–11, *P* < .001), with a higher prevalence of chronic obstructive pulmonary disease (COPD) (3% higher, 95% CI, 1–8, *P* = .036). Before induction, hemodynamically unstable patients had a higher SAP (median (first–third quartile): 161 (145–175) mm Hg vs 150 (134–166) mm Hg, *P* < .001) compared to hemodynamic stable patients. Interrater agreement between experts was 0.92 (95% CI, 0.89–0.94). The random forest classifier model showed excellent performance with an area under the receiver operating curve (AUROC) of 0.96, a sensitivity of 0.84, and specificity of 0.94.

**CONCLUSIONS:**

Based on the high sensitivity and specificity, the developed model is able to differentiate between clinically relevant hemodynamic instability and hemodynamic stable patients. This classification model will pave the way for future research concerning hemodynamic instability and its prevention.

**KEY POINTS**

**Question:** How can undesirable postinduction blood pressure decreases, defined as hemodynamically instability, be classified with an automated classification model based on blood pressures?

**Findings:** In this prospective study hemodynamically unstable patients were distinguished from a dataset of 375 patients by 15 experts, and implemented to derive a classification model based on blood pressure parameters, resulting in an excellent performance (area under the receiver operating curve (AUROC) of 0.96).

**Meaning:** With the developed automated classification model, clinically relevant hemodynamic instability can be distinguished, paving the for future research concerning hemodynamic instability and its prevention.

Hypotension can develop fast and intense during induction of anesthesia^1–3^ with incidences of postinduction hypotension (PIH) ranging between 9 and 60%.^4–6^ PIH often occurs rapidly, and its correction is frequently initiated too late since the anesthetists’ attention is deviated to other activities during anesthesia induction. The wide distribution of PIH incidence is related to the different definitions of hypotension (eg, absolute or relative thresholds).^7^ The higher the chosen threshold, the higher the incidence of PIH.^8^

Describing hypotension solely based on an absolute or relative threshold seems awkward. First, for awake patients with a high blood pressure (BP), a major drop in BP is required to be classified as hypotensive when relying on an absolute threshold, but only requires a smaller drop when a relative threshold is used. For awake patients with a low BP, the absolute decline in BP, required for classification as hypotensive, is more comparable to the relative threshold. Second, the speed at which BP drops is rarely taken into account. Counteracting hypotension is easier for those patients who merely drift toward a lower BP than those with a fast decline in BP. Thus, patients with a rapid drop in BP while remaining above a BP threshold will not be classified as hypotensive, whereas physicians might feel the urge to intervene due to the hemodynamic instability.

**Figure 1.**
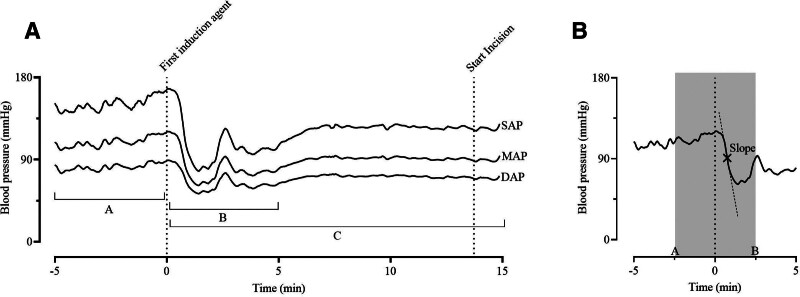
A, Overview of a hemodynamically unstable patient, with the averaged systolic (SAP), mean (MAP), and diastolic blood pressure (DAP). Data were split into three sections: (A) 5 min of blood pressure data before the first induction agent; (B) 5 min of blood pressure data after the first induction agent; and (C) all 15 min of blood pressure data after the first induction agent. From each section, multiple features were derived (Supplemental Digital Content 2, Appendix, Table A1, http://links.lww.com/AA/F123). About 2 min after the administration of the first anesthetics, phenylephrine was administered, probably explaining the sudden increase in blood pressure. B, The 2.5 min before and after induction (gray area) are implemented to derive the maximum slope.

We propose a conversion toward a more comprehensive way to define hemodynamic instability, incorporating the magnitude and speed of BP changes. BP tracings were presented to experts who visually decided which patients developed clinically relevant hypotension during induction and which did not, based on intuitive cognition. With this labeling, an automated classification model was developed and validated.

## METHODS

This prospective single-center study was registered at the Netherlands Trial Register (NL7810) and included adult elective noncardiac surgery patients operated between January 2019 and July 2022 at the Amsterdam University Medical Centers (Amsterdam UMC). Ethical approval for this study was provided by the Institutional Ethics Committee (ref: NL 6748.018.18), and written informed consent was obtained from included patients before participation. Patients were excluded when noninvasive BPs could not be measured, when cardiac arrhythmias, including atrial fibrillation, or abnormal anatomy of the fingers were present. This article adheres to the applicable Consolidated Standards of Reporting Trials (CONSORT) guidelines.

### Blood Pressure Measurements

Beat-to-beat noninvasive BP data were obtained with a ClearSight finger cuff (Edwards Lifesciences). The finger BP was automatically remodeled to a brachial BP. A description of this noninvasive BP measurement technique can be found elsewhere.^9^ Both devices contain the same algorithm that automatically derives beat-to-beat variables such as systolic, mean, and diastolic arterial blood pressure (SAP, MAP, and DAP, respectively).^10^ Measurements started after arrival at the operating theater and lasted until the end of surgery. During measurements, the administration, timing, and dosing of medication were annotated by a trained researcher.

### Preprocessing

Continuous BP data during induction were extracted, starting 5 minutes before, and ending 15 minutes after onset of induction (defined as the moment of administration of the first induction agent). Patients were excluded from analyses when too many BP data were missing (>4 minutes within the 5 minutes before or after start induction, or >8 minutes in the last 10 minutes). MAP, SAP, and DAP data were eliminated in case of unrealistic values (pulse pressure (PP) <10 mm Hg, SAP change >20 mm Hg between consecutive beats, or when the difference between DAP and the 10 enclosing DAP values was >25 mm Hg), and when the heart reference system (HRS) was unaligned (change of >35 cm from start). The remaining beat-to-beat values were interpolated to 1 Hz, followed by a moving average of 45 seconds, and used for further analyses.

### Futurization

Multiple features were extracted from the preprocessed data and used as input for the development of the hemodynamic instability classification model. The 20 minutes of BP measurement were divided into 3 (partly overlapping) sections; 5 minutes before induction, 5 minutes after induction, and 15 minutes after induction (Figure 1A, sections A, B, and C, respectively). For each section, the lowest and highest value (with corresponding time-course), mean, and variance of SAP, MAP, and DAP were determined. PP and the mean negative slope, based on the BP 2.5 minutes before and after induction, Figure 1B, were derived. The first and second deciles of the steepest downward slopes were stored for SAP, MAP, and DAP. Change over time of the variables between the 3 different sections (for example the change in mean SAP; comparing section A to section B) were calculated. In total 98 features were derived (Supplemental Digital Content 1, Supplemental Table 1, http://links.lww.com/AA/F103).

### Classification of Hemodynamic Instability

The derived features are used as the input data for the classification model. This input should be labeled (eg, which patients are hemodynamically unstable and which are stable) so that during the development phase the model is trained to distinguish unstable from stable patients, based on input features. The final classification model will be able to label patients either being hemodynamically unstable or stable, solely based on BP-based features.

**Figure 2.**
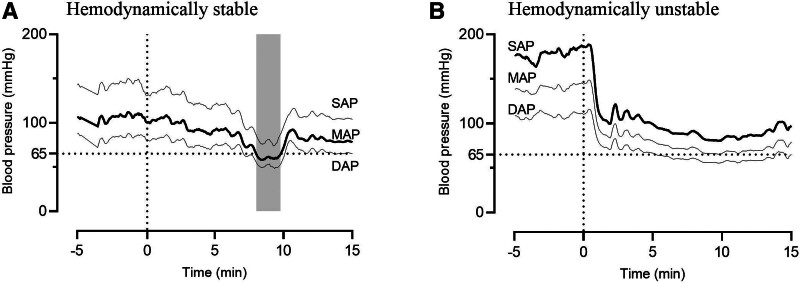
Typical examples of mismatch between a common intraoperative hypotension definition and the PIH definition according to this study. The induction starts at t = 0 min, and the averaged systolic, mean, and diastolic blood pressures are illustrated (SAP, MAP, and DAP, respectively). On the left, a blood pressure tracing is illustrated which we consider not of clinical major interest, as blood pressure declines slowly. However, with the hypotension definition of MAP <65 mm Hg, this patient would be classified as hypotensive. On the right, an obvious hemodynamically unstable patient is illustrated. Here, this patient would have been classified as not having experience hypotension, when applying the hypotension definition of MAP <65 mm Hg or SAP <65 mm Hg. PIH, post-induction hypotension.

Patients who are deemed to have a clinically relevant decrease in BP (eg, a deep or rapid decrease in BP) during induction, that is, severe hemodynamic instability, are defined as being hemodynamically unstable. Patients with a stable or a clinically irrelevant, slowly developing decrease in BP are defined as hemodynamic stable. As highlighted in the introduction, using a fixed threshold may lead to incorrect labeling of patients. For example, implementing a commonly used threshold of MAP <65 mmHg^6,11–14^ may result in the classification of a patient as having experienced hypotension, whereas the reduction in BP is actually very small (Figure 2A). Contrarily, a patient starting off in hypertension can illustrate a major reduction in BP but will not be labeled as having experienced hypotension (Figure 2B).

An expert panel consisting of 15 experienced physicians in this field, working either in the Department of Intensive Care or Anesthesiology at the Amsterdam UMC, individually labeled patients as hemodynamic stable or unstable. Assignment was based on 2 figures, representing the raw noninvasive BP trend, and the 45 seconds averaged SAP, MAP, and DAP tracings (Supplemental Digital Content 2, Supplemental Figures, http://links.lww.com/AA/F104). Exact information such as timings of induction, intubation, nasogastric positioning, administration of anesthetics, and vasopressors were annotated in the figures. We asked the experts to label patients as hemodynamically unstable when they considered the patients’ hemodynamic state unstable or of clinically relevant interest. No strict definition of hemodynamic instability was given at the time of labeling, to capture their intuitive cognition. In total 75 cases were labeled by all experts, of which 25 cases were labeled twice, to check for labeling differences within an expert. When at least 75% of the expert panel agreed, the patient was classified as hemodynamically unstable, and otherwise treated as stable.

### Labelling

Several reliability analyses were performed to assess the labeling of the experts. Correlation coefficients, ranging between 0 and 1, were calculated. A value between 0.50 and 0.75 indicates moderate reliability, 0.75 and 0.90 indicates good reliability, and a value above 0.90 represents excellent reliability.^15^ The correlation coefficients were calculated with a 2-way effect model as the Fleiss kappa was considered too strict. Experts were asked to classify patients as either having experienced hemodynamic instability or not, whereas there might be a gray area in between those 2 groups.

First, the consistency of the experts was determined using the 25 double-labeled patients using intrarater reliability analyses, derived for each expert. Measurements were averaged, using a 2-way mixed effect model, with data treated as single measures.^16,17^ Second, the agreement, or interrater reliability between the experts was determined with the 2-way random and average measures. Third, the average interrater reliability of the labels of 4 experts were compared to that of the other eleven experts. This allowed for determination of whether 4 experts could accurately reflect the integral expert outcome when labeling additional cases. For the interrater reliability analyses, the 2-way random effect model, with average measures, was applied. In the case of good consistency, defined as reliability ≥0.80, these 4 experts were deemed able to label all remaining patients.

### Model

The overall dataset was divided into a training and test set, consisting of 70% and 30% of the samples, respectively. Both sets were normalized by scaling each feature between 0 and 1, and used to derive the best-performing classifier and hyperparameters through a grid search. The tested classifier algorithms were as follows: logistic regression, K-nearest neighbors, support vector machine, and random forest. The models were trained toward the area under the receiver operating curve (AUROC). To solve the imbalanced dataset, the Synthetic Minority Oversampling Technique (SMOTE) was applied to the training dataset.^18^ The percentage correctly identified hemodynamic patients was calculated with the sensitivity, whereas the specificity was calculated to assess the percentage correctly identified hemodynamic unstable and stable patients by the model.

### Secondary Outcomes

The incidence of hemodynamic instability found with the classification model was compared to the incidence of PIH when applying commonly used PIH definitions. First, the incidence of PIH when using a MAP <65 mm Hg as the threshold was checked, and the second implemented definition was a decline in SAP >20% from baseline.

Differences in anesthetics and vasopressors between hemodynamically unstable and stable patients were also investigated. As some anesthetics might result in a decrease in BP, a vasopressor, like phenylephrine, ephedrine, and norepinephrine, can be prophylactically administered. To distinguish vasopressors administered preventatively from those used to treat hypotension, a cutoff time was implemented, defined as the timing of the first rocuronium administration. In case rocuronium was not administered (when the trachea was not intubated), this timing was based on the average time of the rocuronium administration found for the other patients.

### Statistical Analyses

Data were presented as mean with standard deviation (SD) or median (first–third quartile), depending on the distribution. Differences between patients having experienced PIH and patients who did not were assessed with either the unpaired t-test or the Wilcoxon rank sum test for continuous data, and with Fischer’s exact test for categorical data. Preprocessing and futurization was executed using MATLAB (Version 2020b, The Mathworks Inc), reliability analyses with MedCalc (MedCalc Software), and the model development with the Scikit-learn module in Python (Python Software Foundation, version 3.9, Scikit-learn 1.1.3).

## RESULTS

Written informed consent was obtained from 477 patients, of which 38 were excluded due to logistic problems, 7 due to withdrawal of consent, and 57 patients due to missing data. Of the remaining 375 patients, a median of 8.7% (5.8–13.4 first–third quartile), of the SAP/MAP/DAP values were eliminated.

**Table 1.** Patient Characteristics

	Total (n = 375)	Hemodynamically unstable (n = 78)	Hemodynamic stable (n = 297)	P-value
Gender, female/male	188/187	41/37	147/150	.703^a^
Age, y	57 (15)	64 (11)	56 (15)	**<.001** ^b^
Weight, kg	81 (15)	79 (15)	81 (16)	.322^b^
Height, cm	174 (10)	173 (10)	174 (10)	.204^b^
Body mass index, kg·m^-2^	26.6 (4.4)	26.5 (4.3)	26.6 (4.5)	.811^b^
ASA I	68 (18%)	10 (13%)	58 (20%)	.190^a^
II	197 (53%)	46 (59%)	151 (51%)	.206^a^
III	109 (29%)	22 (28%)	87 (19%)	.890^a^
IV	1 (0%)	0	1 (0%)	1.0^a^
History of				
Hypertension	128 (34%)	32 (41%)	96 (32%)	.175^a^
Diabetes Mellitus type II	45 (12%)	12 (15%)	33 (11%)	.323^a^
Atrial fibrillation	19 (5%)	7 (9%)	12 (4%)	.086^a^
COPD	19 (5%)	8 (10%)	11 (4%)	**.036** ^a^
CVA	13 (3%)	3 (4%)	10 (3%)	.738^a^
Type of surgery				
Gastrointestinal	100 (27%)	26 (33%)	74 (25%)	.152^a^
Urological	67 (18%)	15 (19%)	52 (18%)	.741^a^
Gynecological	73 (19%)	13 (17%)	60 (20%)	.525^a^
Orthopedic	33 (9%)	8 (10%)	25 (8%)	.653^a^
Vascular	41 (11%)	5 (6%)	36 (12%)	.220^a^
Ear, nose, throat	20 (5%)	1 (1%)	19 (6%)	.090^a^
Neurological	12 (3%)	3 (4%)	9 (3%)	.719^a^
Plastic	4 (1%)	1 (1%)	3 (1%)	1.0^a^
Other	25 (7%)	6 (8%)	19 (6%)	
Preoperative medication				
Angiotensin-converting enzyme inhibitor	47 (13%)	11 (14%)	36 (12%)	.701^a^
Angiotensin II receptor blocker	50 (13%)	7 (9%)	43 (14%)	.262^a^
Beta blocker	67 (18%)	16 (21%)	51 (17%)	.508^a^
Calcium channel antagonist	63 (17%)	12 (15%)	51 (17%)	.865^a^
Preoperative midazolam	26 (7%)	3 (4%)	23 (8%)	.318^a^

The data are presented as mean with standard deviation, or as number with the corresponding percentage.

Abbreviations: ASA, American Society of Anesthesiologist; COPD, chronic obstructive pulmonary disease; CVA, cerebral vascular accident.

a Fisher exact test.

b Unpaired *t* test.

The data set (188 females vs 187 males) showed a good distribution for age and body mass index, with an average (SD) age of 57 (15) years, and body mass index of 26.6 (4.4) kg·m^-2^ (Table 1). The majority of the population had an American Society of Anesthesiologist (ASA) score of II (53%) or III (29%), and 32% of the patients had chronic hypertension.

### Agreement and Reliability of the Experts

In total 15 experts labeled the patients; 7 with a background in intensive care medicine and 8 with a background in anesthesiology, with a mean (SD) average of 13.4 (8.6) years and 13.4 (6.7) years of experience, respectively. Intrarater reliability of the 15 experts showed good agreement with a correlation of 0.80 (95% confidence interval (CI), 0.61–0.90). Concerning the interrater agreement, comparing experts with each other, an excellent agreement of 0.92 (95% CI, 0.89–0.94) was found. When the average outcome of 4 experts was compared with eleven experts for consistency, a correlation of 0.87 (95% CI, 0.80–0.92) was found, representing good agreement. Based on this, labeling by 4 experts was considered adequate and was applied on the remaining dataset.

### Hemodynamically Unstable versus Stable Patients

In total 21% of the patients were classified as hemodynamically unstable and 79% as stable. When a PIH definition of MAP <65 mm Hg was applied to the dataset, 26% of the patients were classified as hypotensive, of which 44% were also classified as unstable according to the classification model. When the PIH definition was based on a decrease in SAP >20% from baseline, 47% of the patients were classified as hypotensive, of which 32% were also classified as hemodynamically unstable by the classification model.

Based on the experts’ labeling, hemodynamically unstable patients were significantly older (with 7 years, *P* < .001), and had a higher prevalence of chronic obstructive pulmonary disease (COPD; 10% vs 4%, *P* = .036) compared to stable patients. No significant differences were found in other patient characteristics such as height, weight, BMI, history, type of surgery, or preoperative medication. A higher preinduction BP was found in hemodynamically unstable patients (Supplemental Digital Content 1, Supplemental Table 2, http://links.lww.com/AA/F103). Here, SAP was increased (11 mm Hg, *P* < .001), consequently resulting in an increased PP and MAP (10 mm Hg, *P* < .001, and 6 mm Hg, *P* = .015).

### Medication Administration

Comparing the medication administered during the entire induction period, hemodynamically unstable patients received phenylephrine with a higher incidence (18% vs 9%, *P* = .039) and a higher dosage (1.4 vs 1.3 µg·kg^-1^, *P* = .028, Supplemental Digital Content 1, Supplemental Table 3, http://links.lww.com/AA/F103), where no distinction can be made if medication was administered to prevent or correct for instability. Norepinephrine was also administered more often in the hemodynamically unstable group (77% vs 64%, *P* = .039), with a higher dosage (0.47 vs 0.35 µg·kg^-1^·min^-1^, *P* = .007). Likewise, ephedrine boluses were administered more often in hemodynamically unstable patients than in stable patients (23% vs 8%, *P* < .001).

**Table 2.** Confusion Matrix of Test Dataset

		Predicted model output		
		Stable	Unstable	Total
**Experts**	**Stable**	83	5	88
	**Unstable**	4	21	25
	**Total**	87	26	113

**Table 3.** Comparison Between Model and Experts

Amount of times labeled as hemodynamically unstable	Total patients (n = 375)	Test dataset (n = 113)	Model outcome: false classifications
0/4	204	65	1 (2%)
1/4	55	13	1 (8%)
2/4	38	10	3 (30%)
3/4	27	9	3 (33%)
4/4	51	16	1 (6%)

Four experts labeled patients, where at max a patient could be 4 times labeled as being hemodynamically unstable. When labeled at least 3 times as hemodynamically unstable, the patient was classified as being unstable (3/4 and 4/4), otherwise considered stable (0/4, 1/4, 2/4). In the test dataset, the experts classified 88 patients as stable and 25 as stable. From the hemodynamic stable group patients, the model falsely classified 5 patients, whereas for the unstable group, 4 were falsely classified.

**Figure 3.**
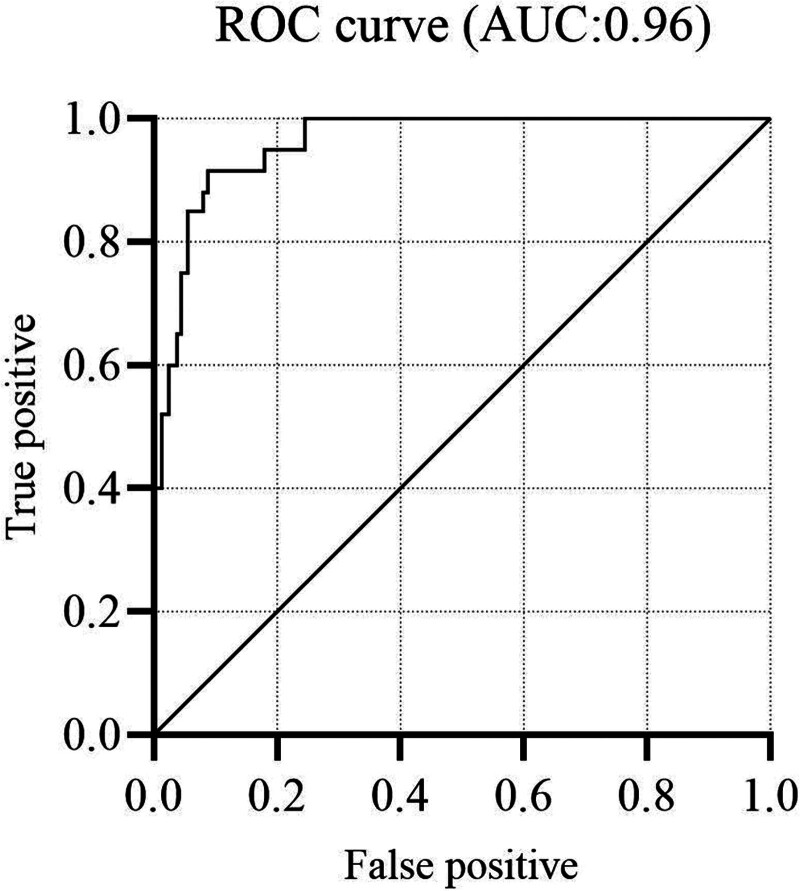
The area under the receiver operating curve is displayed, with sensitivity on the *x*-axis and 1-specificity on the *y*-axis.

To examine whether the treatment/prevention of (early) hypotension had taken place, medication administered until the first rocuronium administration (with a mean (SD) of 140 (94) seconds after the first induction agent) was scrutinized (Supplemental Digital Content 1, Supplemental Table 4, http://links.lww.com/AA/F103). Here, the hemodynamically unstable group received more phenylephrine boluses compared to stable patients (0.608 µg·kg^-1^, *P* = .042), and in more unstable patients continuous norepinephrine administration was started (65% vs 44%, *P* = .001).

### Classification Model

The best-performing model was the random forest classifier, with 50 estimators and a maximum depth of 6 as the optimized parameters (Supplemental Digital Content 1, Supplemental Table 5, http://links.lww.com/AA/F103). Here, an AUROC of 0.98 (SD: 0.01) was found, based on the training set. Other classifiers showed similar results, with an AUROC (SD) of 0.98 (0.01) for K-nearest neighbors, 0.97 (0.02) for logistic regression, and 0.95 (0.03) for the decision tree. Applying the random forest model to the test set resulted in an AUROC of 0.96 (Figure 3), with a sensitivity of 0.84, specificity of 0.94, and accuracy of 0.92. The test dataset consisted of 25 hemodynamically unstable patients, and 88 stable patients. Of those 88 patients, 5 patients were incorrectly identified as being stable, whereas 4 out of 25 unstable patients were incorrectly identified as stable (Table 2). Expert labeling is compared to the outcome of the model in Table 3. Patients for which all experts agreed, that is, labeled as either hemodynamically unstable or stable by all experts, were also often detected by the model; 2% of the stable group and 6% of the unstable group were erroneously labeled. For those patients for whom the experts did not show 100% agreement, the model also showed more erroneous labeling. Here, the model erroneously labeled 8% and 33% of the patients, where 1 expert did not agree with the other 3. 30% of the patients, labeled as hemodynamically unstable by 2 experts and a stable by 2 experts, also showed erroneous labeling.

## DISCUSSION

We present an automated classification model for hemodynamic instability after anesthesia induction. This model is trained on features of the arterial BP and the implemented labels are based on visual differentiation by experts between patients with or without postinduction hemodynamic instability. With this model, clinically relevant hemodynamic (postinduction) instability can effectively be distinguished in an automated way from irrelevant decreases in pressure or normotension. The hemodynamically unstable patients tended to be older, with a higher incidence of COPD, and displayed a higher BP. The classification model showed excellent performance, making automated labeling of these patients feasible in large databases and paving the way for future research in the field of PIH.

The commonly implemented PIH definitions, using BP thresholds, showed a higher incidence (twice as high) of PIH, when compared with the incidence found when focusing on hemodynamic instability. In addition, the patients who were identified as PIH or hemodynamically unstable were not the same.

In previous studies analyzing PIH, the thresholds for intraoperative hypotension, like an absolute value for MAP,^1,6,19–21^ a percentage decline in BP,^13,22^ or a combination of thresholds,^23–25^ were used. However, PIH is caused by different physic and pharmacologic mechanisms compared to intra operative hypotension (IOH). During induction of anesthesia, the instant administration of (bolus) anesthetics, such as propofol and opioids causes arterial and venous vasodilatation and reduces cardiac contractility leading to a reduction in preload and afterload. Moreover, compensatory heart rate increase is limited as baroreflex sensitivity is depressed.^26^ Hence, applying static IOH definitions to assess PIH is insufficient, as it lacks physiological reasoning. In previous studies concerning PIH, its incidence ranges from 9% to 92%,^1,4,6,13,19–25^ making comparison between studies and analyses of risk factors and outcomes challenging.^11,13,24,27,28^ Logically, when applying a very strict definition for PIH, such as MAP <55 mm Hg, the incidence will be lower,^21^ whereas for a more undemanding definition, such as MAP <65 mm Hg, the incidence will be higher.^19^ The proposed classification model does not use the classic threshold definitions but instead implements multiple features from the BP tracing to capture the hypotension of clinical interest; hemodynamic instability. Comparing the outcome of the model to the commonly implemented PIH definitions, we found a lower hemodynamic instability incidence compared to the PIH definitions. The patients the model allocated to be unstable, were not the same patients the PIH definitions found. It appears that our model is not a merge of all PIH definitions, but distinguishes a new, clinically relevant patient category. No other classification for hemodynamic instability after induction has been developed. However, hemodynamic instability and the different ways how to detect is, have been described previously.^29,30^ The focus of this article was the detection of perceived clinically relevant hemodynamic instability post induction based on BP waveform. The model might benefit from other hemodynamic instability detectors, such as electrocardiogram, but it was already sufficient with an AUROC of 0.97.

As our model is trained with labels based on visual interpretation of experts, or their gut feeling, the defined hemodynamic instability entails a more physiological rationale. Distinguishing hemodynamically unstable patients from stable patients with visual BP patterns was feasible with good consensus between experts. The labeling was very strict, as experts were only eligible to label patients as being hemodynamically unstable or not, whereas several patients were within the gray area between the labels. This can result in a lower intrarater reliability of the experts, and a lower interrater reliability between experts concerning the Fleiss’ kappa. Therefore, we implemented the 2-way random effect model to assess the interrater reliability, as this test is based on continuous values, partly omitting the 2 label restrictions. With the classification model, we found that most of the patients labeled as hemodynamically unstable by all experts were correctly identified by the model. Vice versa, concerning the labeled hemodynamic stable patients, the model classified most of those patients correctly as well. When experts did not unanimously agree, the classification model also showed more false positive/negatives. However, to strengthen the model, external validation with experts of other hospitals is desirable. This model could aid in further research in predicting, and as a result preventing, hemodynamic instability and possible related adverse outcomes.

Hemodynamically unstable patients showed an association with increased age, incidence of COPD, and a high SAP based on preinduction BP. These associations were also found in other studies, reporting associations with age,^4,27^ sex, weight, type 2 diabetes,^13^ MAP^4,25,31,32^ and SAP.^4,13,27^ Of interest is the reported inconsistency in the association of preinduction MAP or SAP with the occurrence of PIH. One study showed that a MAP <70 mm Hg was found positively related to PIH incidence,^4^ whereas other studies found a relation with an increased MAP.^23,25,32^ For SAP, a similar inconsistency was found, where both a lower^27^ and higher^13^ SAP were associated with PIH. Both studies included similar patients, and used a comparable preinduction period to measure MAP. These conflicting results may be explained by the different definitions of hypotension used, but also by the difference in premedication or the different stages of anxiety of the patients. Focusing on our current study, the higher BP in the hemodynamically unstable group might be induced by the physician; we found a higher incidence of norepinephrine administration in this group. This might suggest that physicians anticipated these patients to become hemodynamically unstable.

A limitation of this study is the lack of a strict induction protocol, which resulted in differences in the number and type of induction agents, making comparison more difficult. However, based on the induction time period until the first rocuronium administration, hemodynamically unstable patients received more (incidence or dosage) vasopressors (norepinephrine and phenylephrine) compared to stable patients. This suggests that, despite the physician suspected or perceived hypotension to occur in the hemodynamically unstable group, the physician was not able to prevent it or did not treat aggressively enough.

The second limitation concerns the recording of noninvasive BP and the embedded algorithm underlying the calculation of beat-to-beat values. When BP is very low, this may result in a small difference between systolic and diastolic pressure. The algorithm is not always capable to detect these low pressures. Regarding our study, this could have had an effect on the number of hemodynamically unstable patients, as these specific patients were removed from the dataset due to too much missing data. However, this occurrence was rarely seen in our population and would not affect our model, but merely reduce the dataset.

In conclusion, we developed an automated classification model to distinguish between the presence and absence of clinically relevant hemodynamic instability, based on BP visualization. By creating this model, hemodynamically unstable patients can now automatically be classified, contributing to further research concerning hemodynamic instability, its risk factors, and its prevention.

## ACKNOWLEDGMENTS

We would like to thank the following for their expertise concerning postinduction hypotension: P. R. Tuinman, S. F. van Wonderen, W. K. Lagrand, J. S. J. Raasveld, J. Schuurmans, P. R. Wynandts, M. C. Reuland, D. A. Dongelmans, D. M. P. van Meenen, T. G. V. Cherpanath, and H. Hermanns from the departments of Anesthesiology and Intensive Care, Amsterdam UMC, the Netherlands.

## DISCLOSURES

**Conflicts of Interest:** A. P.J. Vlaar, D. P. Veelo, and R. V. Immink have received grants and consultancy fees from Edwards Lifescience, and A. P.J. Vlaar and D. P. Veelo also from Philips Medical BV. Ma. W. Hollmann reports having received grants and consultancy fees from PAION, Medical Developments and IDD Pharma. No other authors declared Conflicts of Interest. **Funding:** None. **This manuscript was handled by:** Thomas M. Hemmerling, MSc, MD, DEAA.

## Supplementary Material


