# Supplementary material for: Defining Postinduction Hemodynamic Instability With an Automated Classification Model
Source: Anesth Analg. 2024 Oct 25;140(2):444–52. doi: 10.1213/ANE.0000000000007315 (PMC11687939; doi:10.1213/ANE.0000000000007315)
Supplement: Supplementary file 2 [file ane-140-444-s002.docx]

**APPENDIX**

| Table 1. Overview of blood pressure features implemented in the model   \|  \| Mean \| Maximum \| time \| Minimum \| time \| Variance \| 0.1 Decile \| 0.2 Decile \| Slope \| Total \| \| --- \| --- \| --- \| --- \| --- \| --- \| --- \| --- \| --- \| --- \| --- \| \| SAP \| A, B, C,  ∆AB,∆AC \| A, B, C,  ∆AB,∆AC \| B,C \| A, B, C,  ∆AB,∆AC \| B, C \| A, B, C, ∆AB,∆AC \| A, B, C \| A, B, C \| AB \| 31 \| \| MAP \| A, B, C,  ∆AB,∆AC \| A, B, C,  ∆AB,∆AC \| B,C \| A, B, C,  ∆AB,∆AC \| B, C \| A, B, C, ∆AB,∆AC \| A, B, C \| A, B, C \| AB \| 31 \| \| DAP \| A, B, C,  ∆AB,∆AC \| A, B, C,  ∆AB,∆AC \| B,C \| A, B, C,  ∆AB,∆AC \| B, C \| A, B, C, ∆AB,∆AC \| A, B, C \| A, B, C \| AB \| 31 \| \| PP \| A, B, C,  ∆AB,∆AC \|  \|  \|  \|  \|  \|  \|  \|  \| 5 \| \| Total \| 20 \| 15 \| 6 \| 15 \| 6 \| 15 \| 9 \| 9 \| 3 \| 98 \|   Features are calculated from the systolic, mean, and diastolic blood pressure (SAP, MAP, DAP) and the pulse pressure (PP), in sections A, B, and C (Figure 1). Additionally, differences in the features between sections (∆AB or ∆AC) are calculated. The slope is calculated in blood pressure data 2.5 minutes before (last half section A) and after (first half section B) the first induction agent. Per patient in total 98 features are calculated and used for the model. |
| --- | --- | --- | --- | --- | --- | --- | --- | --- | --- | --- | --- | --- | --- | --- | --- | --- | --- | --- | --- | --- | --- | --- | --- | --- | --- | --- | --- | --- | --- | --- | --- | --- | --- | --- | --- | --- | --- | --- | --- | --- | --- | --- | --- | --- | --- | --- | --- | --- | --- | --- | --- | --- | --- | --- | --- | --- | --- | --- | --- | --- | --- | --- | --- | --- | --- | --- |

| Table 2. Hemodynamic parameters based on the 5 minutes prior to start induction   \|  \| \| \| \| Hemodynamically unstable \| Hemodynamic stable \| p-value \| \| --- \| --- \| --- \| --- \| --- \| --- \| --- \| \| Mean \| *SAP (mmHg)* \| \| \| 161 (145-175) \| 150 (134-166) \| **<0.001** \| \| MAP (mmHg) \| \| \| \| 116 (106-127) \| 110 (101-122) \| **0.015** \| \| DAP (mmHg) \| \| \| \| 88 (79-95) \| 86 (78-93) \| 0.280 \| \| PP (mmHg) \| \| \| \| 73 (62-82) \| 63 (53-76) \| **<0.001** \| \| Max \| \| *SAP (mmHg)* \| \| 174 (157-189) \| 161 (143-178) \| **<0.001** \| \| MAP (mmHg) \| \| \| \| 125 (115-140) \| 119 (109-132) \| **0.009** \| \| DAP (mmHg) \| \| \| \| 96 (88-104) \| 94 (86-102) \| 0.366 \| \| PP (mmHg) \| \| \| \| 80 (69-90) \| 71 (90-83) \| **<0.001** \| \| Min \| \| *SAP (mmHg)* \| \| 146 (133-160) \| 136 (119-153) \| **<0.001** \| \| MAP (mmHg) \| \| \| \| 105 (94-116) \| 101 (91-111) \| **0.025** \| \| DAP (mmHg) \| \| \| \| 80 (72-86) \| 78 (70-85) \| 0.258 \| \| PP (mmHg) \| \| \| \| 63 (54-72) \| 53 (42-66) \| **<0.001** \| \| Variance \| \| \| *SAP (mmHg)* \| 39 (21-66) \| 34 (17-58) \| 0.097 \| \| MAP (mmHg) \| \| \| \| 21 (14-36) \| 18 (11-32) \| 0.120 \| \| DAP (mmHg) \| \| \| \| 12 (8-21) \| 12 (7-23) \| 0.793 \| \| 0.1 Decile \| \| \| *SAP (%)* \| -0.37 (-0.44—0.31) \| -0.36 (-0.44—0.29) \| 0.360 \| \| MAP (%) \| \| \| \| -0.30 (-0.37—0.23) \| -0.29 (-0.36—0.23) \| 0.210 \| \| DAP (%) \| \| \| \| -0.24 (-0.31—0.19) \| -0.25 (-0.32-0.19) \| 0.834 \| \| 0.2 Decile \| \| \| *SAP (%)* \| -0.29 (-0.35—0.24) \| -0.27 (-0.34—0.22) \| 0.743 \| \| MAP (%) \| \| \| \| -0.23 (-0.28—0.18) \| -0.22 (-0.27—0.17) \| 0.286 \| \| DAP (%) \| \| \| \| -0.17 (-0.23—0.13) \| -0.19 (-0.24—0.15) \| 0.213 \| \| Overview of the hemodynamic parameters of hemodynamically unstable and stable patients. Data is presented as median (1^st^ -3^rd^ quartile). Significance was calculated with the Wilcoxon rank sum test. \| \| \| \| \| \| \| |
| --- | --- | --- | --- | --- | --- | --- | --- | --- | --- | --- | --- | --- | --- | --- | --- | --- | --- | --- | --- | --- | --- | --- | --- | --- | --- | --- | --- | --- | --- | --- | --- | --- | --- | --- | --- | --- | --- | --- | --- | --- | --- | --- | --- | --- | --- | --- | --- | --- | --- | --- | --- | --- | --- | --- | --- | --- | --- | --- | --- | --- | --- | --- | --- | --- | --- | --- | --- | --- | --- | --- | --- | --- | --- | --- | --- | --- | --- | --- | --- | --- | --- | --- | --- | --- | --- | --- | --- | --- | --- | --- | --- | --- | --- | --- | --- | --- | --- | --- | --- | --- | --- | --- | --- | --- | --- | --- | --- | --- | --- | --- | --- | --- | --- | --- | --- | --- | --- | --- | --- | --- | --- | --- | --- | --- | --- | --- | --- | --- | --- | --- | --- | --- | --- | --- | --- | --- | --- | --- | --- | --- | --- | --- | --- | --- | --- | --- | --- | --- | --- | --- | --- | --- | --- | --- | --- | --- | --- | --- | --- | --- | --- |

| Table 3. Medication administration during induction   \|  \| Patients receiving medication \| \| \| \| Average dosage \| \| \| \| \| \| --- \| --- \| --- \| --- \| --- \| --- \| --- \| --- \| --- \| --- \| \|  \| *Total (n:375)* \| *Unstable (n:78)* \| *Stable (n:297)* \| *p-value^a^* \| *Total* \| *Unstable* \| *Stable* \| *Mean difference* \| *p-value^b^* \| \| Bolus \|  \|  \|  \|  \|  \|  \|  \|  \|  \| \| Propofol (mg·kg^-1^) \| 278 (74%) \| 63 (81%) \| 215 (72%) \| 0.148 \| 1.6 (1.1-2.1) \| 1.6 (1.3-2.0) \| 1.6 (1.1-2.1) \| -0.012 \| 0.901 \| \| Remifentanil (µg·kg^-1^) \| 2 (1%) \| 0 \| 2 (1%) \| - \| 0.30 (0.29-0.31) \| - \| 0.30 (0.29-0.31) \| - \| - \| \| Sufentanil (µg·kg^-1^) \| 294 (78%) \| 61 (78%) \| 232 (78%) \| 0.878 \| 0.27 (0.22-0.33) \| 0.28 (0.23-0.33) \| 0.27 (0.22-0.33) \| -0.005 \| 0.693 \| \| Morphine (mg·kg^-1^) \| 31 (8%) \| 7 (9%) \| 24 (8%) \| 0.818 \| 0.09 (0.06-0.13) \| 0.11 (0.09-0.15) \| 0.08 (0.05-0.12) \| 0.025 \| 0.203 \| \| Ephedrine (µg·kg^-1^) \| 41 (11%) \| 18 (23%) \| 23 (8%) \| **<0.001** \| 70 (62-83) \| 70 (61-80) \| 70 (63-96) \| -0.011 \| 0.217 \| \| Esketamine (mg·kg^-1^) \| 223 (59%) \| 49 (63%) \| 174 (59%) \| 0.520 \| 0.25 (0.21-0.30) \| 0.26 (0.22-0.31) \| 0.25 (0.20-0.30) \| 0.022 \| 0.143 \| \| Phenylephrine (µg·kg^-1^) \| 41 (11%) \| 14 (18%) \| 27 (9%) \| **0.039** \| 1.3 (1.0-1.6) \| 1.4 (1.0-2.1) \| 1.3 (1.0-1.5) \| 0.425 \| **0.028** \| \| Midazolam (µg·kg^-1^) \| 10 (3%) \| 4 (5%) \| 6 (2%) \| 0.227 \| 30 (20-37) \| 34 (26-42) \| 22 (20-30) \| 0.005 \| 0.602 \| \| Glycopyronium (µg·kg^-1^) \| 7 (2%) \| 1 (1%) \| 6 (2%) \| 1.0 \| 29 (26-29) \| 24 (24-24) \| 29 (27-29) \| - \| 1.0 \| \| Continuous administration \|  \|  \|  \|  \|  \|  \|  \|  \|  \| \| Propofol (mg·kg^-1^·hr^-1^) \| 204 (54%) \| 45 (58%) \| 159 (54%) \| 0.526 \| 1.6 (1.3-1.9) \| 1.6 (1.2-1.9) \| 1.6 (1.3-1.9) \| 0.023 \| 0.811 \| \| Propofol TCI \| 100 (27%) \| 16 (21%) \| 84 (28%) \| 0.196 \| 0.021 (0.01-0.23) \| 0.027 (0.012-0.160) \| 0.021 (0.011-0.229) \| -0.010 \| 0.915 \| \| Remifentanil (µg·kg^-1^·hr^-1^) \| 86 (23%) \| 17 (22%) \| 69 (23%) \| 0.880 \| 5.5 (3.2-6.8) \| 6.8 (5.9-7.0) \| 5.2 (2.8-6.4) \| 1.5 \| **0.042** \| \| Remifentanil TCI \| 3 (1%) \| 0 \| 3 (1%) \| - \| 0.026 (0.017-0.031) \| - \| 0.026 (0.017-0.031) \| - \| - \| \| Sufentanil (µg·kg^-1^·hr^-1^) \| 12 (3%) \| 3 (4%) \| 9 (3%) \| 0.719 \| 0.053 (0.021-0.092) \| 0.075 (0.042-0.155) \| 0.044 (0.016-0.086) \| -0.108 \| 0.716 \| \| Esketamine (µg·kg^-1^·hr^-1^) \| 5 (1%) \| 0 \| 5 (2%) \| - \| 21 (11-26) \| - \| 21 (11-26) \| - \| - \| \| Norepinephrine (µg·kg^-1^·min^-1^) \| 250 (67%) \| 60 (77%) \| 190 (64%) \| **0.032** \| 0.37 (0.25-0.59) \| 0.47 (0.31-0.63) \| 0.35 (0.23-0.54) \| 0.105 \| **0.007** \| \| Data is presented as number (%), or as median (1^st^ -3^rd^ quartile). The mean difference in dosage between the hemodynamically unstable and stable patients is presented for clarification. TCI: Target-controled infusion.  ^a^ Fisher’s exact test  ^b^ Wilcoxon rank sum test \| \| \| \| \| \| \| \| \| \| |
| --- | --- | --- | --- | --- | --- | --- | --- | --- | --- | --- | --- | --- | --- | --- | --- | --- | --- | --- | --- | --- | --- | --- | --- | --- | --- | --- | --- | --- | --- | --- | --- | --- | --- | --- | --- | --- | --- | --- | --- | --- | --- | --- | --- | --- | --- | --- | --- | --- | --- | --- | --- | --- | --- | --- | --- | --- | --- | --- | --- | --- | --- | --- | --- | --- | --- | --- | --- | --- | --- | --- | --- | --- | --- | --- | --- | --- | --- | --- | --- | --- | --- | --- | --- | --- | --- | --- | --- | --- | --- | --- | --- | --- | --- | --- | --- | --- | --- | --- | --- | --- | --- | --- | --- | --- | --- | --- | --- | --- | --- | --- | --- | --- | --- | --- | --- | --- | --- | --- | --- | --- | --- | --- | --- | --- | --- | --- | --- | --- | --- | --- | --- | --- | --- | --- | --- | --- | --- | --- | --- | --- | --- | --- | --- | --- | --- | --- | --- | --- | --- | --- | --- | --- | --- | --- | --- | --- | --- | --- | --- | --- | --- | --- | --- | --- | --- | --- | --- | --- | --- | --- | --- | --- | --- | --- | --- | --- | --- | --- | --- | --- | --- | --- | --- | --- | --- | --- | --- | --- | --- | --- | --- | --- | --- | --- | --- | --- | --- | --- | --- | --- | --- | --- | --- | --- | --- | --- | --- | --- | --- | --- |

| Table 4. Medication administration during induction until the first rocuronium administration |
| --- |
| \|  \| Patients receiving medication \| \| \| \| Average dosage \| \| \| \| \| \| --- \| --- \| --- \| --- \| --- \| --- \| --- \| --- \| --- \| --- \| \|  \| *Total (n:375)* \| *Unstable (n:78)* \| *Stable*  *(n:297)* \| *p-value^a^* \| *Total* \| *Unstable* \| *Stable* \| *Mean difference* \| *p-value^b^* \| \| Bolus \|  \|  \|  \|  \|  \|  \|  \|  \|  \| \| Propofol (mg·kg^-1^) \| 268 (71%) \| 62 (79%) \| 206 (69%) \| 0.091 \| 1.6 (1.2-2.0) \| 1.6 (1.3-2.0) \| 1.6 (1.1-2.1) \| -0.041 \| 0.675 \| \| Remifentanil (µg·kg^-1^) \| 0 \| - \| - \| - \| - \| - \| - \| - \| - \| \| Sufentanil (µg·kg^-1^) \| 290 (77%) \| 62 (79%) \| 228 (77%) \| 0.652 \| 0.27 (0.22-0.33) \| 0.28 (0.23-0.33) \| 0.27 (0.22-0.33) \| -0.006 \| 0.644 \| \| Morphine (mg·kg^-1^) \| 14 (4%) \| 3 (4%) \| 11 (4%) \| 1.0 \| 0.12 (0.07-0.15) \| 0.14 (0.10-0.15) \| 0.12 (0.06-0.14) \| 0.018 \| 0.612 \| \| Ephedrine (µg·kg^-1^) \| 5 (1%) \| 0 \| 5 (2%) \| - \| 62 (56-82) \| **-** \| 62 (56-82) \| - \| - \| \| Esketamine (mg·kg^-1^) \| 205 (55%) \| 48 (62%) \| 157 (53%) \| 0.201 \| 0.25 (0.21-0.30) \| 0.27 (0.22-0.31) \| 0.25 (0.20-0.30) \| 0.020 \| 0.195 \| \| Phenylephrine (µg·kg^-1^) \| 14 (4%) \| 2 (3%) \| 12 (4%) \| 0.743 \| 1.3 (1.0-1.5) \| 1.8 (1.5-2.1) \| 1.2 (0.9-1.5) \| 0.608 \| **0.042** \| \| Midazolam (µg·kg^-1^) \| 10 (3%) \| 4 (5%) \| 6 (2%) \| 0.227 \| 27 (20-37) \| 34 (26-42) \| 22 (20-30) \| 0.005 \| 0.602 \| \| Glycopyronium (µg·kg^-1^) \| 4 (1%) \| - \| 4 (1%) \| - \| 28 (27-30) \| - \| 28 (27-30) \| - \| - \| \| Continuous administration \|  \|  \|  \|  \|  \|  \|  \|  \|  \| \| Propofol (mg·kg^-1^·hr^-1^) \| 143 (38%) \| 33 (42%) \| 110 (37%) \| 0.433 \| 1.7 (1.5-2.0) \| 1.7 (1.5-2.0) \| 1.7 (1.4-1.9) \| -0.008 \| 0.939 \| \| Propofol TCI \| 89 (24%) \| 13 (17%) \| 76 (26%) \| 0.134 \| 0.016 (0.011-0.180) \| 0.014 (0.011-0.067) \| 0.018 (0.011-0.207) \| -0.017 \| 0.831 \| \| Remifentanil (µg·kg^-1^·hr^-1^) \| 79 (21%) \| 17 (22%) \| 62 (21%) \| 0.877 \| 5.7 (3.8-6.9) \| 6.8 (5.9-7.0) \| 5.3 (3.8-6.6) \| 1.1 \| 0.121 \| \| Remifentanil TCI \| 3 (1%) \| 0 \| 3 (1%) \| - \| 0.026 (0.017-0.031) \| - \| 0.026 (0.017-0.031) \| - \| - \| \| Sufentanil (µg·kg^-1^·hr^-1^) \| 7 (2%) \| 2 (3%) \| 5 (2%) \| 0.639 \| 0.079 (0.065-0.162) \| 0.128 (0.075-0.181) \| 0.079 (0.047-0.450) \| -0.219 \| 0.667 \| \| Esketamine (µg·kg^-1^·hr^-1^) \| 0 \| - \| - \| - \| - \| - \| - \| - \| - \| \| Norepinephrine (µg·kg^-1^·min^-1^) \| 183 (49%) \| 51 (65%) \| 132 (44%) \| **0.001** \| 0.46 (0.31-0.63) \| 0.51 (0.36-0.65) \| 0.44 (0.31-0.62) \| 0.082 \| 0.058 \| \| Data is presented as number (%), or as median (1^st^ -3^rd^ quartile). The mean difference in dosage between the hemodynamically unstable and stable patients is presented for clarification. TCI: Target-controled infusion.  ^a^ Fisher’s exact test  ^b^ Wilcoxon rank sum test \| \| \| \| \| \| \| \| \| \| |

| Table 5. Optimized parameters of the final classification model.   \| Parameter \| Value \| \| \| \| \| --- \| --- \| --- \| --- \| --- \| \| Classifier \| Random forest \| \| \| \| \| Estimator \| 50 \| \| \| \| \| Depth \| 6 \| \| \| \| \| Best features,  in arbitrary order (n:40) \| *SAP* \| *MAP* \| *DAP* \| *PP* \| \| Mean \| Section B, ∆AB, ∆AC \| Section B, ∆AB, ∆AC \| Section B, ∆AB, ∆AC \| ∆AB, ∆AC \| \| Minimum \| Section B, C,  ∆AB, ∆AC \| Section B, C,  ∆AB, ∆AC \| Section B, C, ∆AB, ∆AC \|  \| \| Minimum time \| Section C \| Section C \| Section C \|  \| \| Variance \| Section B, C,  ∆AB \| Section B, ∆AB \| Section B, ∆AB \|  \| \| 0.1 Decile \| Section B \| Section B \|  \|  \| \| 0.2 Decile \| Section B \| Section B \|  \|  \| \| Slope \| Section AB \| Section AB \| Section AB \|  \| |
| --- | --- | --- | --- | --- | --- | --- | --- | --- | --- | --- | --- | --- | --- | --- | --- | --- | --- | --- | --- | --- | --- | --- | --- | --- | --- | --- | --- | --- | --- | --- | --- | --- | --- | --- | --- | --- | --- | --- | --- | --- | --- | --- | --- | --- | --- | --- | --- | --- | --- | --- | --- | --- | --- | --- | --- | --- | --- | --- | --- | --- |
