# Supplementary material for: Defining Postinduction Hemodynamic Instability With an Automated Classification Model
Source: Anesth Analg. 2024 Oct 25;140(2):444–52. doi: 10.1213/ANE.0000000000007315 (PMC11687939; doi:10.1213/ANE.0000000000007315)
Supplement: Supplementary file 3 [file ane-140-444-s003.pdf]

Supplemental Figures. The blood pressure tracings of the 75 patients, reviewed by the 15 experts. In the upper panel, the averaged systolic, mean, and arterial blood pressure are presented. Additionally, anesthetics during the induction period are given. The raw blood pressure tracing can be found in the lower panel, with the systolic, mean, and arterial blood pressure in red, yellow and purple respectively.

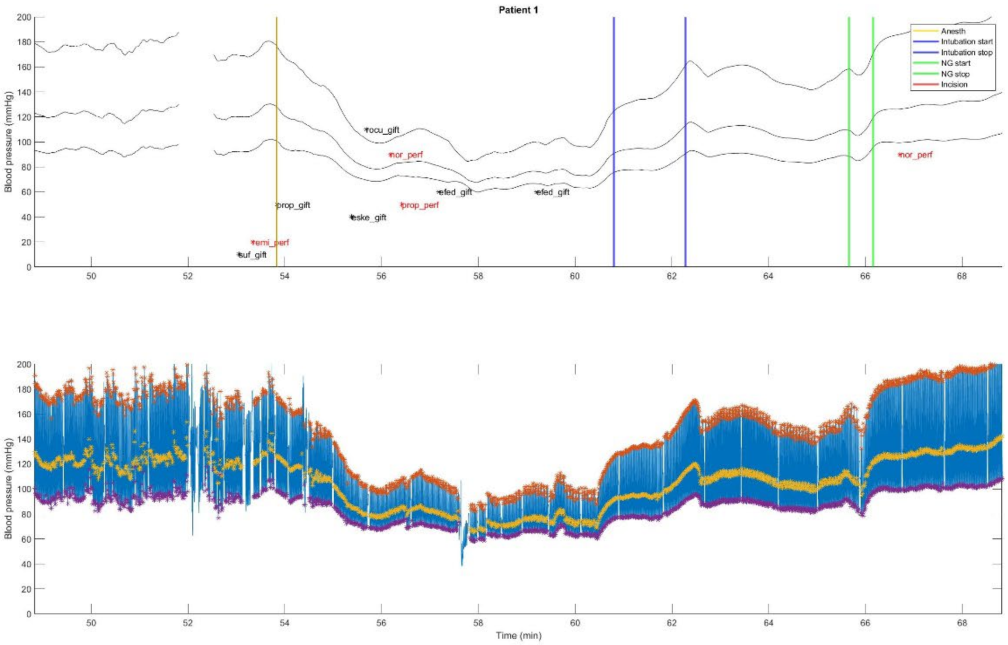
Patient 1
Anesth
Intubation start
Intubation stop
NG start
NG stop
Incision
rocu_gift
nor_perf
efed_gift
efed_gift
prop_gift
eske_gift
prop_perf
remi_perf
suf_gift
nor_perf
Blood pressure (mmHg)
Blood pressure (mmHg)
Time (min)

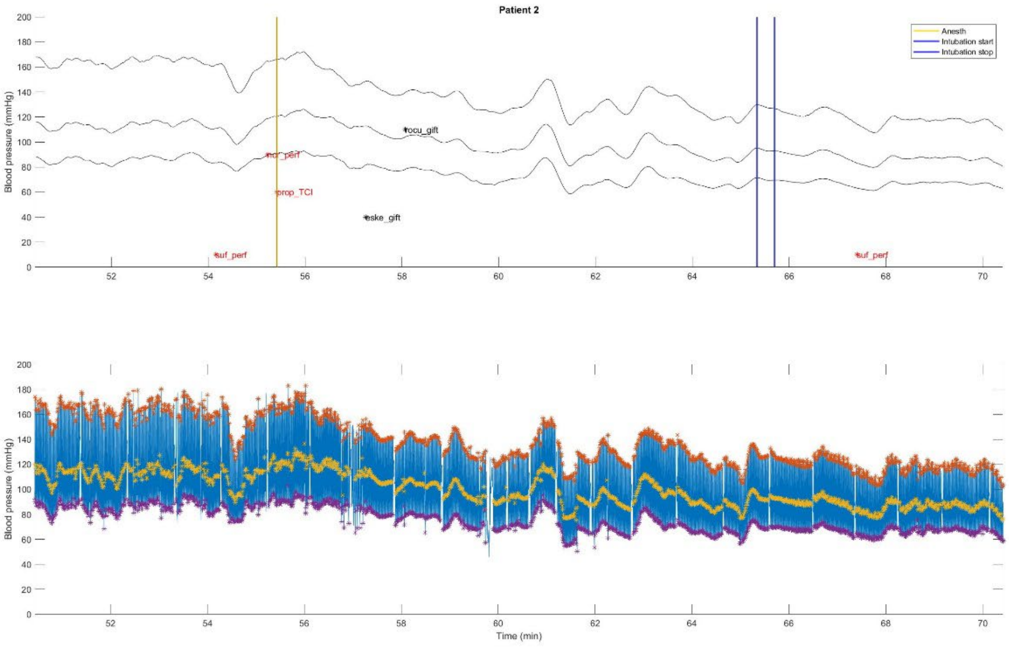
Patient 2
Anesth
Intubation start
Intubation stop
rocu_gift
nor_perf
prop_TCI
eske_gift
suf_perf
suf_perf
Blood pressure (mmHg)
Blood pressure (mmHg)
Time (min)

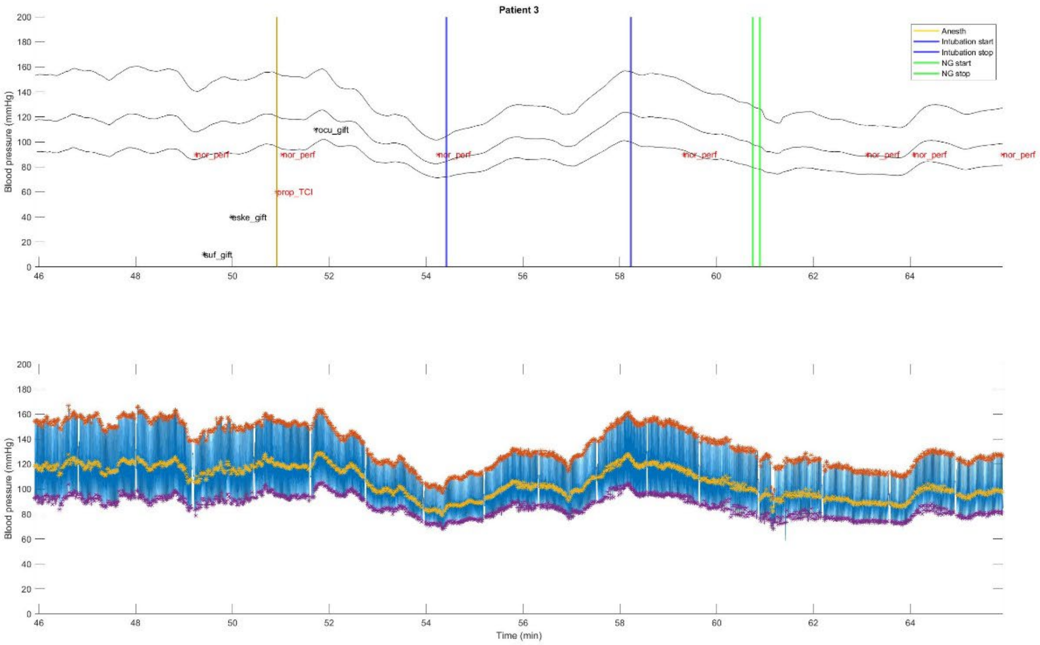
Patient 3
Anesth
Intubation start
Intubation stop
NG start
NG stop
Blood pressure (mmHg)
rocu_gift
nor_perf
prop_TCI
eske_gift
suf_gift
Time (min)

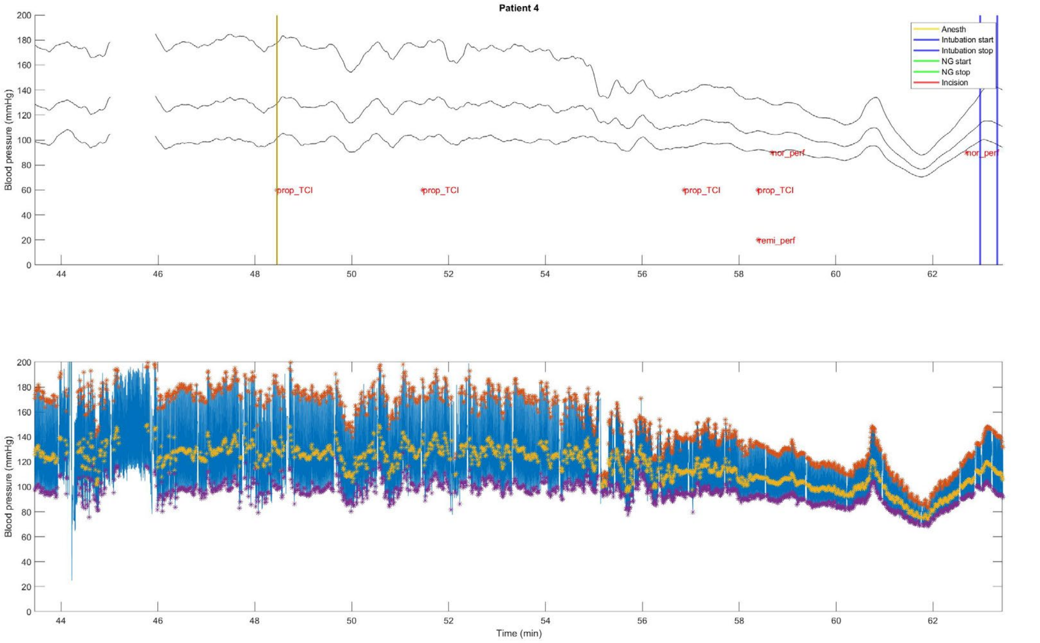
Patient 4
Anesth
Intubation start
Intubation stop
NG start
NG stop
Incision
Blood pressure (mmHg)
nor_perf
prop_TCI
remi_perf
Time (min)

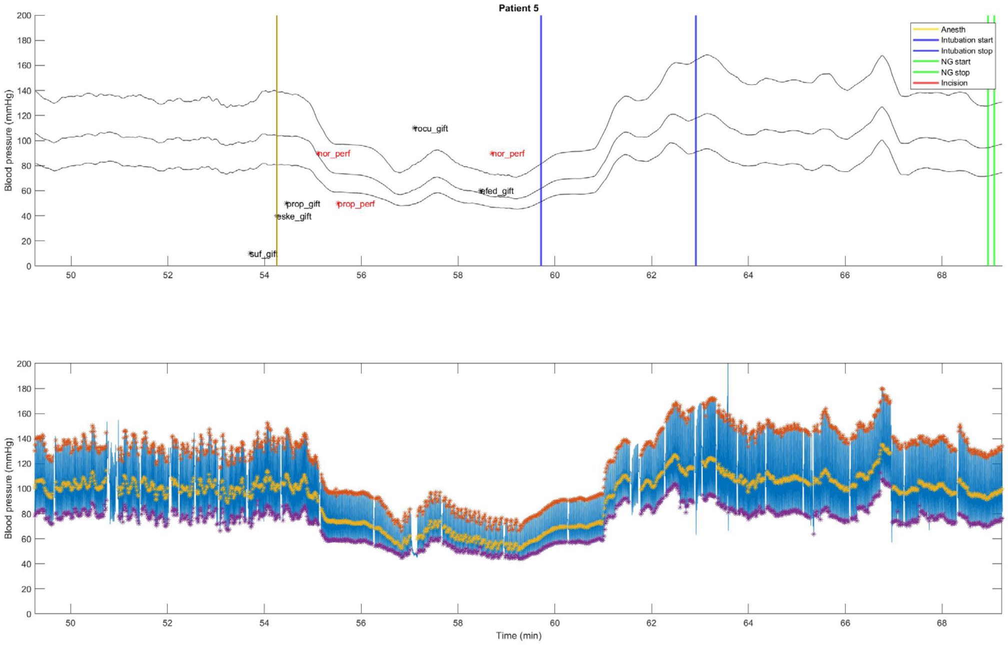

Patient 5
Blood pressure (mmHg)
Anesth
Intubation start
Intubation stop
NG start
NG stop
Incision
suf_gift
eske_gift
prop_gift
nor_perf
prop_perf
rocu_gift
nor_perf
efed_gift
Time (min)

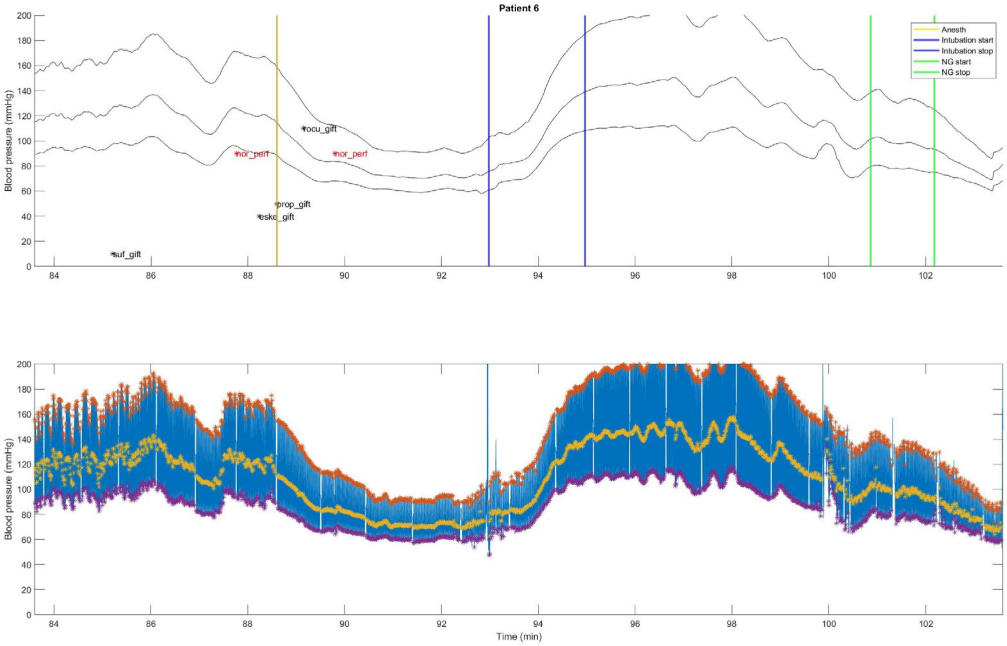

Patient 6
Blood pressure (mmHg)
Anesth
Intubation start
Intubation stop
NG start
NG stop
suf_gift
eske_gift
prop_gift
nor_perf
rocu_gift
nor_perf
Time (min)

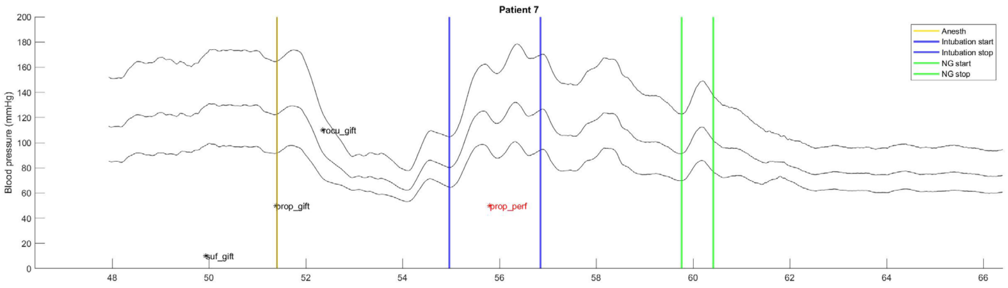
Patient 7
Blood pressure (mmHg)
Anesth
Intubation start
Intubation stop
NG start
NG stop
rocu_gift
prop_gift
prop_perf
suf_gift

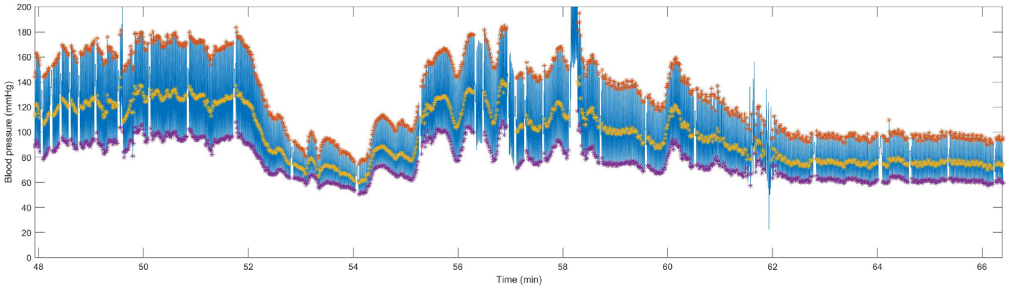
Blood pressure (mmHg)
Time (min)

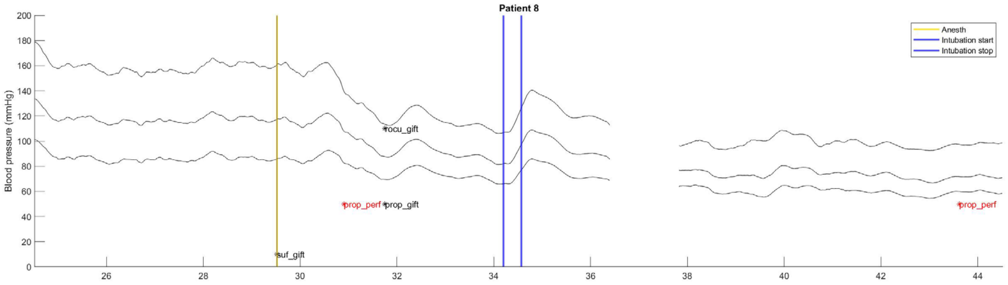
Patient 8
Blood pressure (mmHg)
Anesth
Intubation start
Intubation stop
rocu_gift
prop_perf
prop_gift
suf_gift
prop_perf

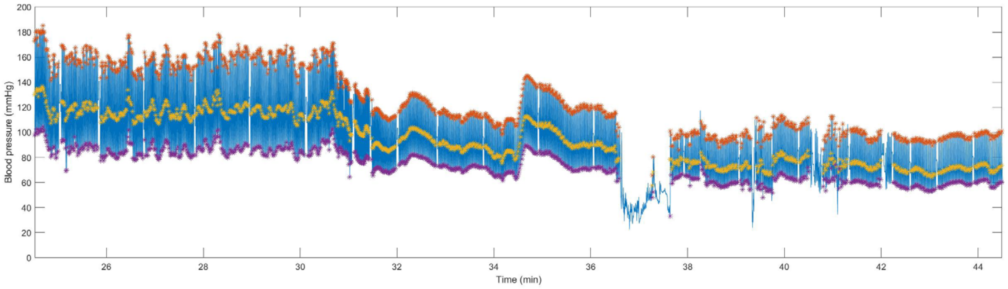
Blood pressure (mmHg)
Time (min)

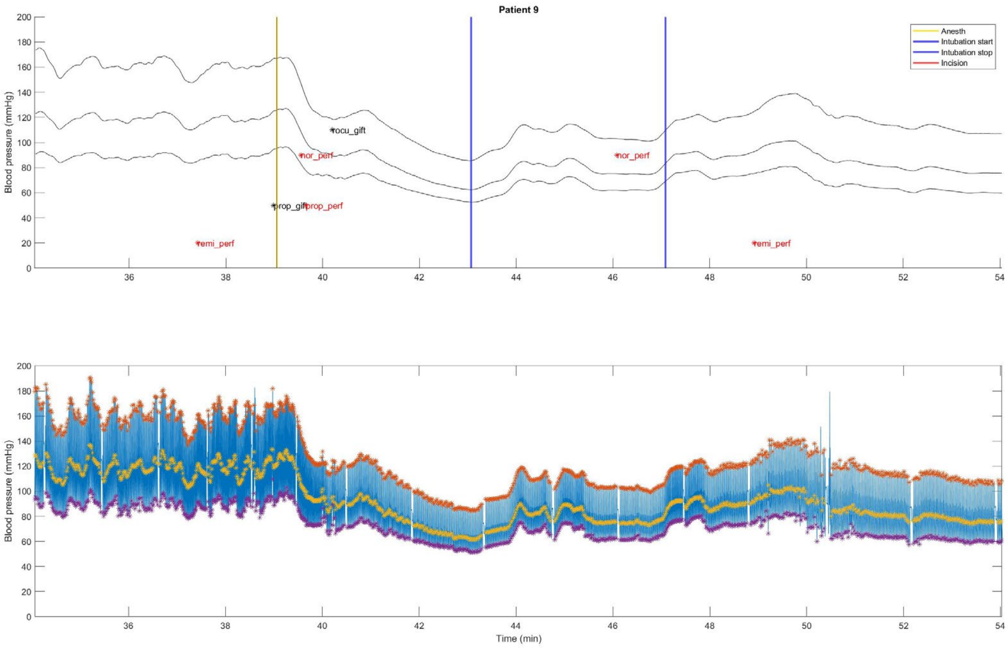
Patient 9
Anesth
Intubation start
Intubation stop
Incision
Blood pressure (mmHg)
rocu_gift
nor_perf
prop_gift
prop_perf
remi_perf
Time (min)

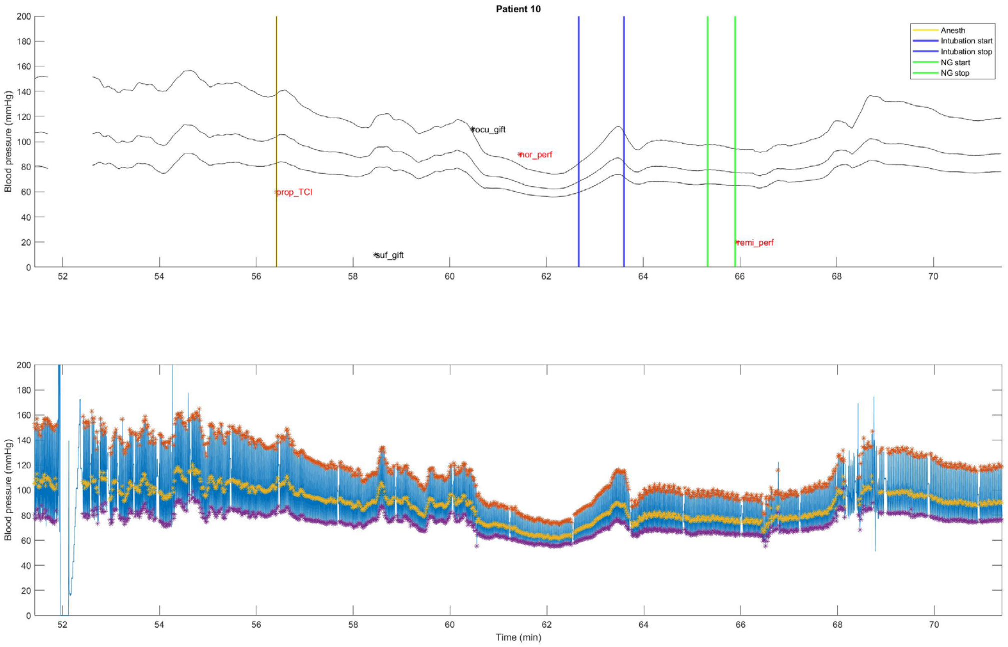
Patient 10
Anesth
Intubation start
Intubation stop
NG start
NG stop
Blood pressure (mmHg)
rocu_gift
nor_perf
prop_TCI
suf_gift
remi_perf
Time (min)

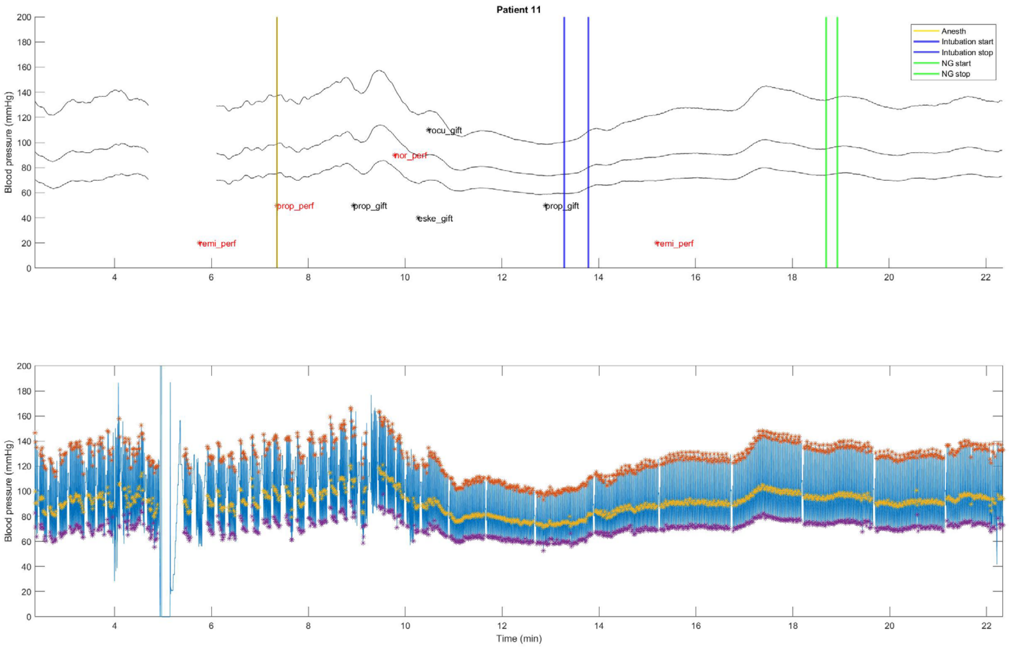
Patient 11
Anesth
Intubation start
Intubation stop
NG start
NG stop
rocu_gift
nor_perf
prop_perf
prop_gift
eske_gift
prop_gift
remi_perf
remi_perf
Blood pressure (mmHg)
Time (min)

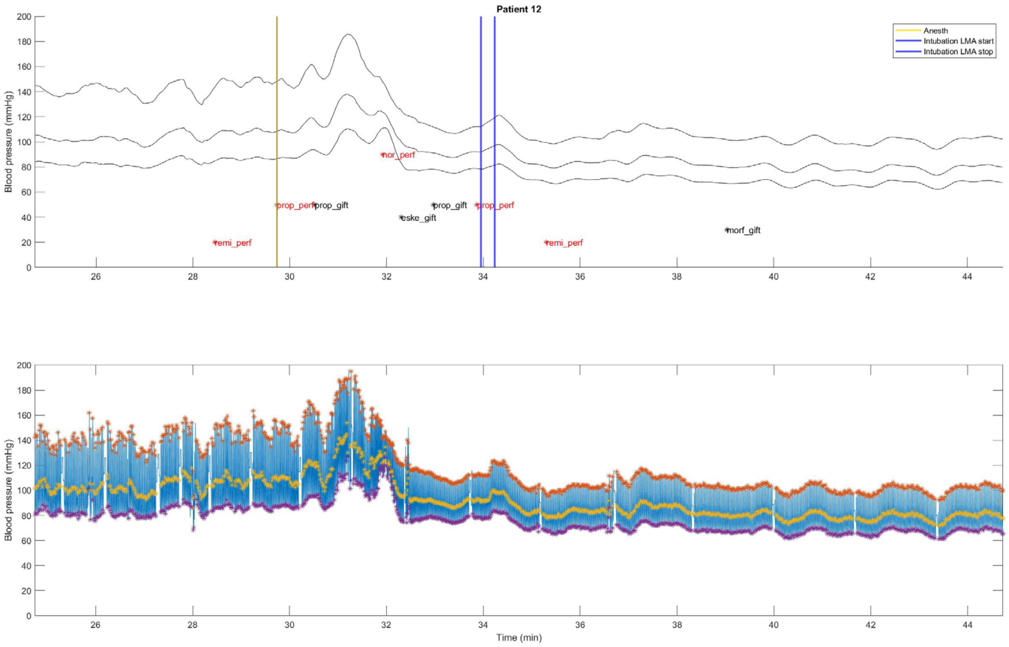
Patient 12
Anesth
Intubation LMA start
Intubation LMA stop
nor_perf
prop_perf
prop_gift
prop_gift
prop_perf
eske_gift
morf_gift
remi_perf
remi_perf
Blood pressure (mmHg)
Time (min)

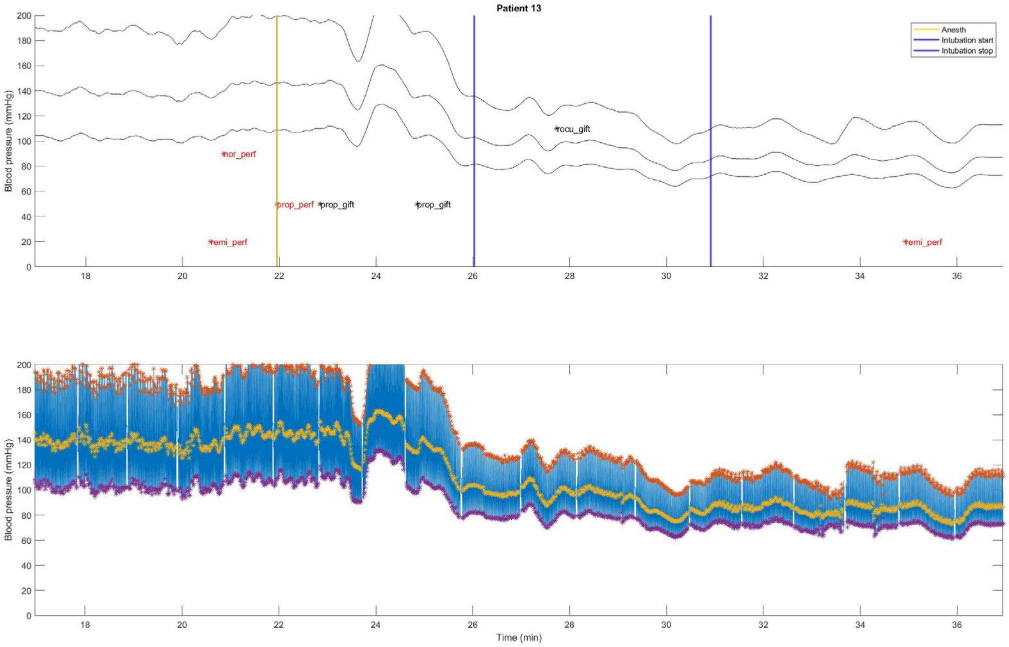

Patient 13
Anesth
Intubation start
Intubation stop
Blood pressure (mmHg)
nor_perf
prop_perf
prop_gift
prop_gift
rocu_gift
remi_perf
remi_perf
Time (min)

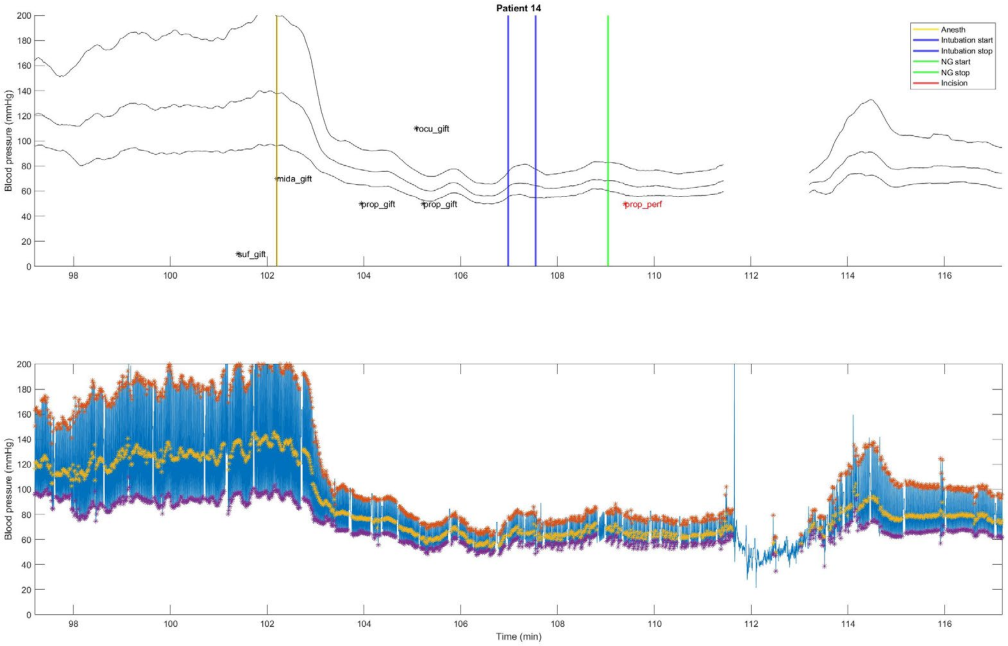

Patient 14
Anesth
Intubation start
Intubation stop
NG start
NG stop
Incision
Blood pressure (mmHg)
rocu_gift
mida_gift
prop_gift
prop_gift
prop_perf
suf_gift
Time (min)

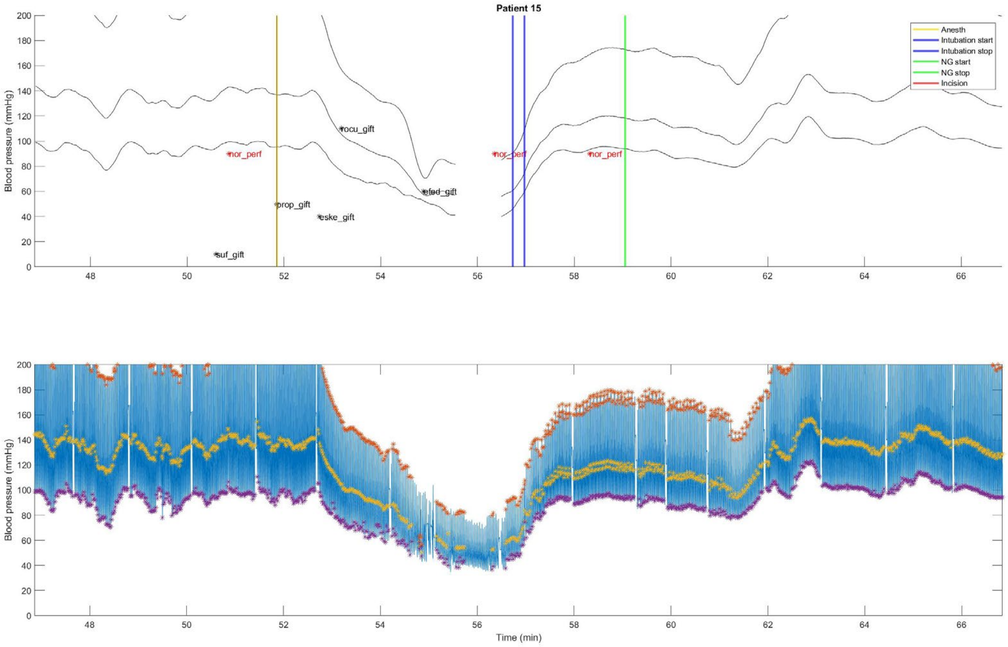
Patient 15
Anesth
Intubation start
Intubation stop
NG start
NG stop
Incision
Blood pressure (mmHg)
suf_gift
nor_perf
prop_gift
eske_gift
rocu_gift
efed_gift
Time (min)

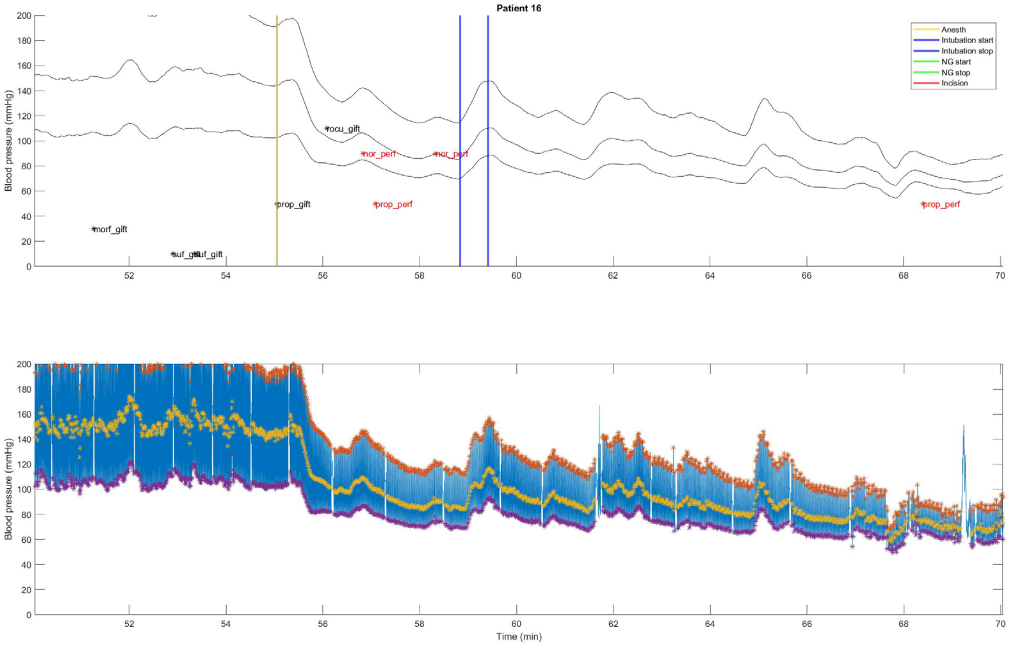
Patient 16
Anesth
Intubation start
Intubation stop
NG start
NG stop
Incision
Blood pressure (mmHg)
morf_gift
suf_gift
prop_gift
rocu_gift
nor_perf
prop_perf
Time (min)

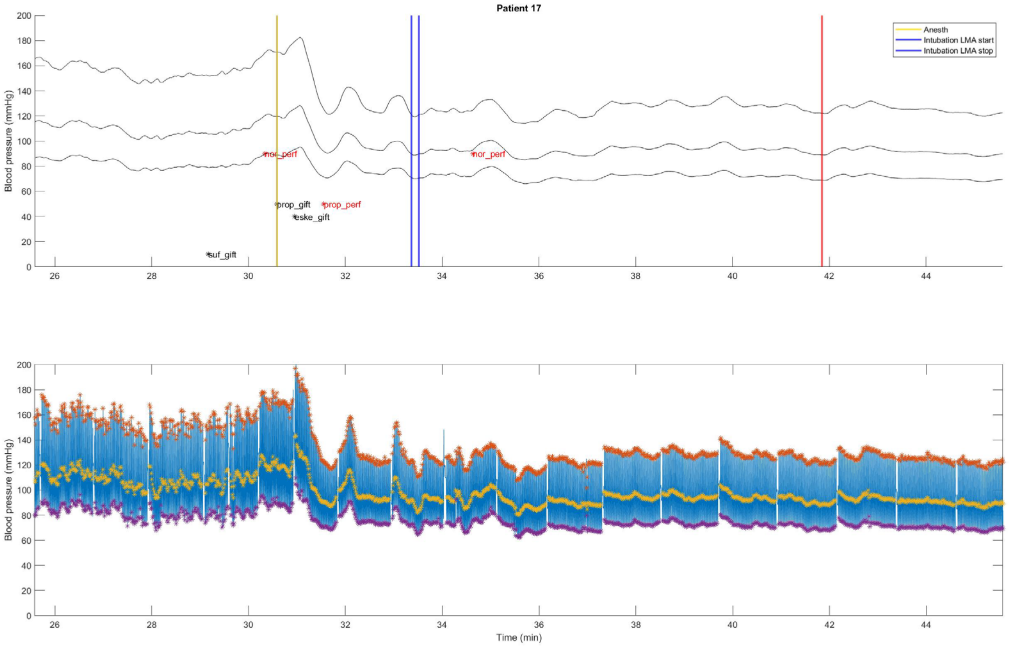
Patient 17
Anesth
Intubation LMA start
Intubation LMA stop
nor_perf
prop_gift
prop_perf
eske_gift
suf_gift
nor_perf
Blood pressure (mmHg)
Blood pressure (mmHg)
Time (min)

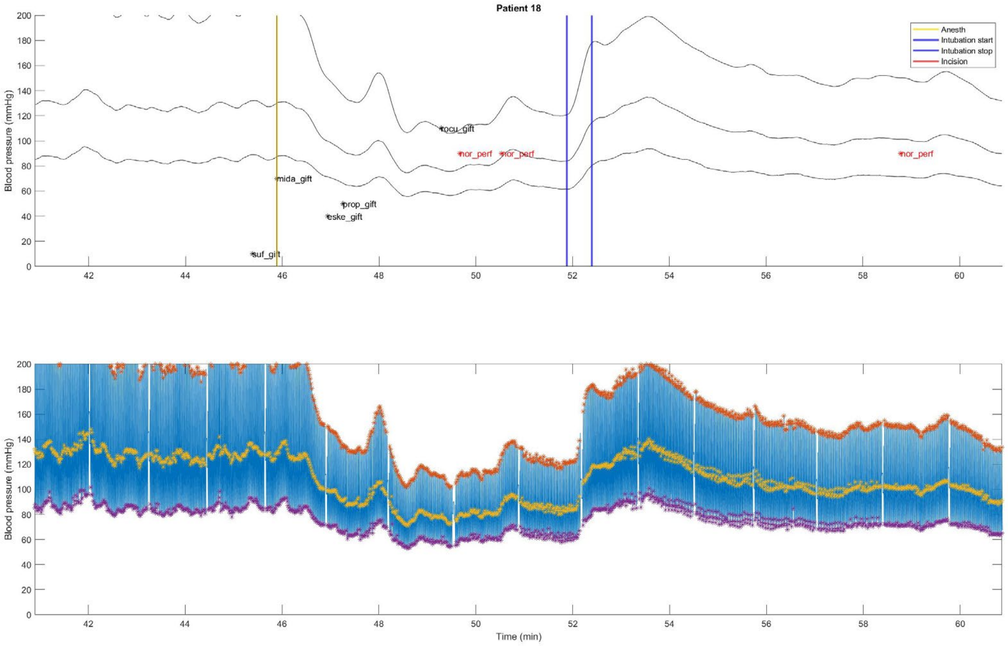
Patient 18
Anesth
Intubation start
Intubation stop
Incision
rocu_gift
nor_perf
nor_perf
nor_perf
mida_gift
prop_gift
eske_gift
suf_gift
Blood pressure (mmHg)
Blood pressure (mmHg)
Time (min)

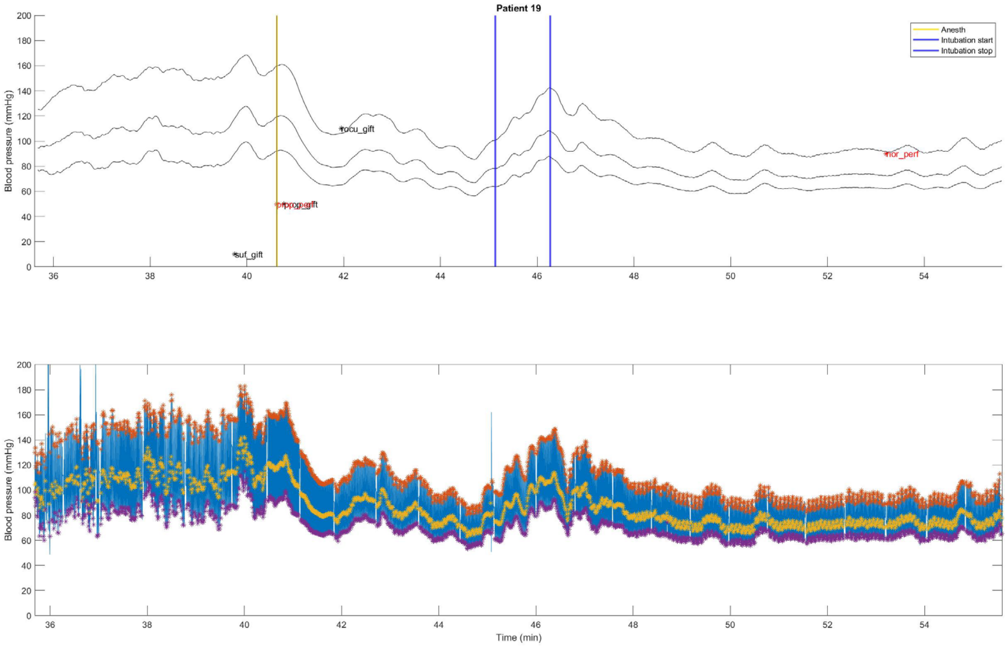
Patient 19
Anesth
Intubation start
Intubation stop
Blood pressure (mmHg)
rocu_gift
prop_gift
suf_gift
nor_perf
Time (min)

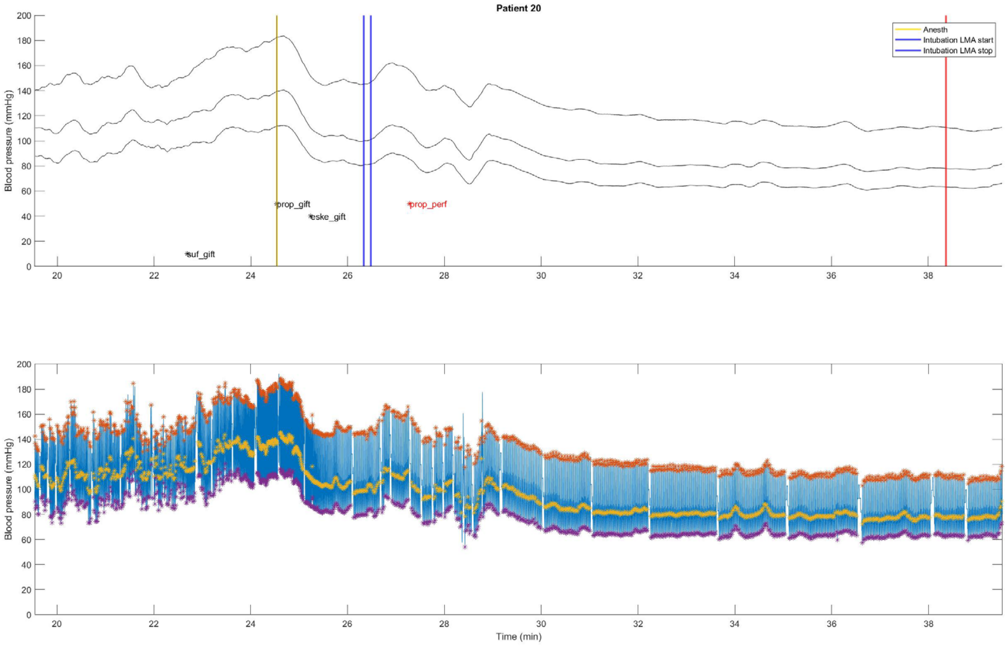
Patient 20
Anesth
Intubation LMA start
Intubation LMA stop
Blood pressure (mmHg)
prop_gift
eske_gift
prop_perf
suf_gift
Time (min)

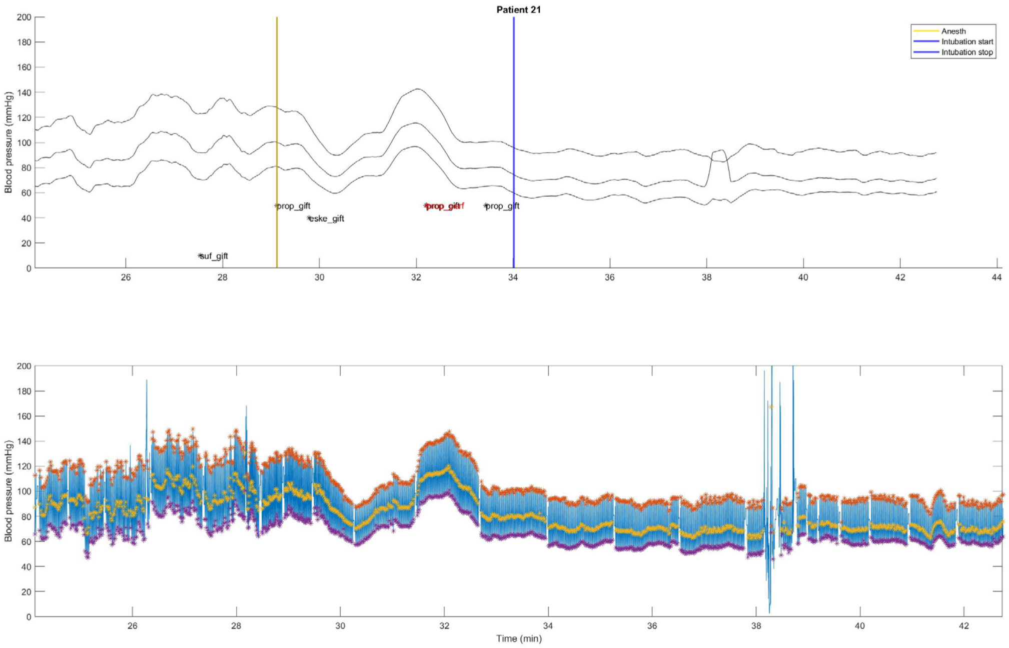
Patient 21
Blood pressure (mmHg)
Anesth
Intubation start
Intubation stop
prop_gift
eske_gift
suf_gift
Time (min)

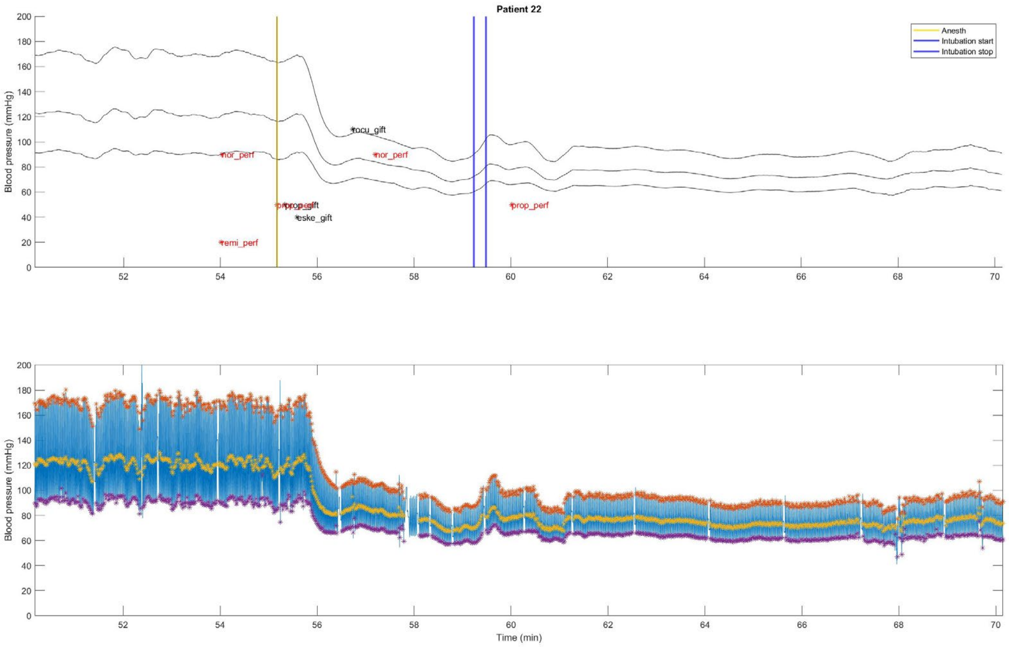
Patient 22
Blood pressure (mmHg)
Anesth
Intubation start
Intubation stop
rocu_gift
nor_perf
prop_gift
eske_gift
prop_perf
remi_perf
Time (min)

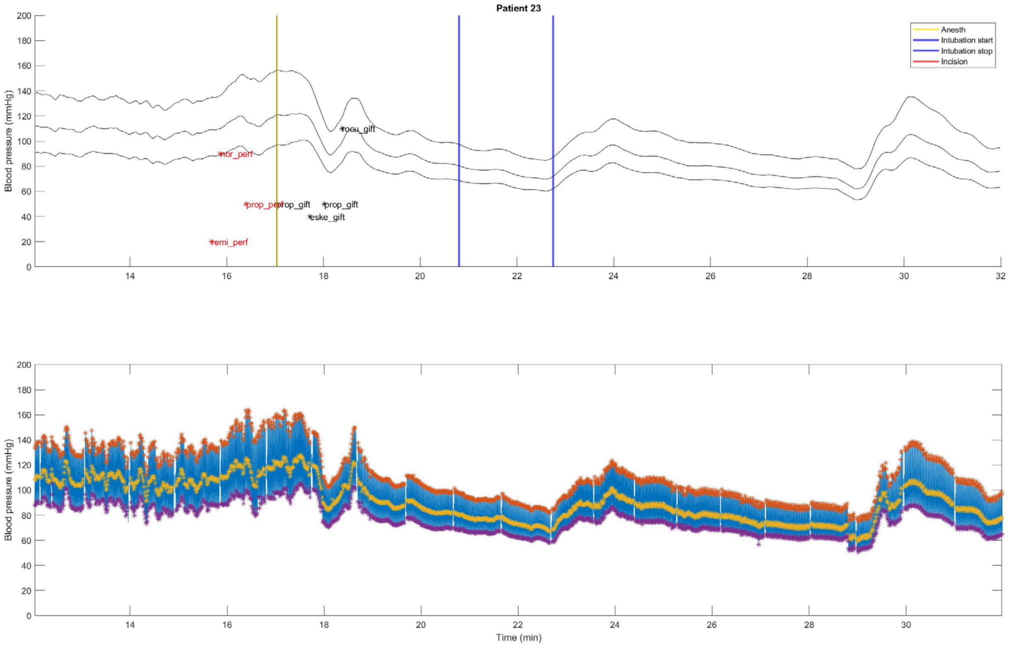

Patient 23
Anesth
Intubation start
Intubation stop
Incision
Blood pressure (mmHg)
Time (min)
nor_perf
prop_perf
prop_gift
prop_gift
eske_gift
rocu_gift
remi_perf

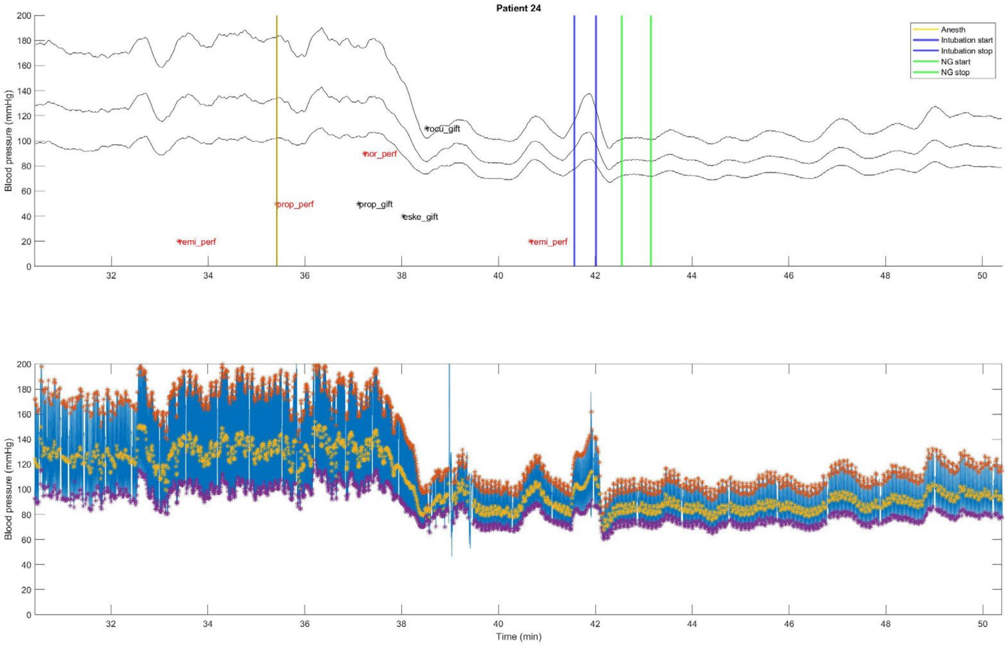

Patient 24
Anesth
Intubation start
Intubation stop
NG start
NG stop
Blood pressure (mmHg)
Time (min)
rocu_gift
nor_perf
prop_perf
prop_gift
eske_gift
remi_perf
remi_perf

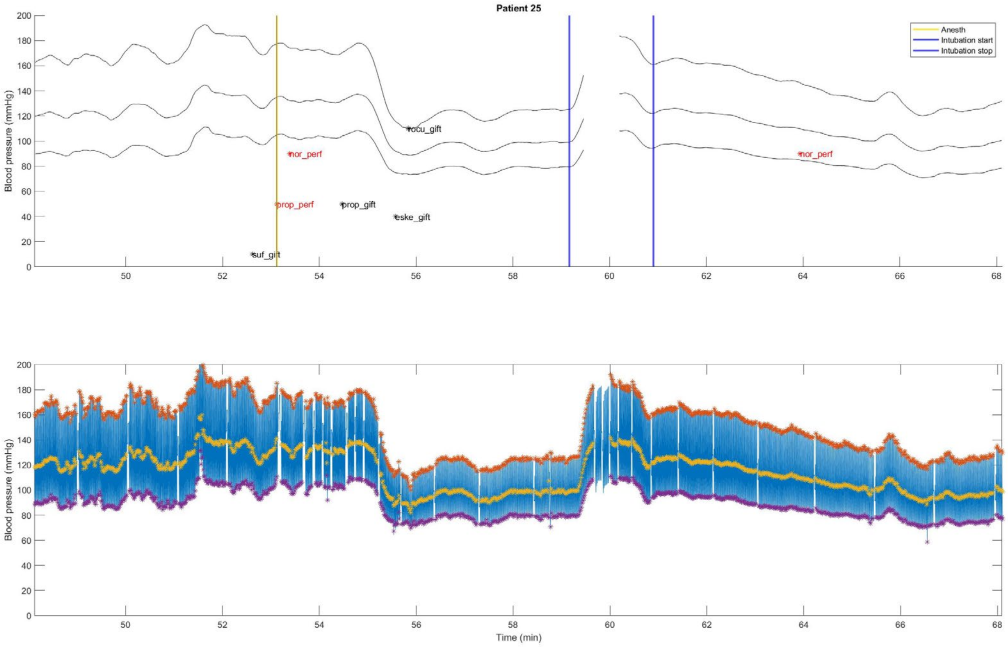

Patient 25
Anesth
Intubation start
Intubation stop
Blood pressure (mmHg)
suf_gift
prop_perf
nor_perf
prop_gift
eske_gift
rocu_gift
nor_perf
Blood pressure (mmHg)
Time (min)

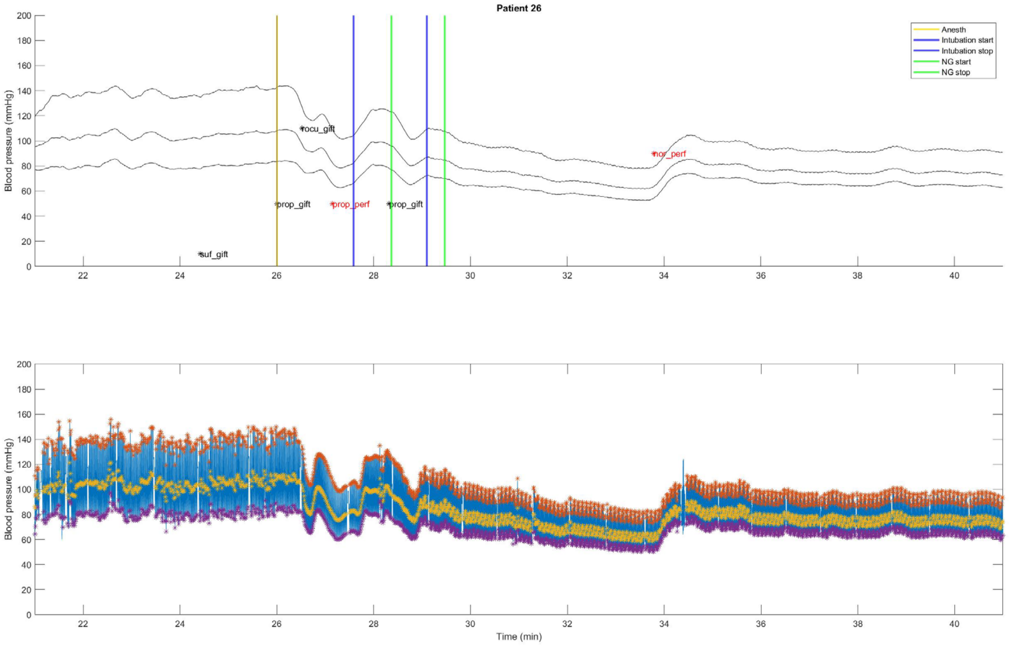

Patient 26
Anesth
Intubation start
Intubation stop
NG start
NG stop
Blood pressure (mmHg)
rocu_gift
nor_perf
prop_gift
prop_perf
prop_gift
suf_gift
Blood pressure (mmHg)
Time (min)

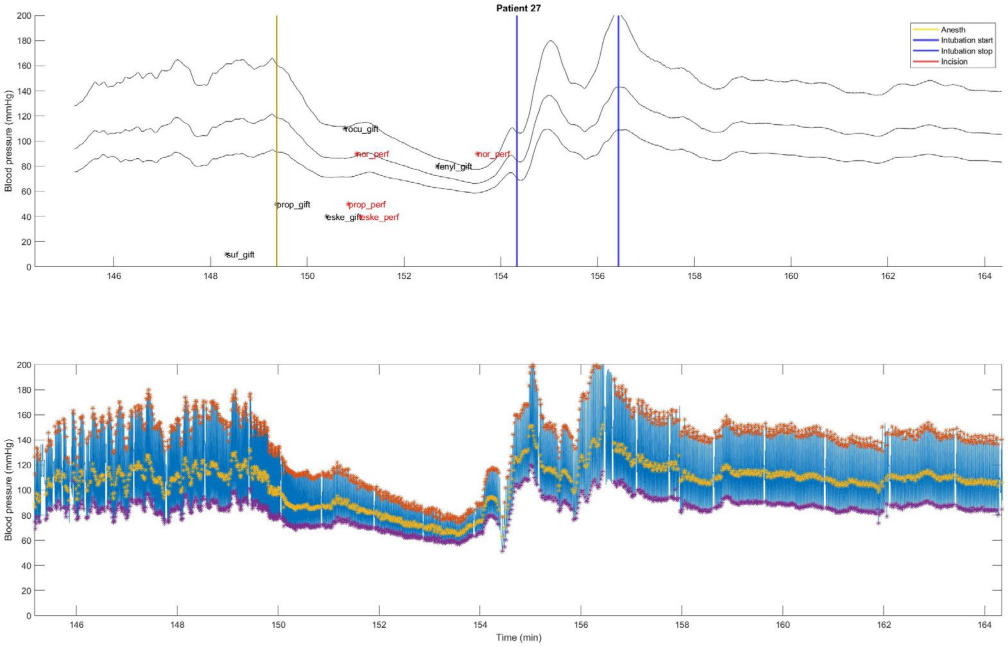

Patient 27
Anesth
Intubation start
Intubation stop
Incision
Blood pressure (mmHg)
rocu_gift
nor_perf
fenyl_gift
nor_perf
prop_gift
prop_perf
eske_gift
eske_perf
suf_gift
Time (min)

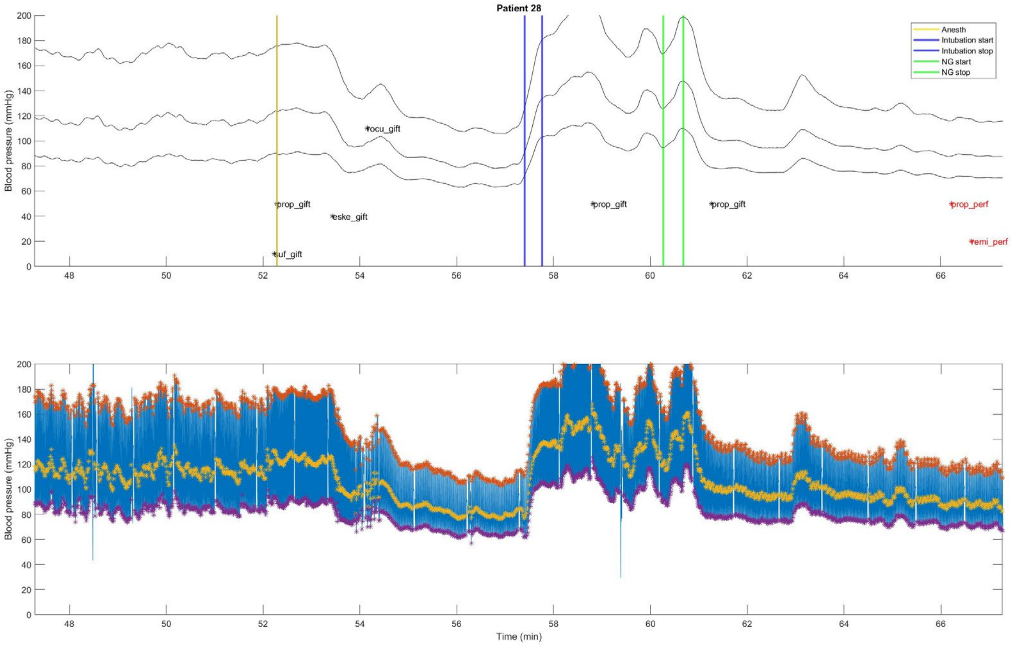

Patient 28
Anesth
Intubation start
Intubation stop
NG start
NG stop
Blood pressure (mmHg)
rocu_gift
prop_gift
eske_gift
prop_gift
prop_gift
prop_perf
suf_gift
remi_perf
Time (min)

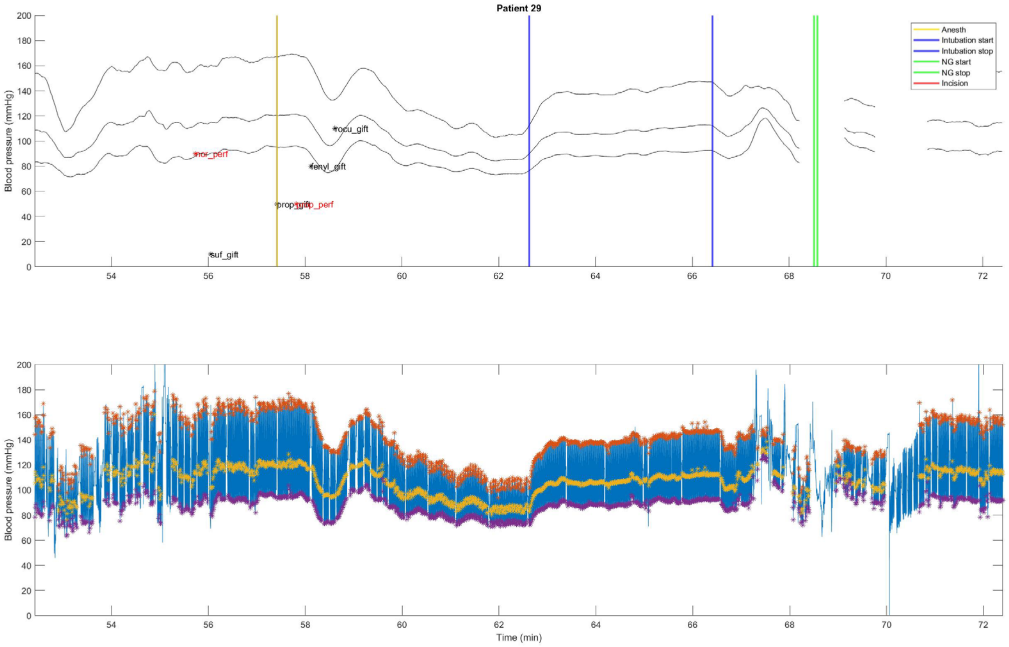
Patient 29
Anesth
Intubation start
Intubation stop
NG start
NG stop
Incision
Blood pressure (mmHg)
nor_perf
prop_gift
prop_perf
suf_gift
rocu_gift
fenyl_gift
Time (min)

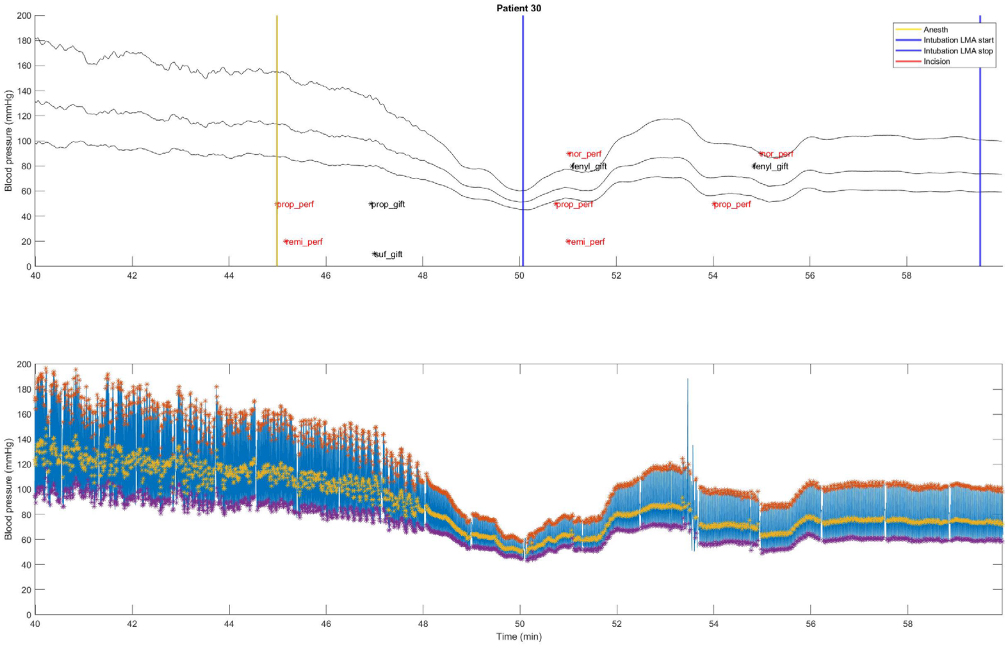
Patient 30
Anesth
Intubation LMA start
Intubation LMA stop
Incision
Blood pressure (mmHg)
prop_perf
remi_perf
prop_gift
suf_gift
nor_perf
fenyl_gift
Time (min)

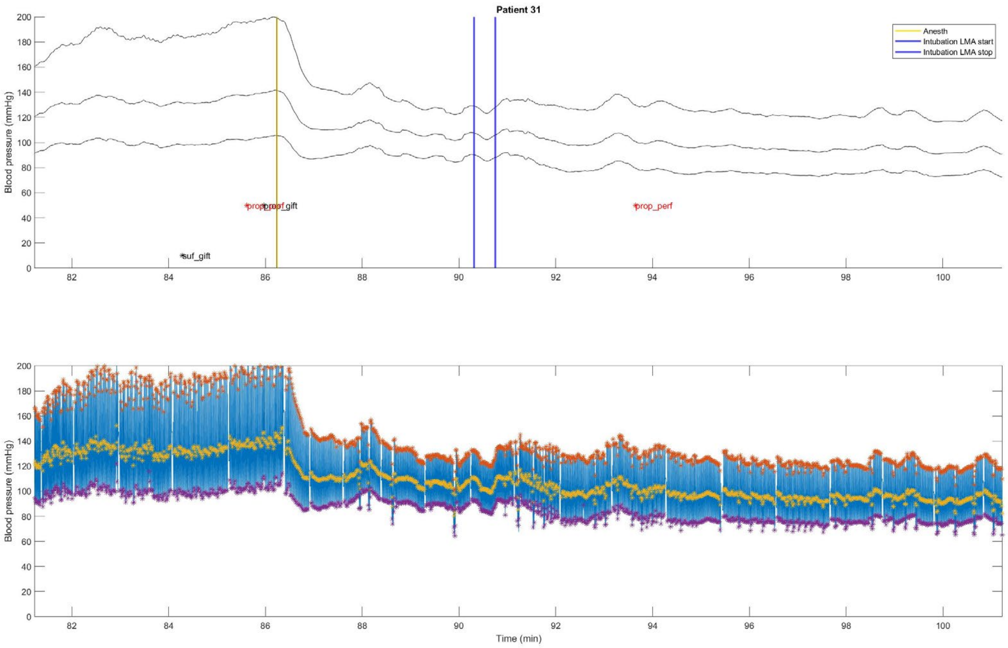
Patient 31
Anesth
Intubation LMA start
Intubation LMA stop
Blood pressure (mmHg)
suf_gift
prop_perf
Time (min)

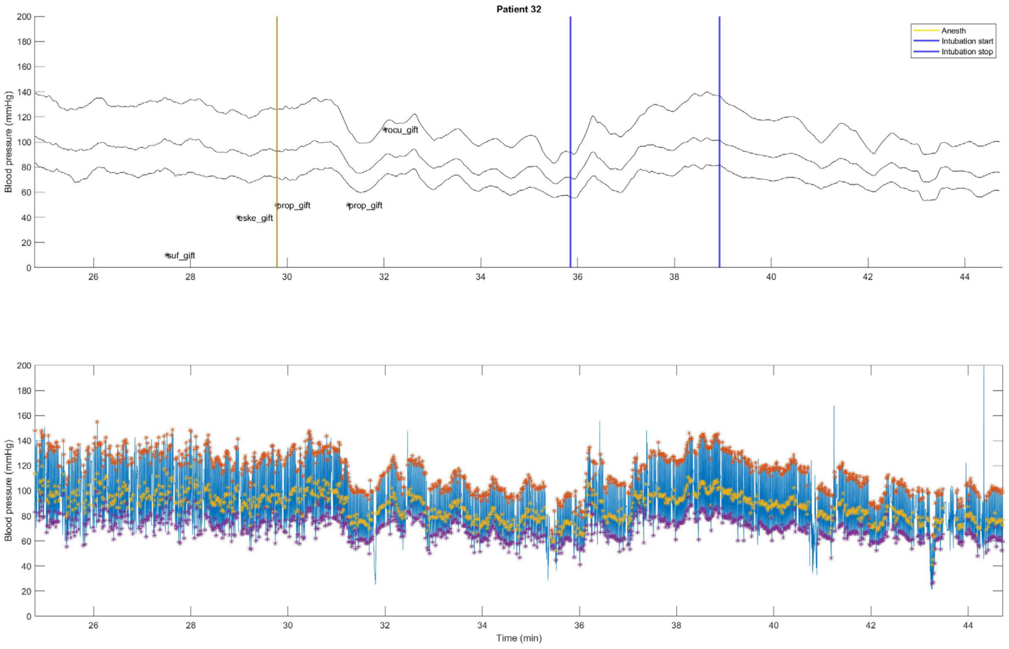
Patient 32
Anesth
Intubation start
Intubation stop
Blood pressure (mmHg)
rocu_gift
prop_gift
eske_gift
suf_gift
Time (min)

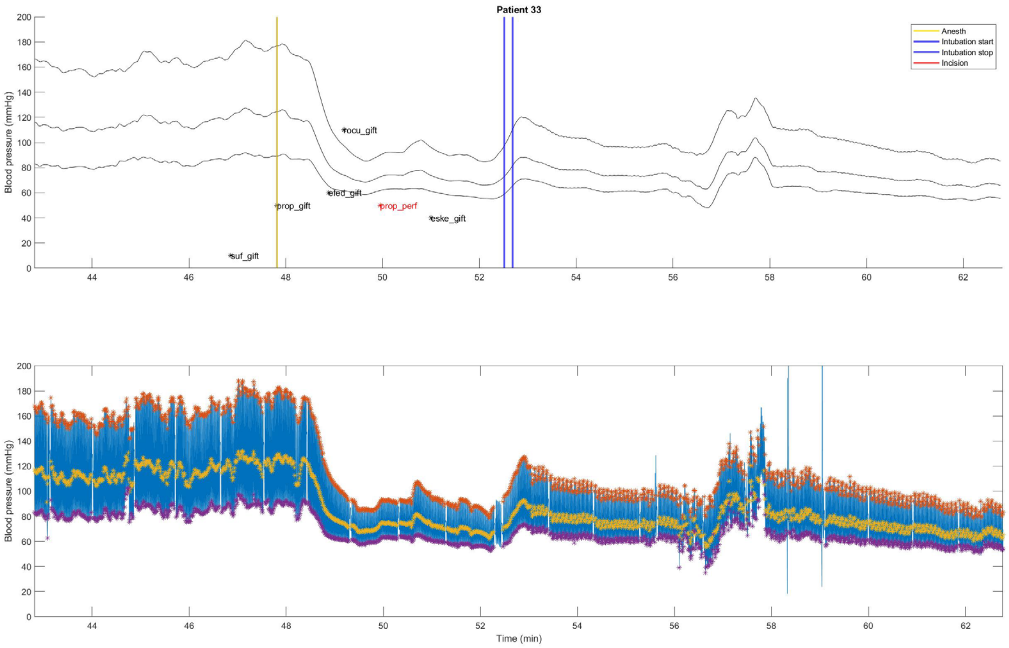
Patient 33
Anesth
Intubation start
Intubation stop
Incision
Blood pressure (mmHg)
ocu_gift
prop_gift
oled_gift
prop_perf
eske_gift
suf_gift
Time (min)

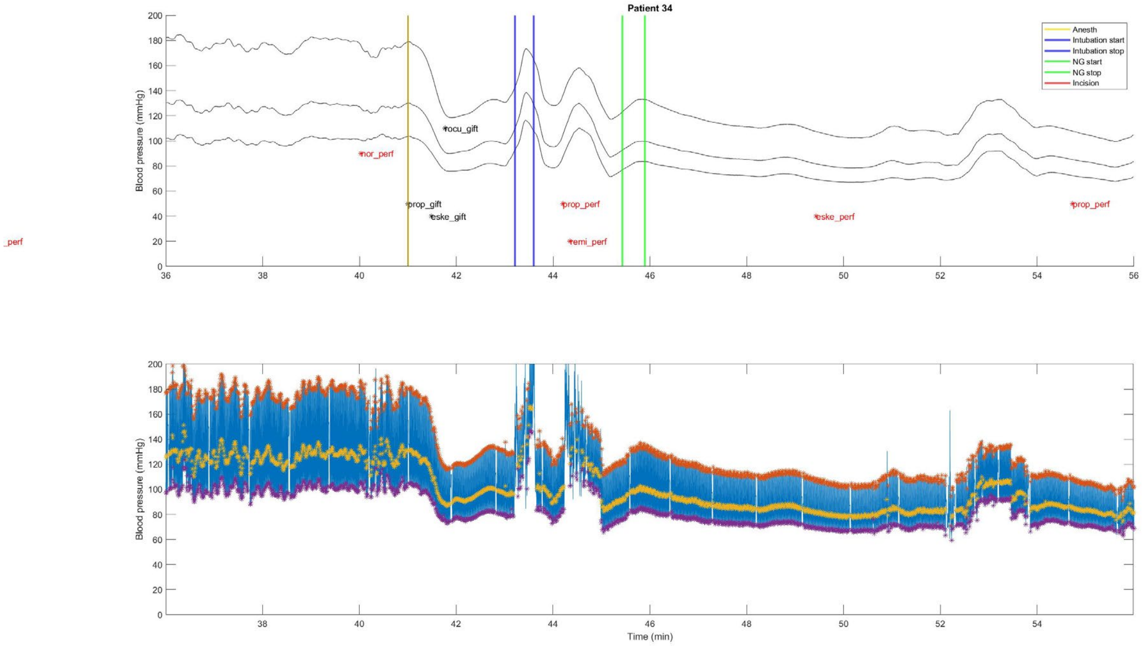
Patient 34
Anesth
Intubation start
Intubation stop
NG start
NG stop
Incision
Blood pressure (mmHg)
nor_perf
rocu_gift
prop_gift
eske_gift
prop_perf
remi_perf
eske_perf
prop_perf
_perf
Time (min)

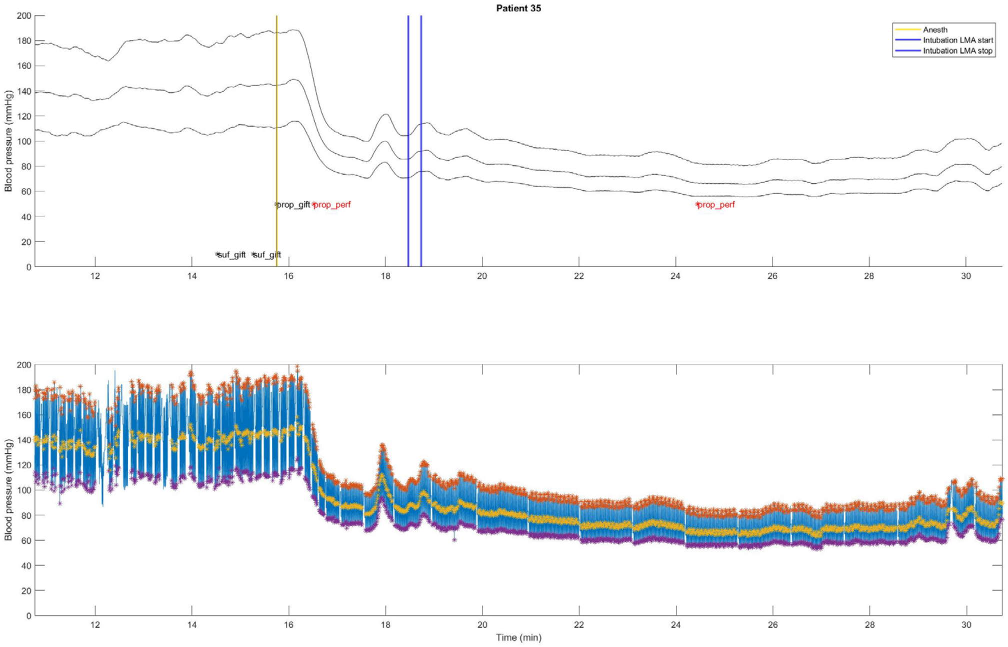
Patient 35
Anesth
Intubation LMA start
Intubation LMA stop
Blood pressure (mmHg)
prop_gift
prop_perf
prop_perf
suf_gift
suf_gift
Time (min)

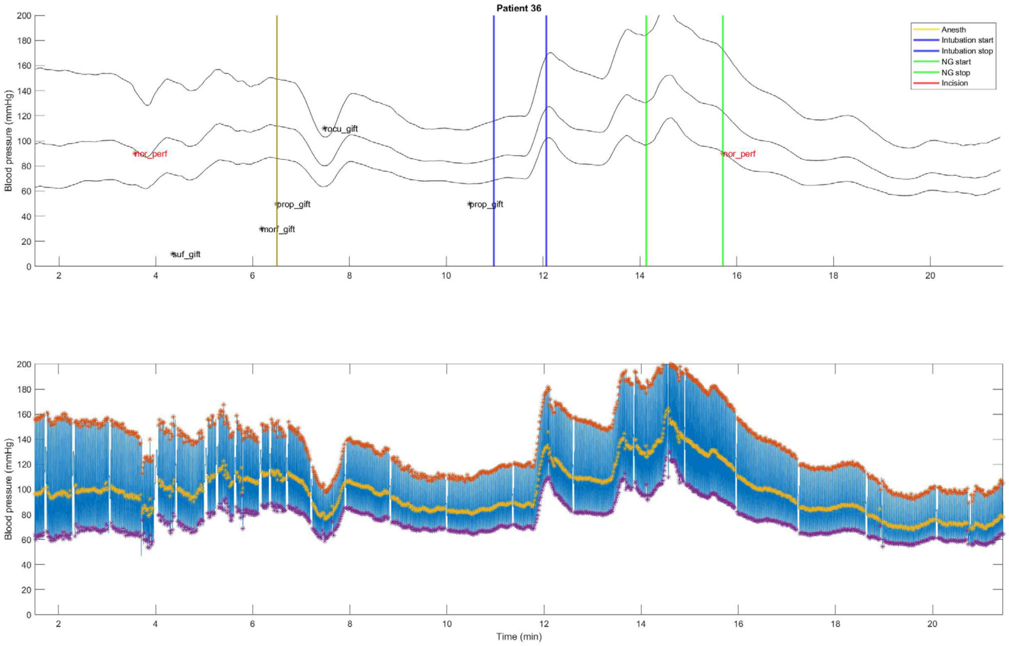
Patient 36
Anesth
Intubation start
Intubation stop
NG start
NG stop
Incision
Blood pressure (mmHg)
rocu_gift
nor_perf
nor_perf
prop_gift
prop_gift
nor_gift
suf_gift
Time (min)

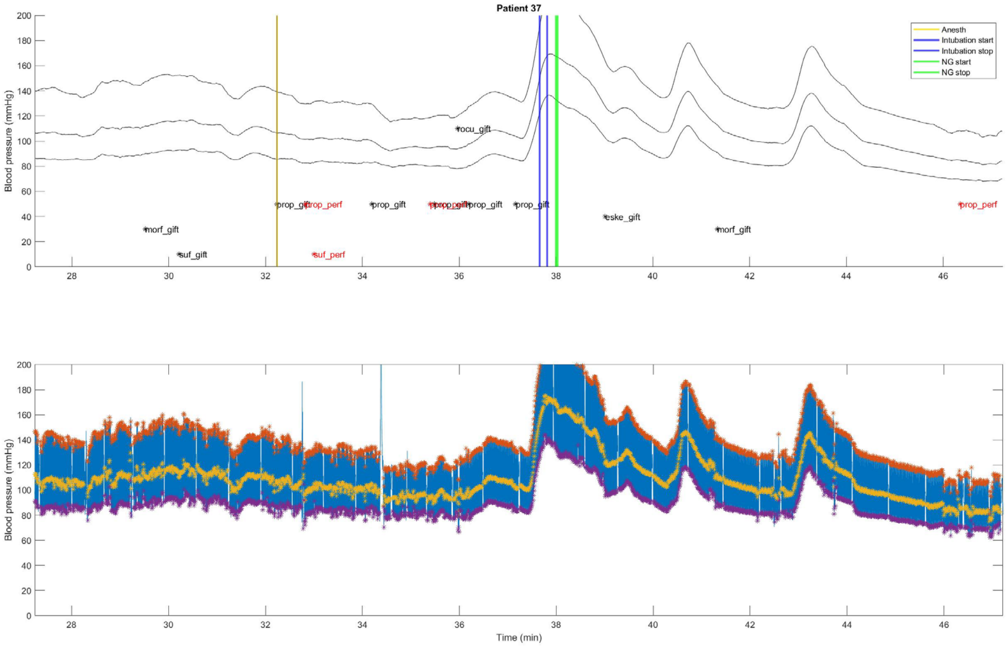
Patient 37
Anesth
Intubation start
Intubation stop
NG start
NG stop
Blood pressure (mmHg)
morf_gift
suf_gift
prop_gift
prop_perf
suf_perf
rocu_gift
eske_gift
Time (min)

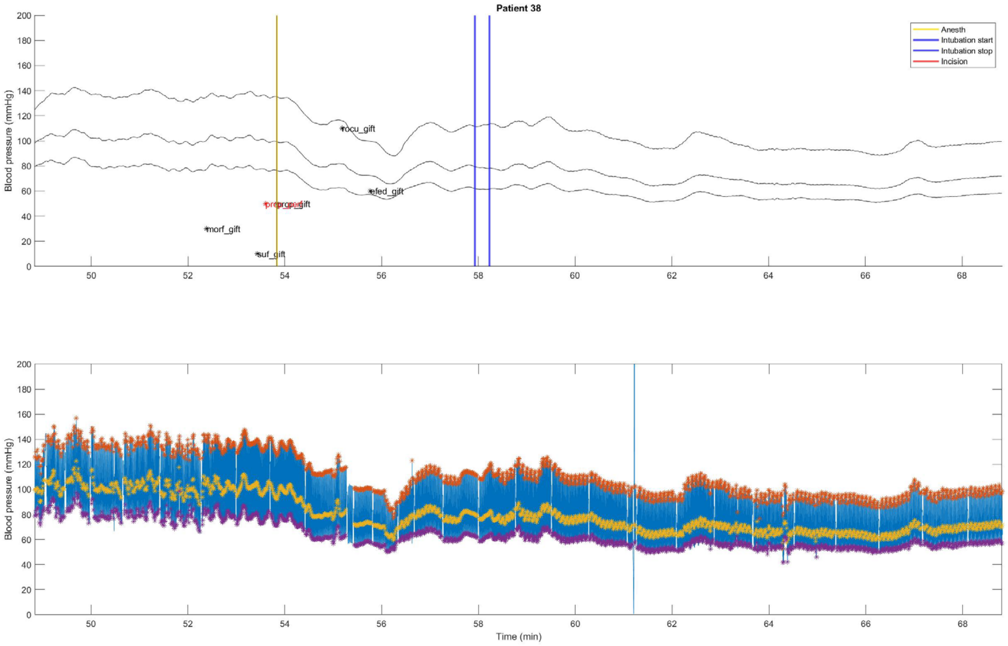
Patient 38
Anesth
Intubation start
Intubation stop
Incision
Blood pressure (mmHg)
rocu_gift
efed_gift
morf_gift
suf_gift
Time (min)

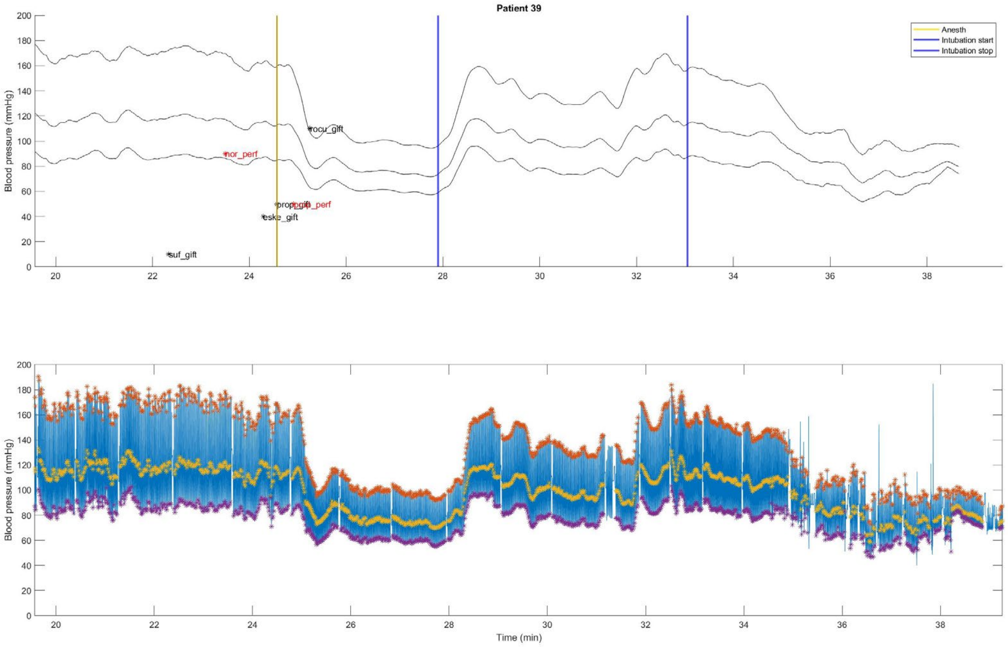
Patient 39
Anesth
Intubation start
Intubation stop
Blood pressure (mmHg)
ocu_gift
nor_perf
suf_gift
Time (min)

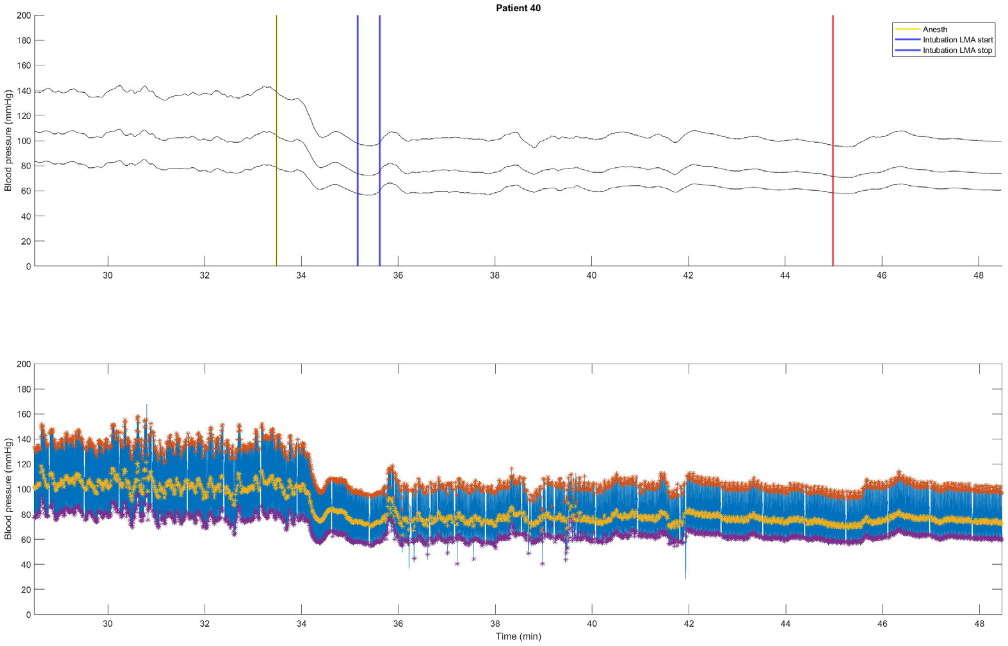
Patient 40
Anesth
Intubation LMA start
Intubation LMA stop
Blood pressure (mmHg)
Time (min)

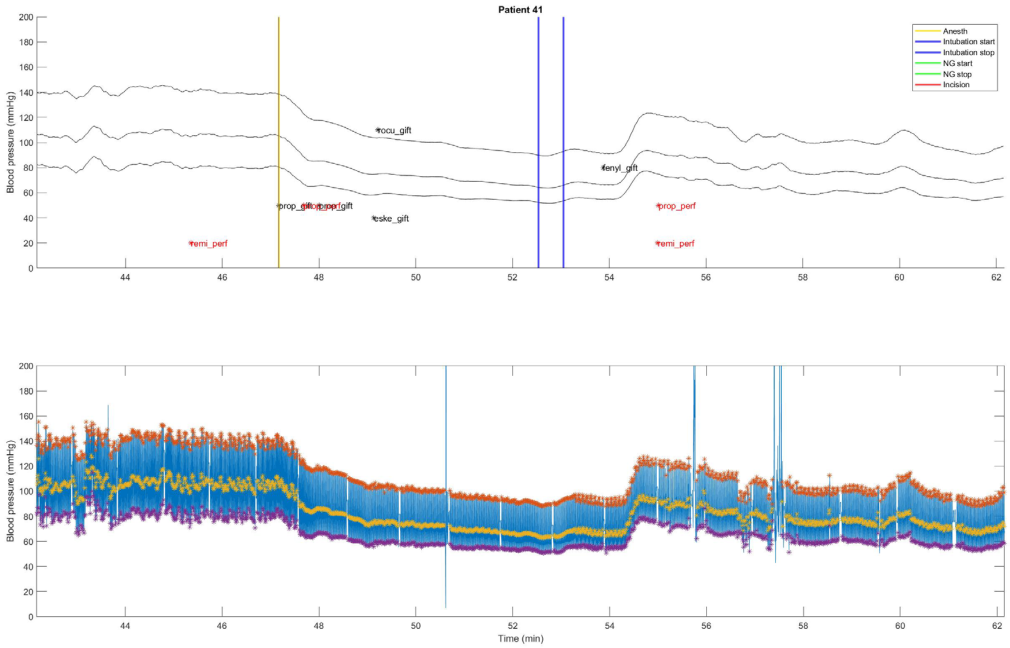
Patient 41
Anesth
Intubation start
Intubation stop
NG start
NG stop
Incision
rocu_gift
fenyl_gift
prop_gift
eske_gift
remi_perf
prop_perf
Blood pressure (mmHg)
Time (min)

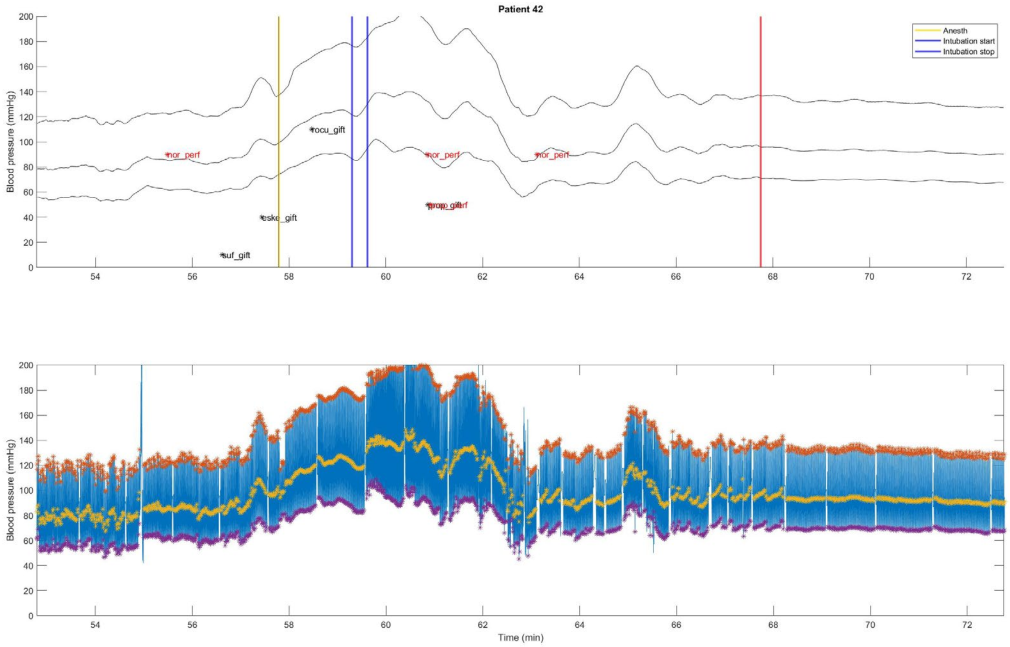
Patient 42
Anesth
Intubation start
Intubation stop
rocu_gift
nor_perf
eske_gift
suf_gift
Blood pressure (mmHg)
Time (min)

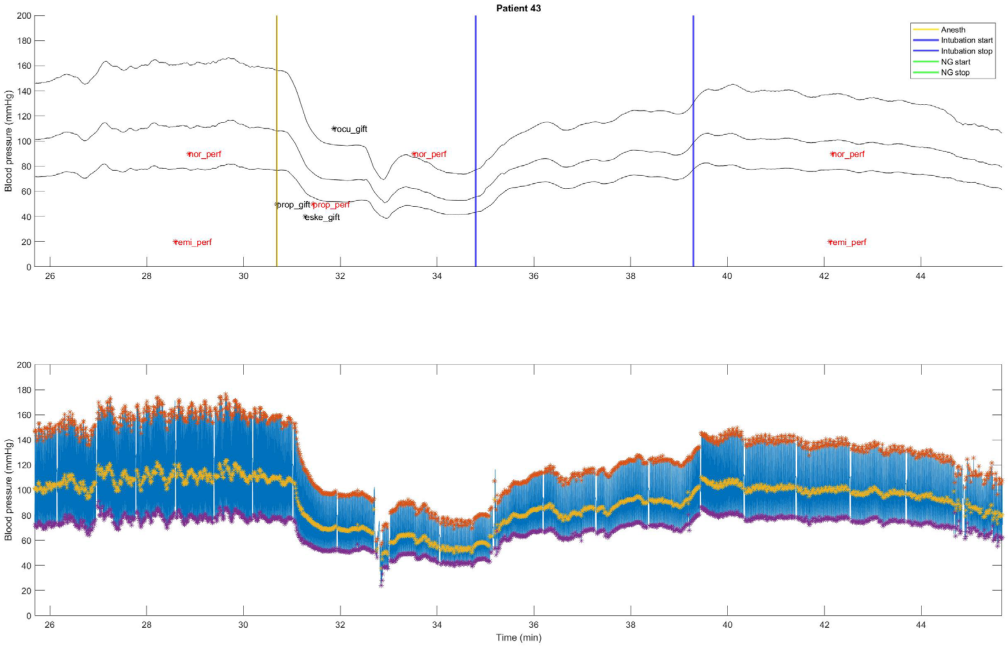
Patient 43
Blood pressure (mmHg)
Time (min)
Anesth
Intubation start
Intubation stop
NG start
NG stop
rocu_gift
nor_perf
prop_gift
prop_perf
eske_gift
remi_perf

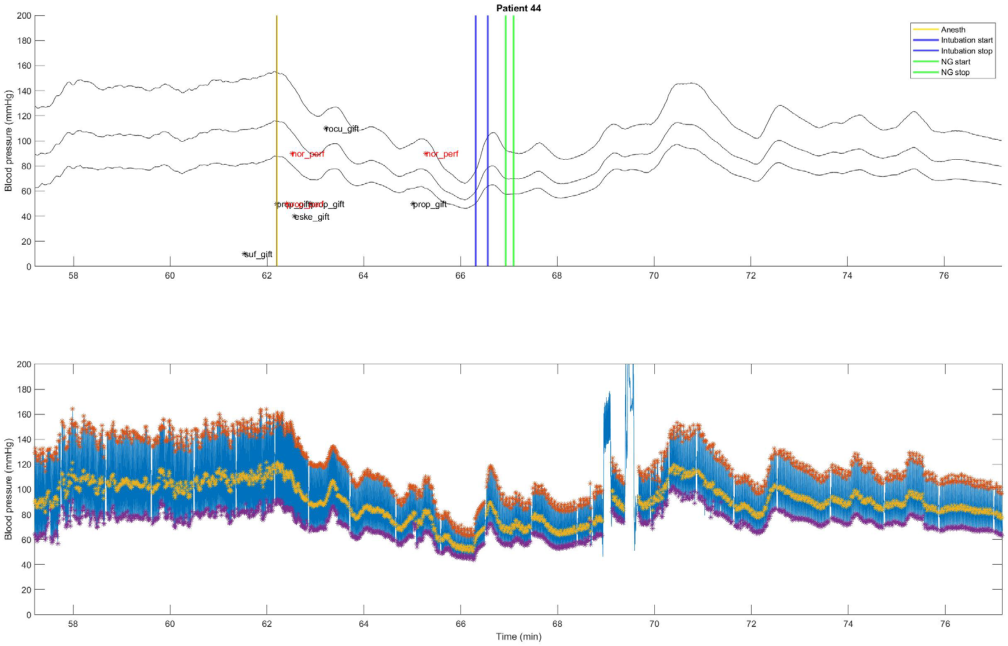
Patient 44
Blood pressure (mmHg)
Time (min)
Anesth
Intubation start
Intubation stop
NG start
NG stop
rocu_gift
nor_perf
prop_gift
eske_gift
suf_gift

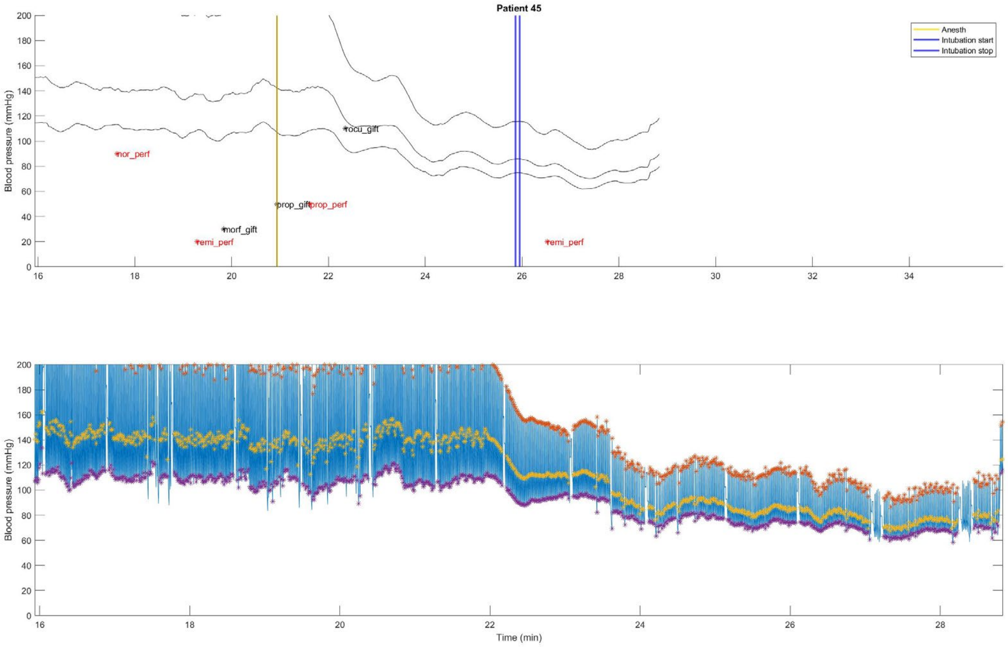

Patient 45
Anesth
Intubation start
Intubation stop
Blood pressure (mmHg)
nor_perf
remi_perf
morf_gift
prop_gift
prop_perf
rocu_gift
remi_perf
Blood pressure (mmHg)
Time (min)

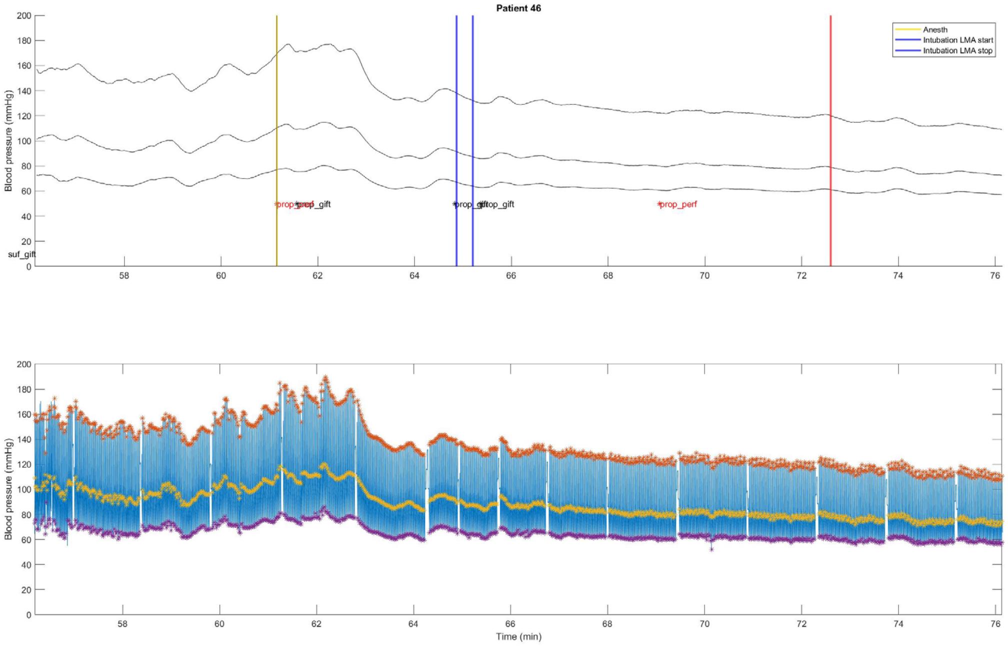

Patient 46
Anesth
Intubation LMA start
Intubation LMA stop
Blood pressure (mmHg)
suf_gift
prop_perf
prop_gift
rocu_gift
Blood pressure (mmHg)
Time (min)

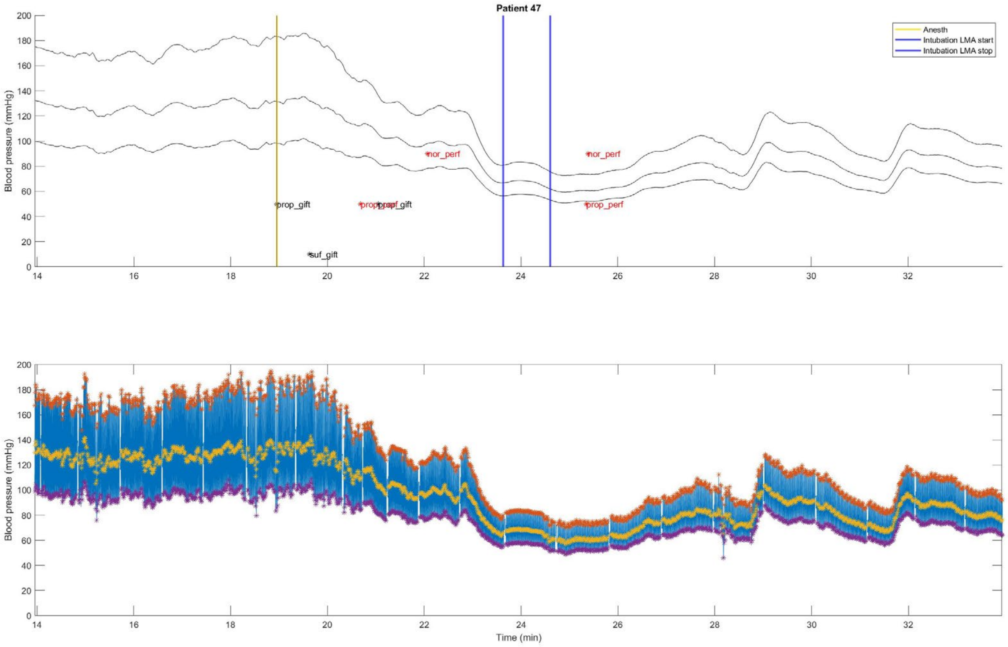
Patient 47
Anesth
Intubation LMA start
Intubation LMA stop
Blood pressure (mmHg)
prop_gift
suf_gift
nor_perf
prop_perf
Time (min)

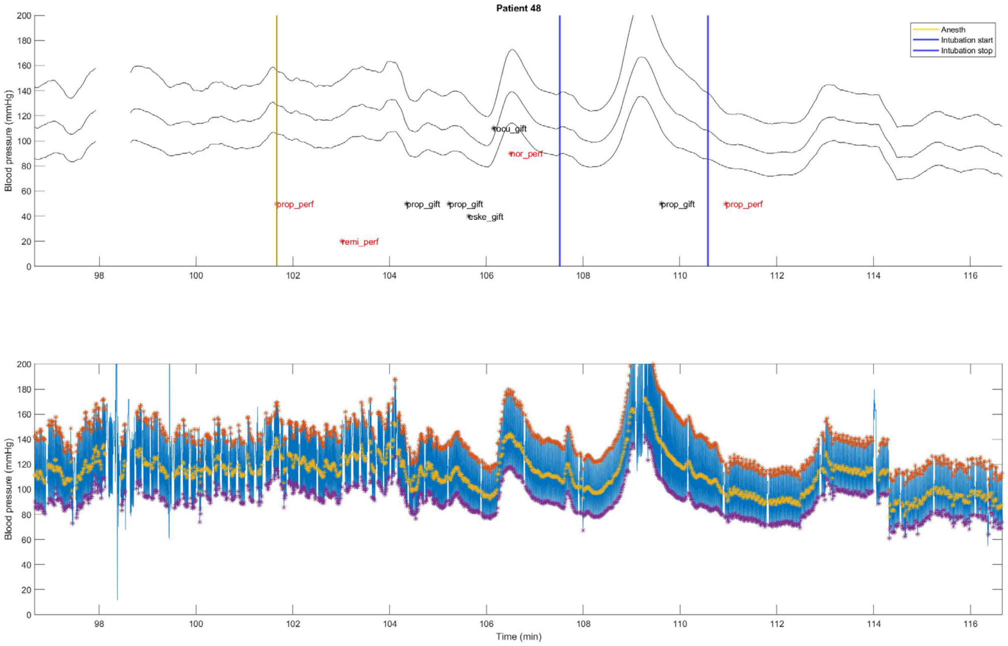
Patient 48
Anesth
Intubation start
Intubation stop
Blood pressure (mmHg)
prop_perf
remi_perf
prop_gift
eske_gift
rocu_gift
nor_perf
Time (min)

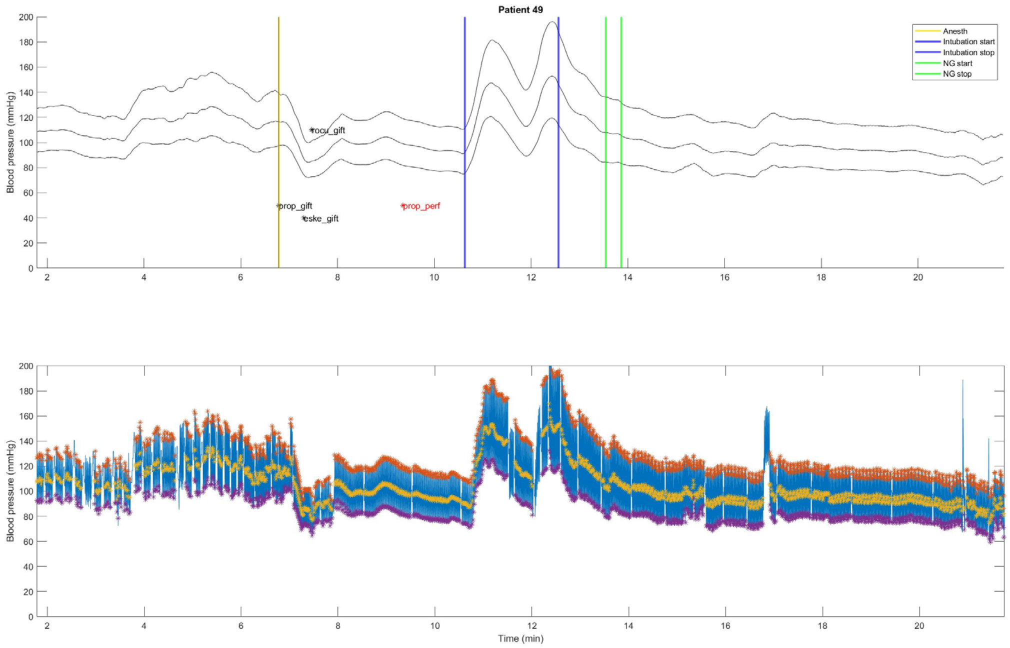
Patient 49
Anesth
Intubation start
Intubation stop
NG start
NG stop
rocu_gift
prop_gift
eske_gift
prop_perf
Blood pressure (mmHg)
Time (min)

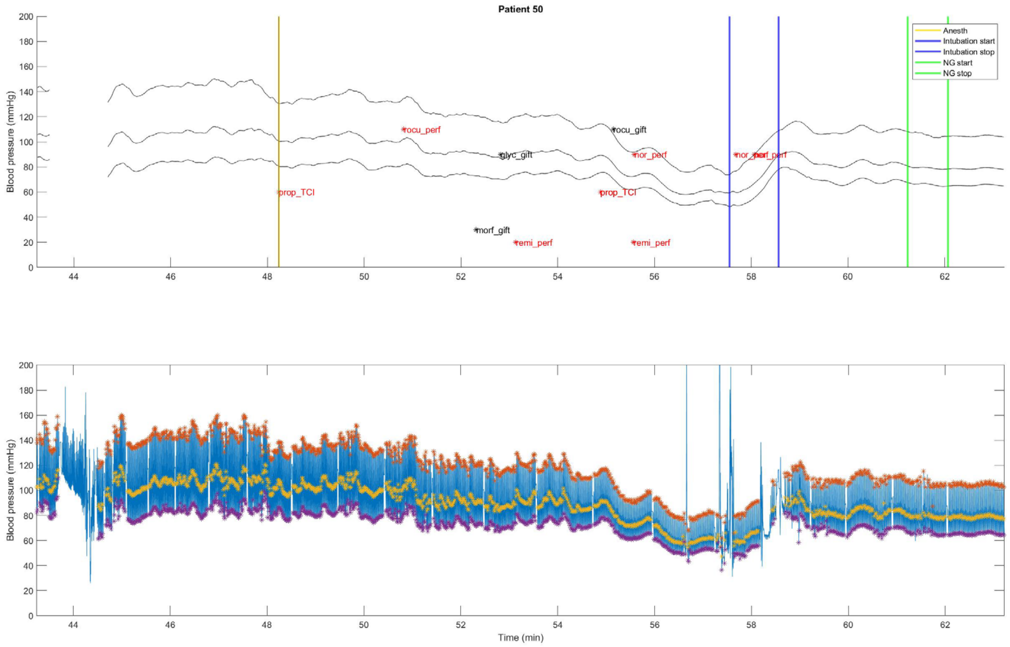
Patient 50
Anesth
Intubation start
Intubation stop
NG start
NG stop
rocu_perf
rocu_gift
glyc_gift
nor_perf
prop_TCI
morf_gift
remi_perf
Blood pressure (mmHg)
Time (min)

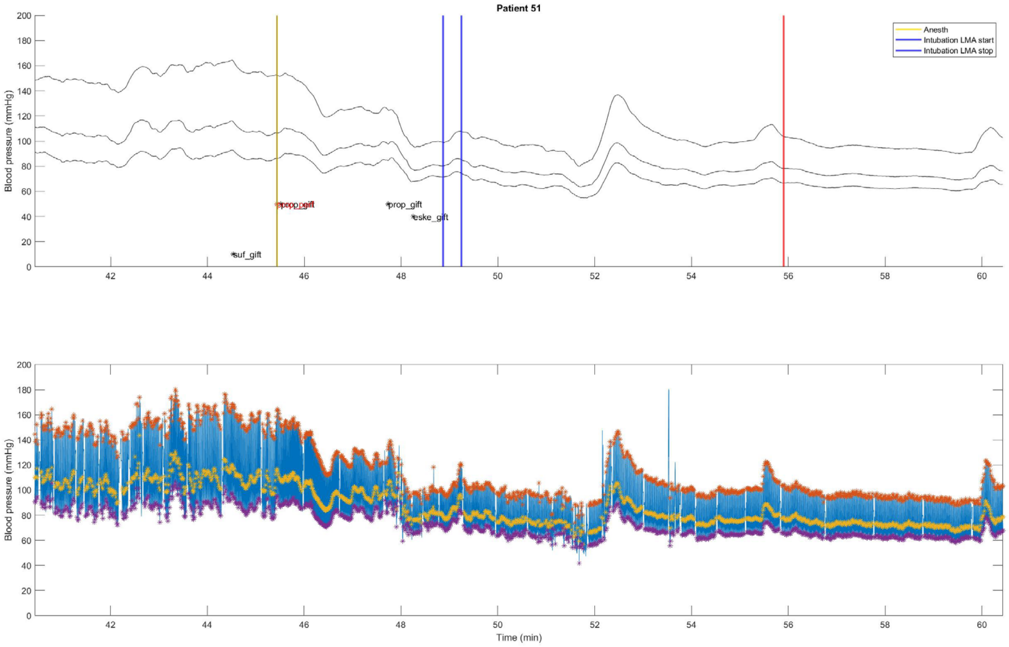
Patient 51
Anesth
Intubation LMA start
Intubation LMA stop
Blood pressure (mmHg)
prop_gift
prop_gift
eske_gift
suf_gift
Time (min)

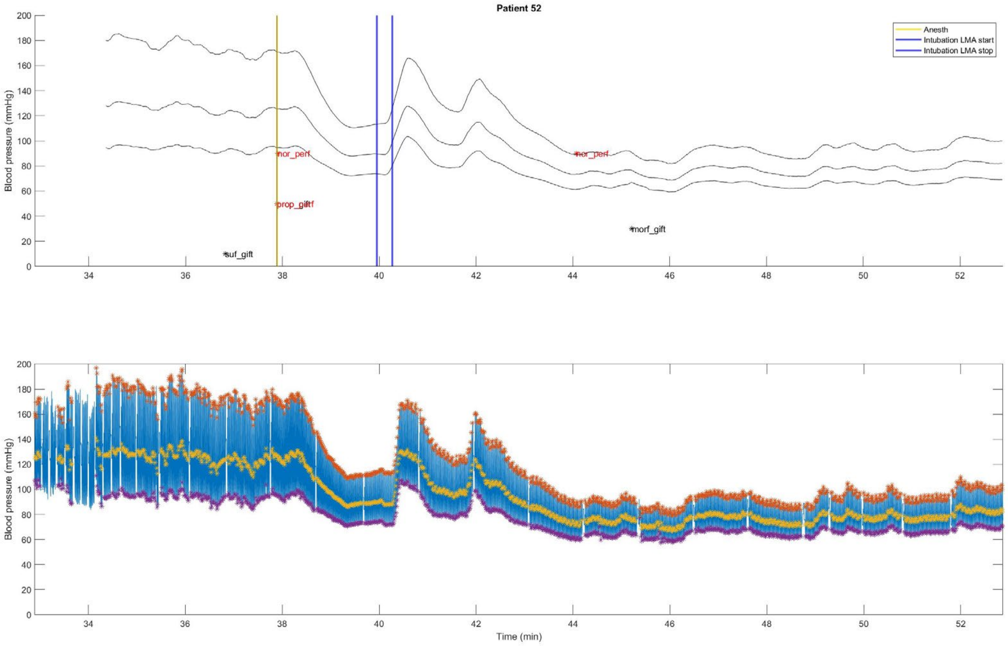
Patient 52
Anesth
Intubation LMA start
Intubation LMA stop
Blood pressure (mmHg)
nor_perf
nor_perf
prop_giftf
morf_gift
suf_gift
Time (min)

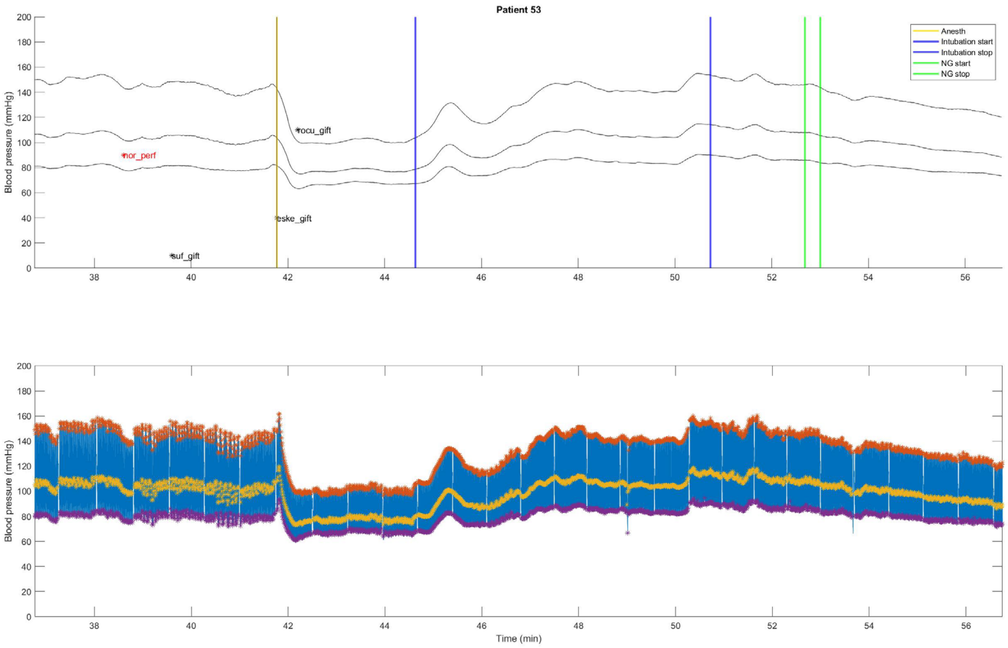
Patient 53
Blood pressure (mmHg)
Time (min)
Anesth
Intubation start
Intubation stop
NG start
NG stop
rocu_gift
nor_perf
eske_gift
suf_gift

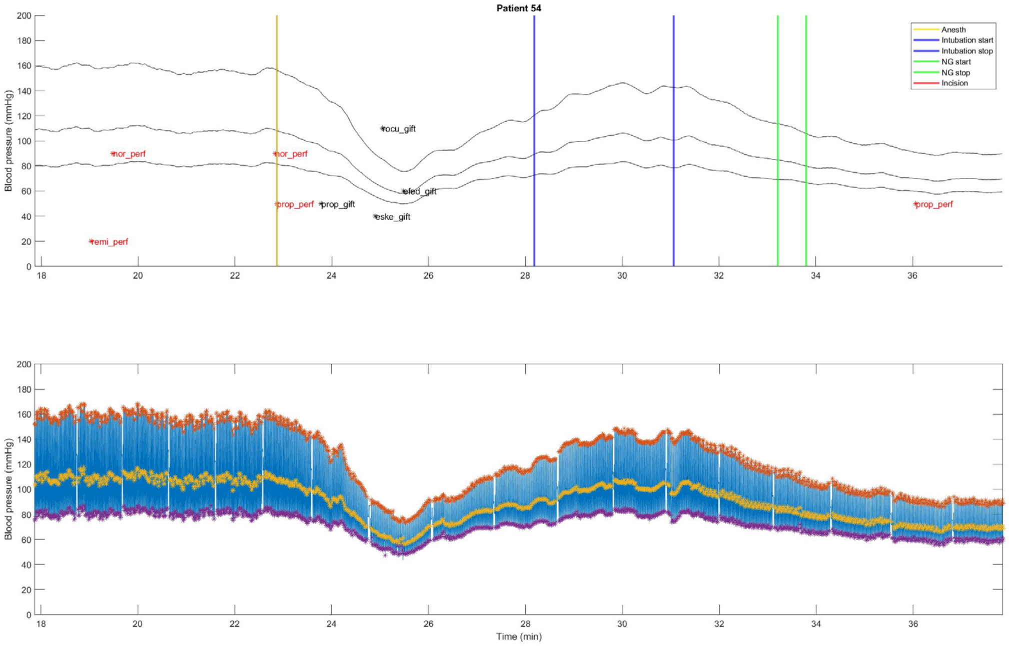
Patient 54
Blood pressure (mmHg)
Time (min)
Anesth
Intubation start
Intubation stop
NG start
NG stop
Incision
rocu_gift
nor_perf
nor_perf
efed_gift
prop_perf
prop_gift
eske_gift
prop_perf
remi_perf

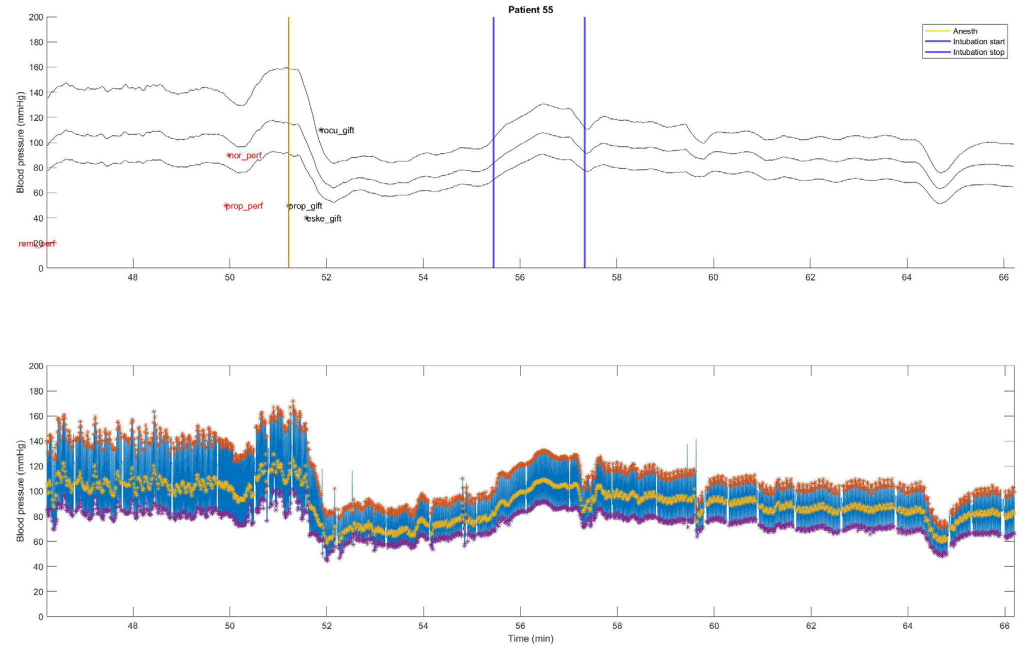
Patient 55
Anesth
Intubation start
Intubation stop
rocu_gift
nor_perf
prop_perf
prop_gift
eske_gift
remi_perf
Blood pressure (mmHg)
Time (min)

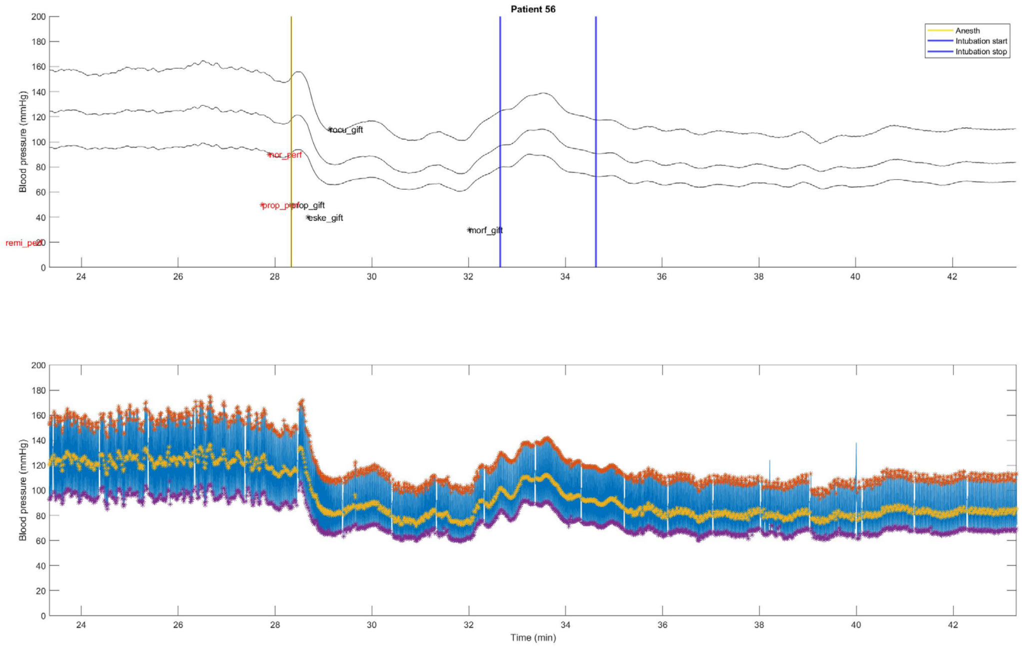
Patient 56
Anesth
Intubation start
Intubation stop
rocu_gift
nor_perf
prop_perf
prop_gift
eske_gift
morf_gift
Blood pressure (mmHg)
Time (min)

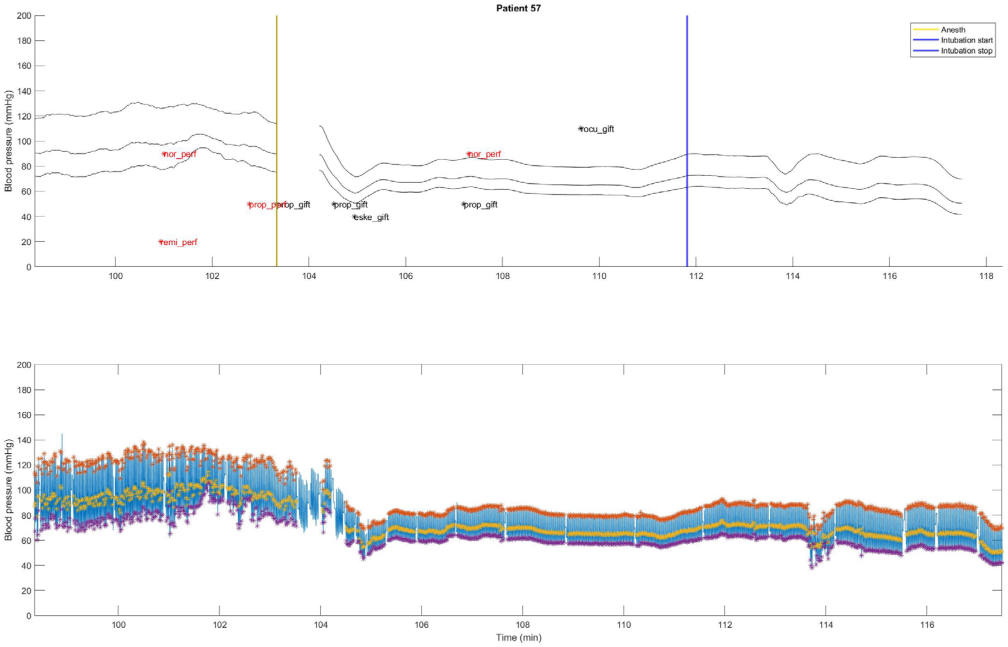
Patient 57
Anesth
Intubation start
Intubation stop
Blood pressure (mmHg)
nor_perf
remi_perf
prop_perf
prop_gift
eske_gift
rocu_gift
Time (min)

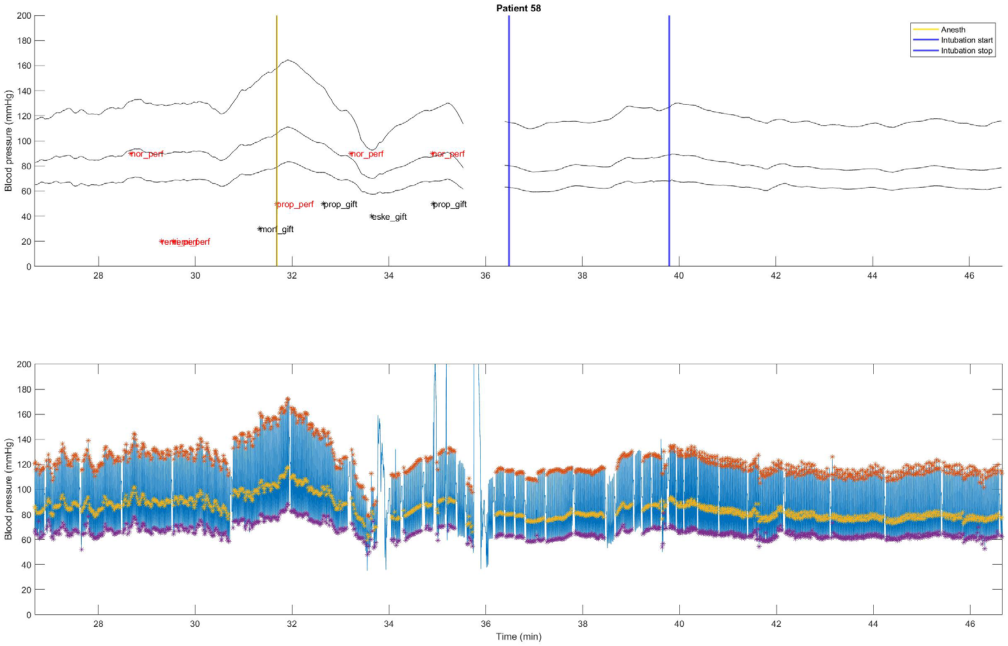
Patient 58
Anesth
Intubation start
Intubation stop
Blood pressure (mmHg)
nor_perf
prop_perf
prop_gift
eske_gift
mor_gift
Time (min)

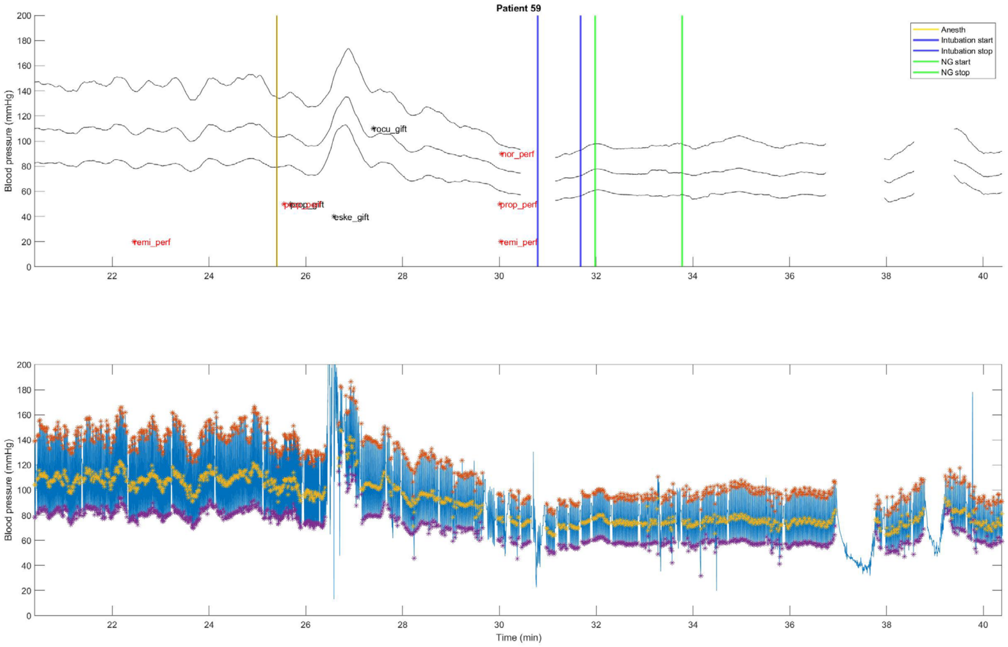
Patient 59
Blood pressure (mmHg)
Anesth
Intubation start
Intubation stop
NG start
NG stop
rocu_gift
nor_perf
prop_gift
prop_perf
eske_gift
remi_perf
Time (min)

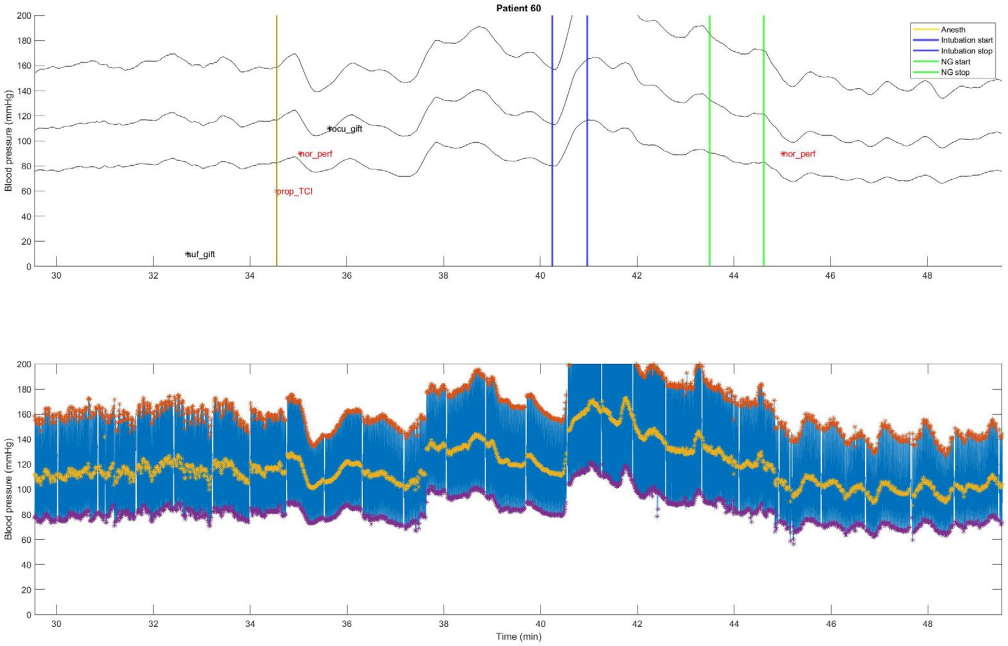
Patient 60
Blood pressure (mmHg)
Anesth
Intubation start
Intubation stop
NG start
NG stop
rocu_gift
nor_perf
prop_TCI
suf_gift
Time (min)

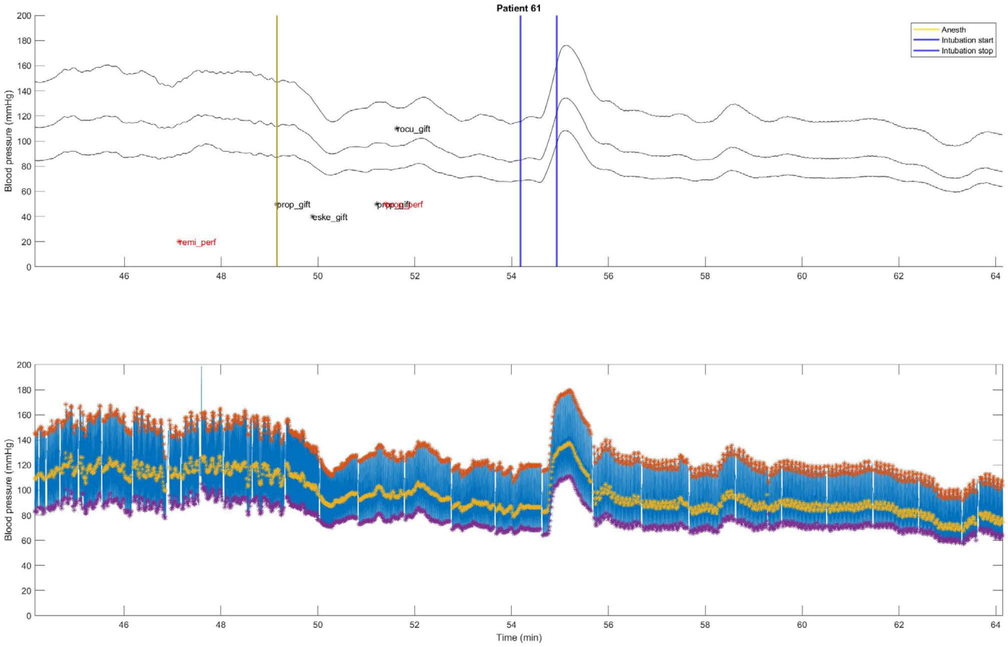

Patient 61
Anesth
Intubation start
Intubation stop
Blood pressure (mmHg)
rocu_gift
prop_gift
eske_gift
remi_perf
Time (min)

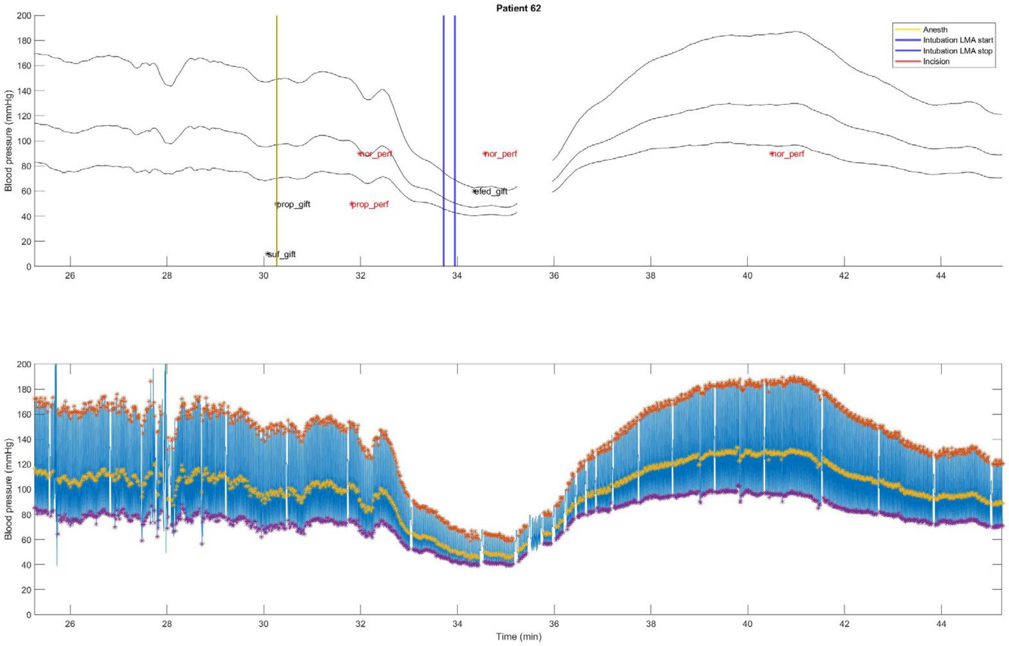

Patient 62
Anesth
Intubation LMA start
Intubation LMA stop
Incision
Blood pressure (mmHg)
nor_perf
efed_gift
prop_gift
prop_perf
suf_gift
Time (min)

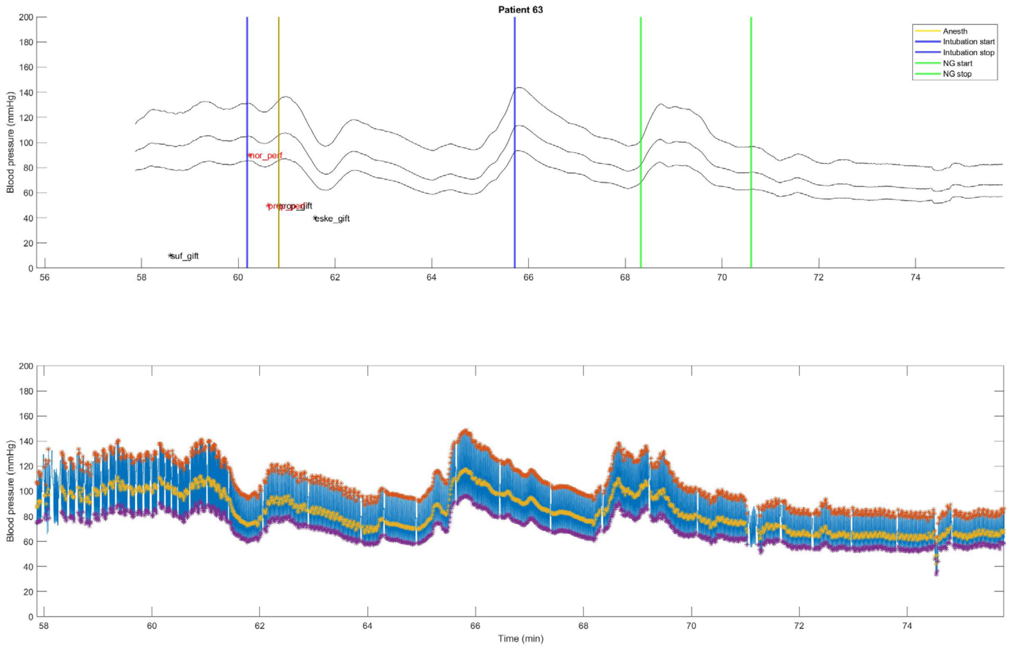
Patient 63
Anesth
Intubation start
Intubation stop
NG start
NG stop
Blood pressure (mmHg)
nor_perf
eske_gift
suf_gift
Time (min)

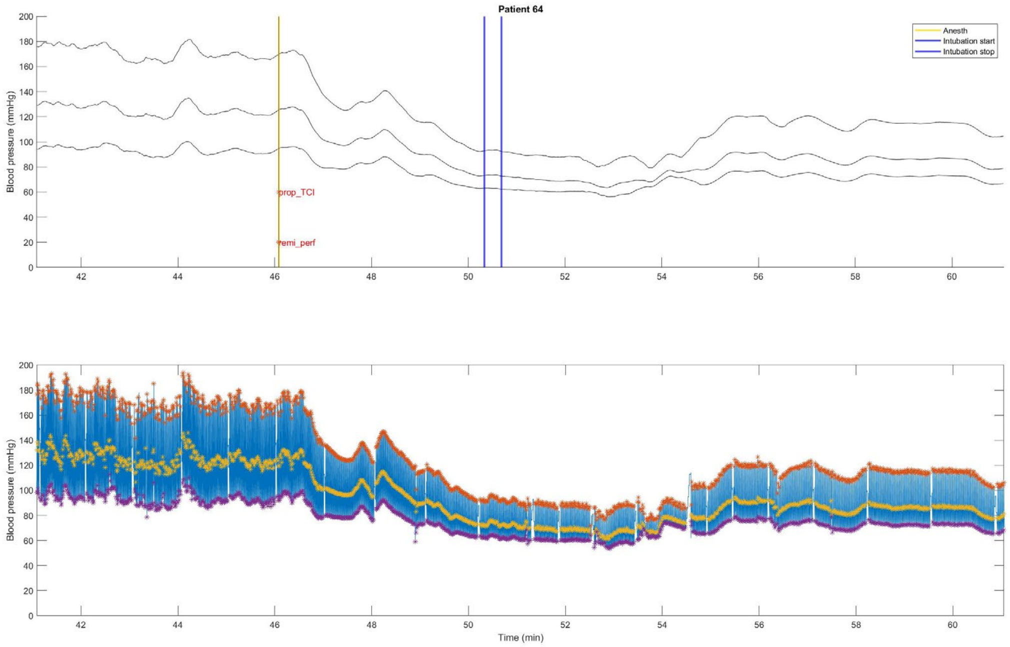
Patient 64
Anesth
Intubation start
Intubation stop
Blood pressure (mmHg)
prop_TCI
remi_perf
Time (min)

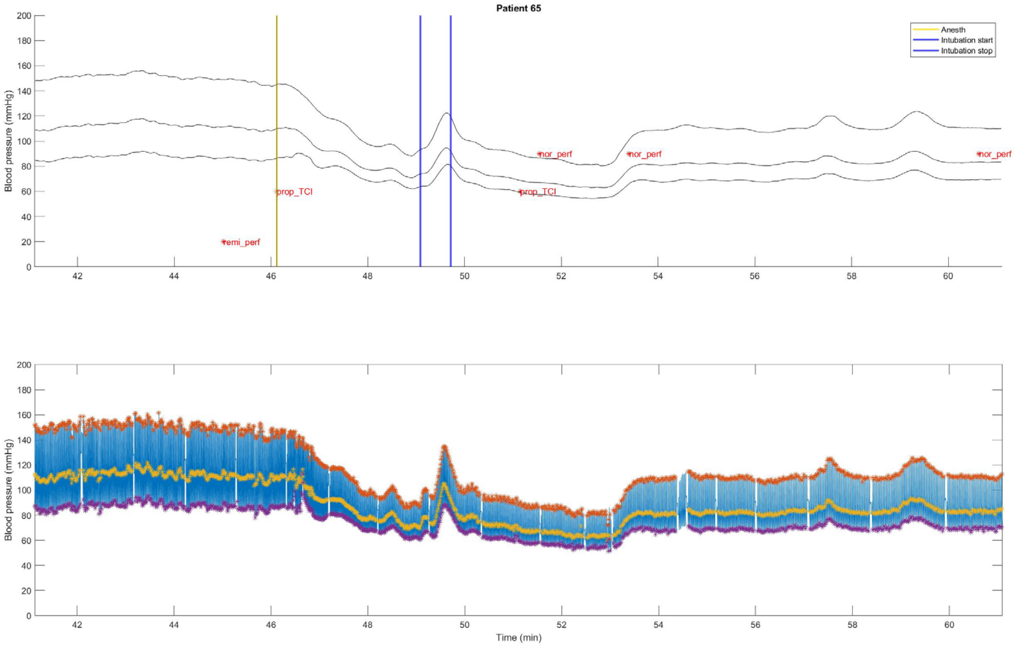
Patient 65
Anesth
Intubation start
Intubation stop
Blood pressure (mmHg)
remi_perf
prop_TCI
nor_perf
Time (min)

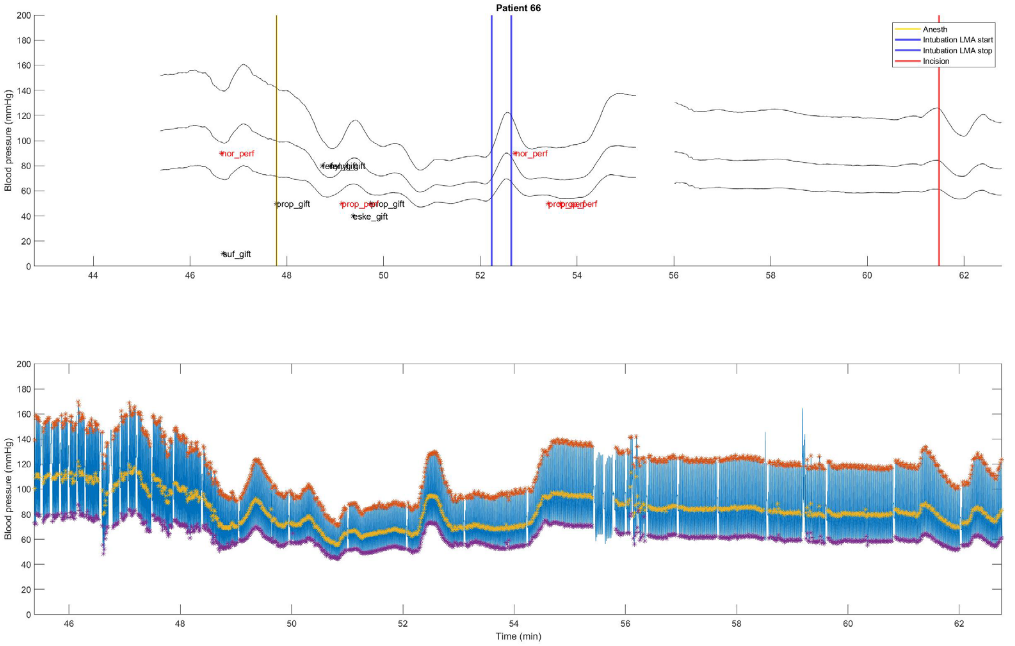
Patient 66
Anesth
Intubation LMA start
Intubation LMA stop
Incision
Blood pressure (mmHg)
nor_perf
prop_gift
suf_gift
eske_gift
Time (min)

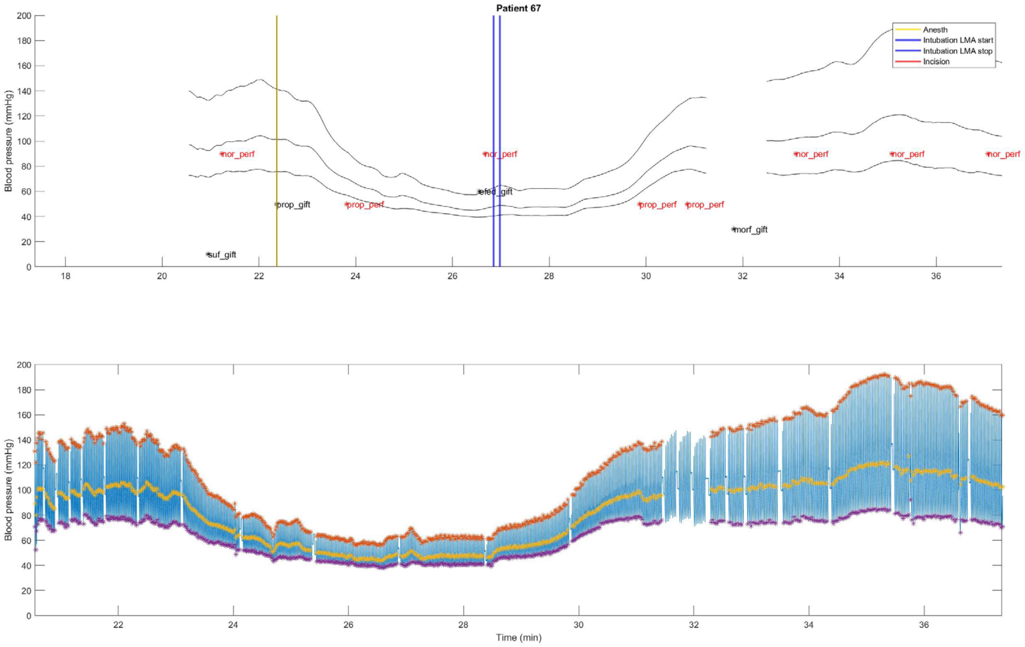
Patient 67
Anesth
Intubation LMA start
Intubation LMA stop
Incision
Blood pressure (mmHg)
nor_perf
prop_gift
prop_perf
efed_gift
morf_gift
suf_gift
Time (min)

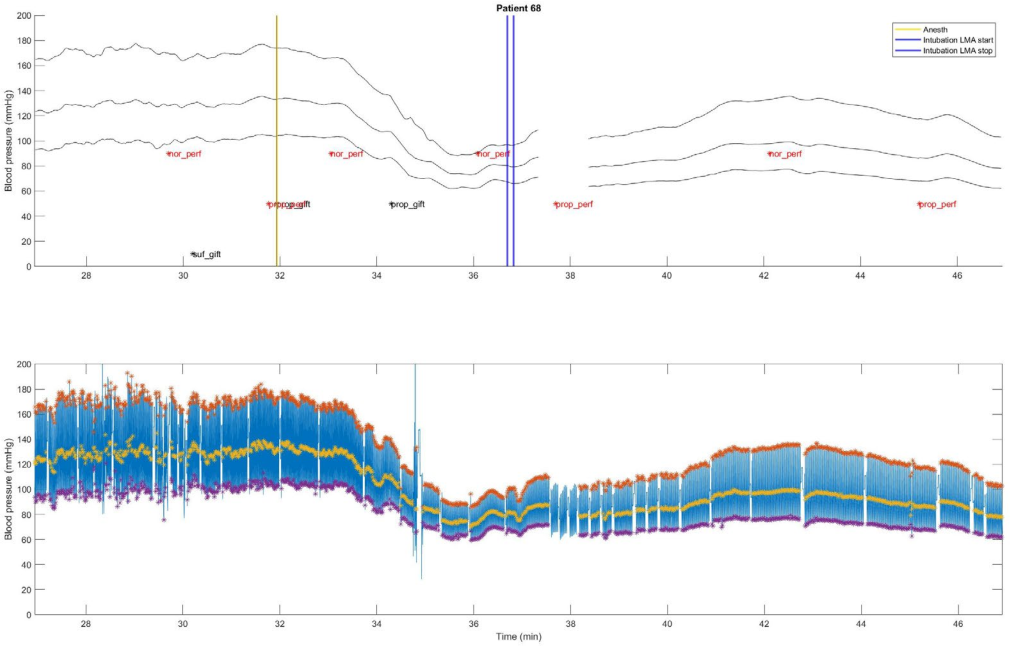
Patient 68
Anesth
Intubation LMA start
Intubation LMA stop
Blood pressure (mmHg)
nor_perf
prop_gift
prop_perf
suf_gift
Time (min)

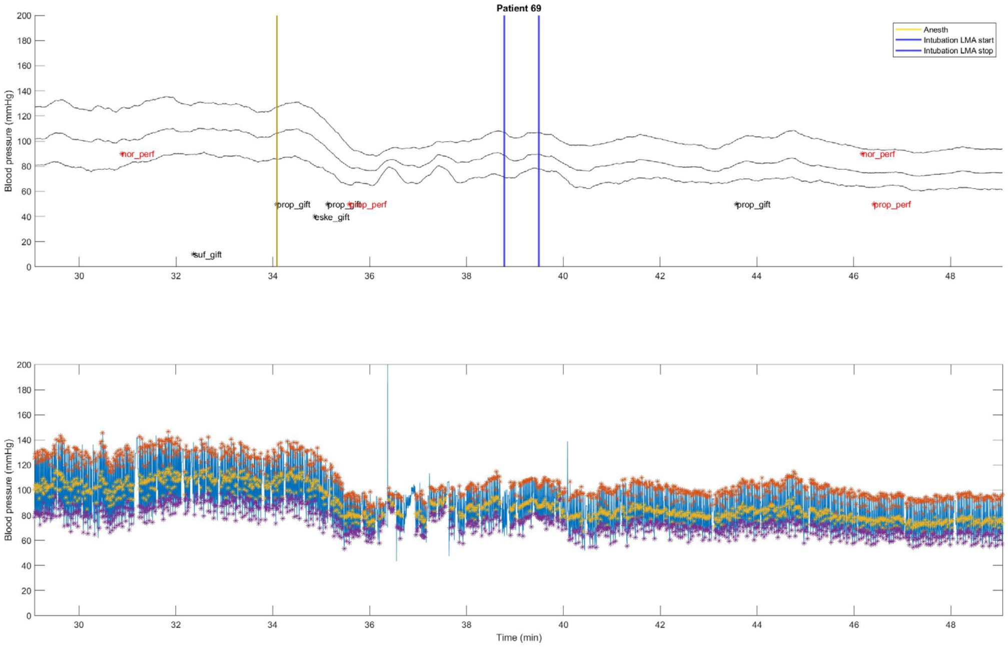
Patient 69
Anesth
Intubation LMA start
Intubation LMA stop
Blood pressure (mmHg)
nor_perf
prop_gift
eske_gift
prop_perf
suf_gift
Time (min)

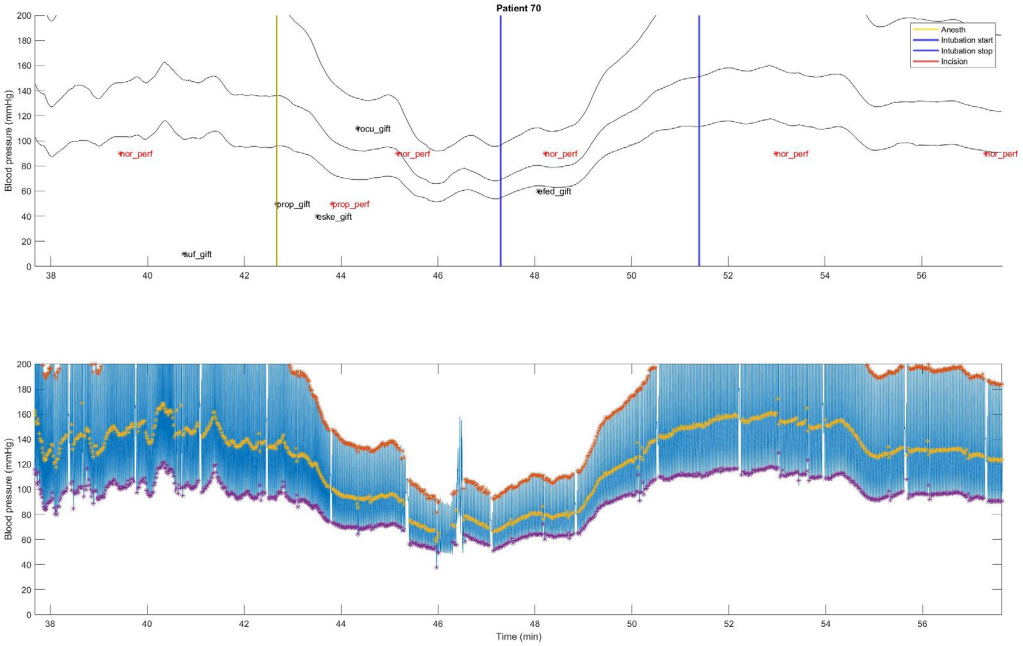
Patient 70
Anesth
Intubation start
Intubation stop
Incision
Blood pressure (mmHg)
nor_perf
rocu_gift
efed_gift
prop_gift
prop_perf
eske_gift
suf_gift
Time (min)

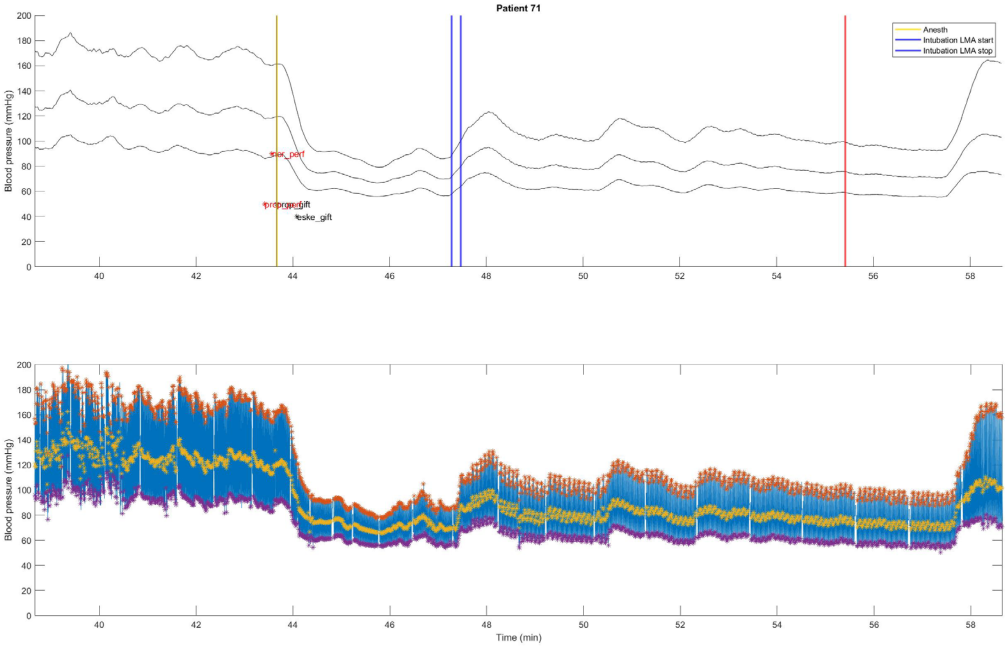
Patient 71
Anesth
Intubation LMA start
Intubation LMA stop
prop_perf
prop_gift
eske_gift
Blood pressure (mmHg)
Time (min)

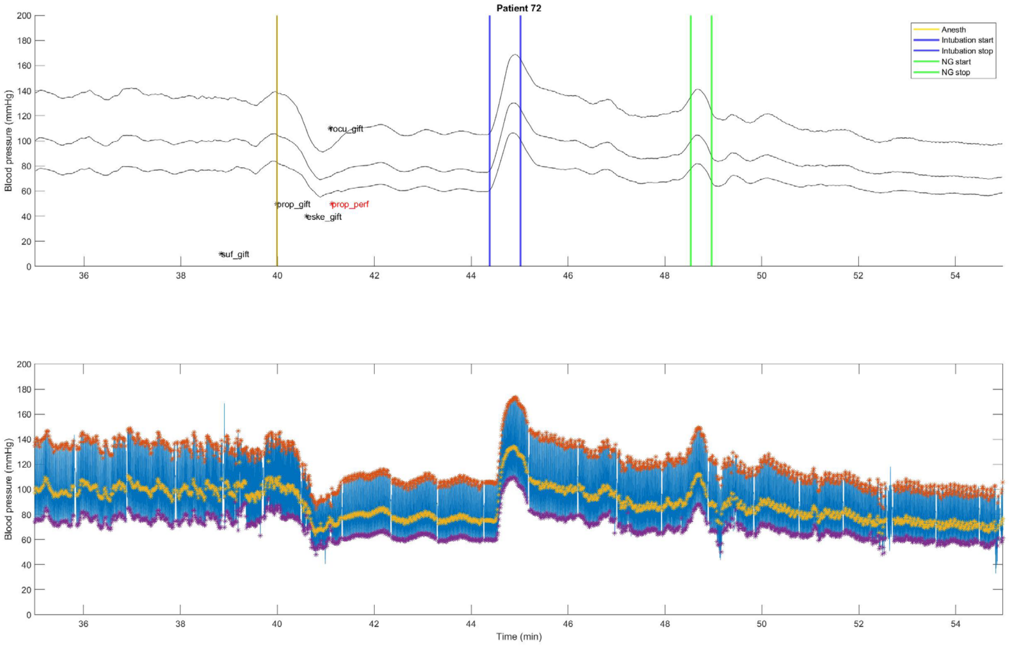
Patient 72
Anesth
Intubation start
Intubation stop
NG start
NG stop
rocu_gift
prop_gift
prop_perf
eske_gift
suf_gift
Blood pressure (mmHg)
Time (min)

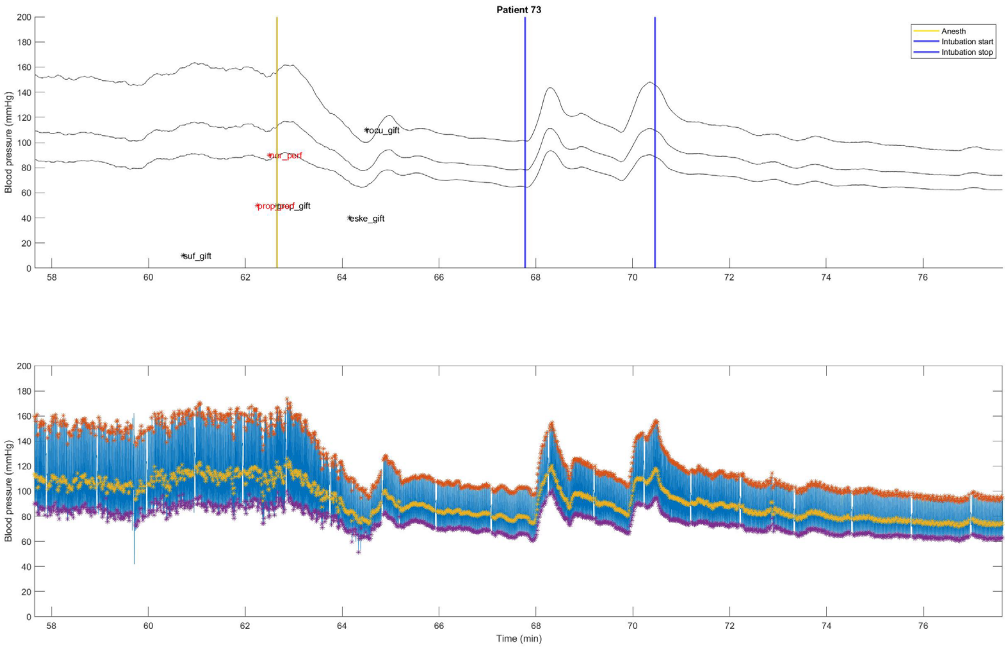
Patient 73
Anesth
Intubation start
Intubation stop
Blood pressure (mmHg)
rocu_gift
eske_gift
suf_gift
Time (min)

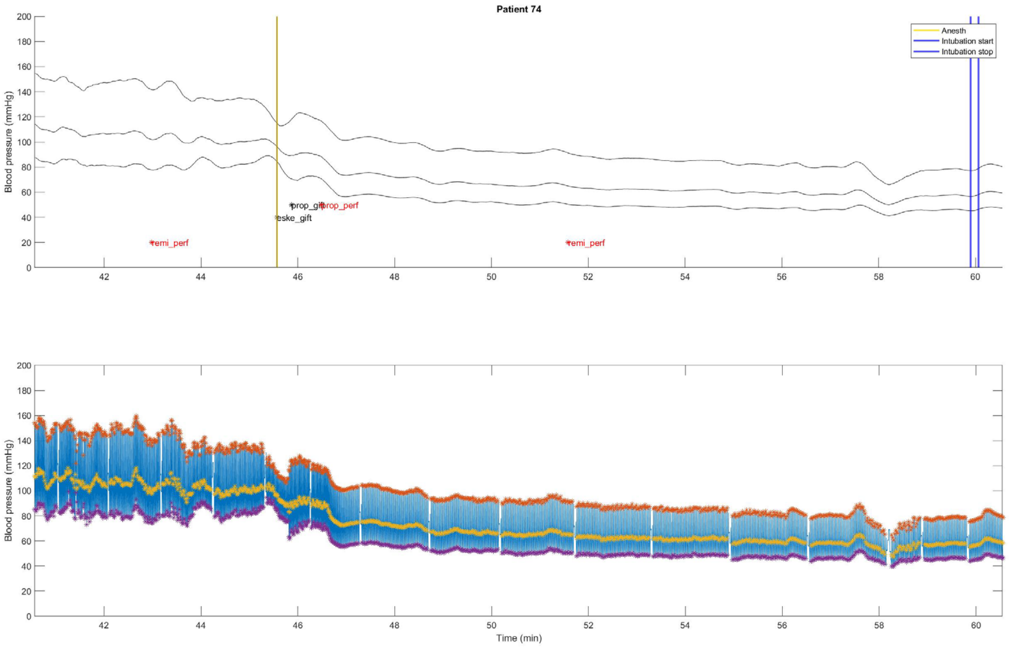
Patient 74
Anesth
Intubation start
Intubation stop
Blood pressure (mmHg)
prop_perf
eske_gift
remi_perf
remi_perf
Time (min)

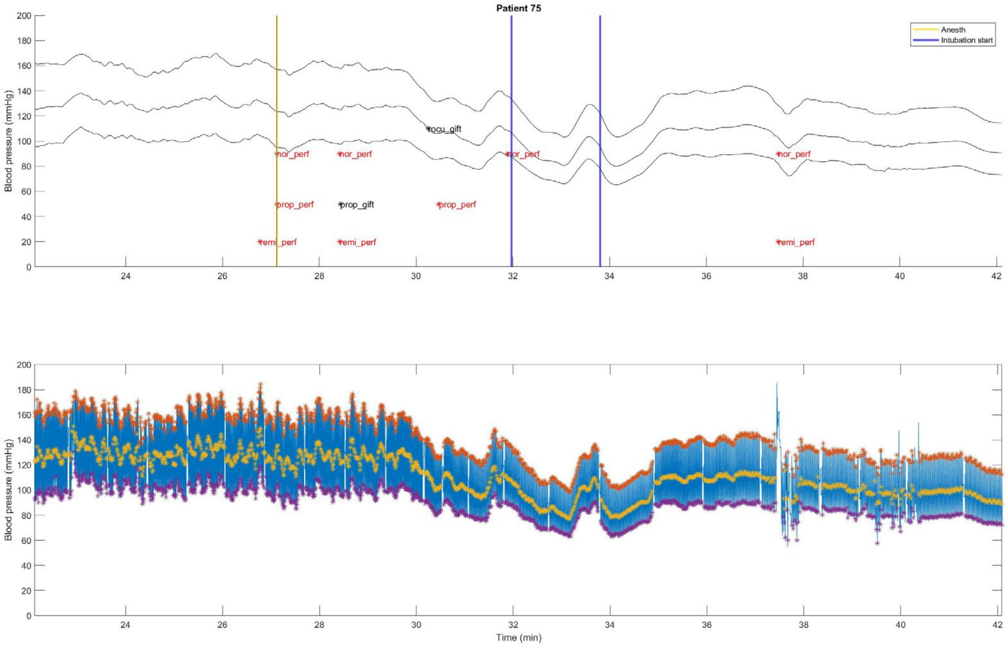
Patient 75
Anesth
Intubation start
Blood pressure (mmHg)
rocu_gift
nor_perf
prop_perf
prop_gift
remi_perf
Time (min)
